# Genome-wide association study of primary tooth eruption identifies pleiotropic loci associated with height and craniofacial distances

**DOI:** 10.1093/hmg/ddt231

**Published:** 2013-05-23

**Authors:** Ghazaleh Fatemifar, Clive J. Hoggart, Lavinia Paternoster, John P. Kemp, Inga Prokopenko, Momoko Horikoshi, Victoria J. Wright, Jon H. Tobias, Stephen Richmond, Alexei I. Zhurov, Arshed M. Toma, Anneli Pouta, Anja Taanila, Kirsi Sipila, Raija Lähdesmäki, Demetris Pillas, Frank Geller, Bjarke Feenstra, Mads Melbye, Ellen A. Nohr, Susan M. Ring, Beate St Pourcain, Nicholas J. Timpson, George Davey Smith, Marjo-Riitta Jarvelin, David M. Evans

**Affiliations:** 1 MRC Centre for Causal Analyses in Translational Epidemiology (CAiTE),; 2 School of Social and Community Medicine and; 3 School of Oral and Dental Sciences, Lower Maudlin Street, Bristol BS1 2LY, UK,; 4 Department of Genomics of Common Disease, School of Public Health, Imperial College London, London W12 ONN, UK,; 5 Oxford Centre for Diabetes, Endocrinology and Metabolism, University of Oxford, Old Road, Oxford OX3 7LJ, UK,; 6 Wellcome Trust Centre for Human Genetics, University of Oxford, Roosevelt Drive, Oxford OX3 7BN, UK,; 7 Department of Paediatrics, Imperial College London, Norfolk Place, London W2 1PG, UK,; 8 Musculoskeletal Research Unit, School of Clinical Sciences, University of Bristol, Southmead Hospital, Bristol BS10 5NB, UK,; 9 Department of Applied Clinical Research and Public Health, Cardiff University, Cardiff CF14 4XY, UK,; 10 Institute of Health Sciences, PO Box 8000,; 11 Department of Clinical Sciences/Obstetrics and Gynecology, PO Box 5000,; 12 Institute of Dentistry, PO Box 5281,; 13 Department of Oral Development and Orthodontics, Institute of Dentistry, PO Box 5281 and; 14 Biocenter Oulu, PO Box 5000, University of Oulu, FIN-90014 Oulu, Finland,; 15 Department of Lifecourse and Services, National Institute for Health and Welfare, PO Box 30, FIN-00271, Finland,; 16 Unit of General Practice, PO Box 22 and; 17 Oral and Maxillofacial Department, PO Box 22,Oulu University Hospital, Oulu FIN-90221, Finland,; 18 Institute of Dentistry, University of Eastern Finland, PO Box 1627, FIN-70211, Kuopio, Finland,; 19 Oral and Maxillofacial Department, Kuopio University Hospital, PO Box 1627, FIN-70211, Kuopio, Finland,; 20 Department of Epidemiology and Public Health, University College London, London WC1E 6BT, UK,; 21 Department of Epidemiology Research, Statens Serum Institut, Copenhagen 2300, Denmark,; 22 Department of Public Health, Section for Epidemiology, Aarhus University, Aarhus 8000C, Denmark,; 23 Department of Epidemiology and Biostatistics, School of Public Health, MRC-HPA Centre for Environment and Health, Faculty of Medicine, Imperial College London, UK and; 24 Unit of Primary Care, PO Box 20, Oulu University Hospital, Kajaanintie 50, FI-90220, 90029 OYS, Finland

## Abstract

Twin and family studies indicate that the timing of primary tooth eruption is highly heritable, with estimates typically exceeding 80%. To identify variants involved in primary tooth eruption, we performed a population-based genome-wide association study of ‘age at first tooth’ and ‘number of teeth’ using 5998 and 6609 individuals, respectively, from the Avon Longitudinal Study of Parents and Children (ALSPAC) and 5403 individuals from the 1966 Northern Finland Birth Cohort (NFBC1966). We tested 2 446 724 SNPs imputed in both studies. Analyses were controlled for the effect of gestational age, sex and age of measurement. Results from the two studies were combined using fixed effects inverse variance meta-analysis. We identified a total of 15 independent loci, with 10 loci reaching genome-wide significance (*P* < 5 × 10^−8^) for ‘age at first tooth’ and 11 loci for ‘number of teeth’. Together, these associations explain 6.06% of the variation in ‘age of first tooth’ and 4.76% of the variation in ‘number of teeth’. The identified loci included eight previously unidentified loci, some containing genes known to play a role in tooth and other developmental pathways, including an SNP in the protein-coding region of *BMP4* (rs17563, *P* = 9.080 × 10^−17^). Three of these loci, containing the genes *HMGA2*, *AJUBA* and *ADK*, also showed evidence of association with craniofacial distances, particularly those indexing facial width. Our results suggest that the genome-wide association approach is a powerful strategy for detecting variants involved in tooth eruption, and potentially craniofacial growth and more generally organ development.

## INTRODUCTION

Primary tooth eruption is a complex and highly regulated process through which primary teeth enter the mouth and become visible. Prior to eruption, mononuclear cells move to the dental follicle and fuse to form osteoclasts. These osteoclasts subsequently resorb alveolar bone and in doing so form an eruption pathway through which the primary dentition can then emerge (1).

Twin studies have provided insight into the genetic control of primary tooth eruption during childhood. The ‘Dental Development and Oral Health of Australian Twins and Their Families’ was a longitudinal study of 98 sets of twins of European ancestry aged between 1 and 3 years of age that aimed to assess the degree to which variation in tooth eruption was due to genetic factors. Although there was no statistically significant difference in eruption times between zygosity and the sexes, there was strong genetic control with regard to the timing of primary incisor eruption with an estimated heritability of ∼ 82 to 94% in males, and 71 to 96% in females (2).

The majority of current knowledge regarding the genetics of tooth eruption and tooth development has been acquired from studies involving transgenic mice and other model organisms, including fish and reptiles, as well as from clinical genetic studies of humans with congenital disorders in which dental abnormalities are a feature. For example, studies in mice have implicated a host of signalling pathways as being critical in proper tooth eruption and development, including those involving the gene families *Bmp*, *Eda*, *Fgf*, *Shh* and *Wnt*, among others (3–5). These pathways are integrated at several stages of the tooth development process and the network appears to be highly conserved evolutionarily across species (4). Disruption of these pathways typically results in severe aberrations of dentition, including tooth agenesis or arrest in the early stages of tooth development (3).

Population-based genome-wide association studies (GWASs) of tooth eruption in children have the capacity to provide complementary information to these studies, by identifying common genetic variation which is associated with non-pathological differences in the timing of tooth eruption between individuals. Loci implicated by GWASs may not necessarily be the same as those that have been identified in molecular studies or be associated with abnormalities, but rather may reflect variation in genes important in more subtle aspects of tooth development, including differences in the timing of tooth eruption or perhaps even genetic variation important in more generalized aspects of growth and development.

In a previous genome-wide meta-analysis of primary tooth eruption, we identified five loci associated with ‘age at first tooth’ and ‘number of teeth’ at 1 year of age at genome-wide levels of significance, and a further five at suggestive levels of significance (6). Many of these loci contained genes previously implicated in tooth or other organ development. A more recent GWAS of secondary tooth eruption identified two of the same loci as well as two others containing the genes *ADK* and *CACNA1S*/*TMEM9* (7). What was particularly striking about both studies was the number of loci displaying large effect sizes. Typically, GWASs of quantitative traits require tens of thousands of individuals to identify common variants of small effect. However, the tooth eruption phenotype appears to be influenced by some loci of comparably large effect (i.e. >1% of the phenotypic variance), implying that the genome-wide study of primary tooth eruption might be a powerful strategy not only at detecting variants involved in dentition, but also SNPs that may exert pleiotropic actions on other aspects of growth and development.

In order to identify novel variants involved in primary tooth eruption, we doubled the size of our previous population-based genome-wide association meta-analysis, increasing our sample to include 5998 and 6609 individuals from the Avon Longitudinal Study of Parents and Children (ALSPAC) for ‘age at first tooth’ and ‘number of teeth’, and a further 5403 individuals from the 1966 Northern Finland Birth Cohort (NFBC1966). SNPs that met the criteria for genome-wide significance (*P* < 5 × 10^−8^) were then assessed for association with other related phenotypes, including measures of craniofacial shape and size, secondary tooth eruption, and height. The aim of our study was to (i) identify novel genetic loci associated with tooth eruption, and (ii) to investigate whether variants associated with tooth development exhibited pleiotropic effects on growth in general. Specifically, we examined the relationship between tooth-associated loci and eruption of secondary teeth, height, craniofacial size and shape, as well as possible relationships between known height-associated loci and tooth eruption.

## RESULTS

A total of 2 446 724 SNPs common to both studies were tested for association with ‘age at first tooth’ and ‘number of teeth at one year’. All analyses were adjusted for gestational age, sex and age, where appropriate (see Materials and Methods). Results from the two studies were combined using fixed effects inverse variance meta-analysis, where effect size estimates are weighted according to the inverse of their standard errors. Q–Q plots indicated little inflation of the test statistics in the individual cohorts and for the meta-analysis overall (‘Age at first tooth’ _LAMBDA ALSPAC_ = 1.04; ‘Age at first tooth’_LAMBDA NFBC1966_ = 1.05; _LAMBDA META_ = 1.07; ‘Number of teeth’: _LAMBDA ALSPAC_ = 1.02; _LAMBDA NFBC1966_ = 1.04; _LAMBDA META_ = 1.06) (Supplementary Material, Fig. S1). The genomic inflation factor *λ* is well known to increase with sample size; we, therefore, also calculated *λ*_1000_ values (8) for the ‘Age at first tooth’ (*λ*_1000_ = 1.01) and ‘Number of teeth’ (*λ*_1000_ = 1.00) meta-analyses. Both values are consistent with little latent population stratification or other systematic biases affecting our results.

**Figure 1.**
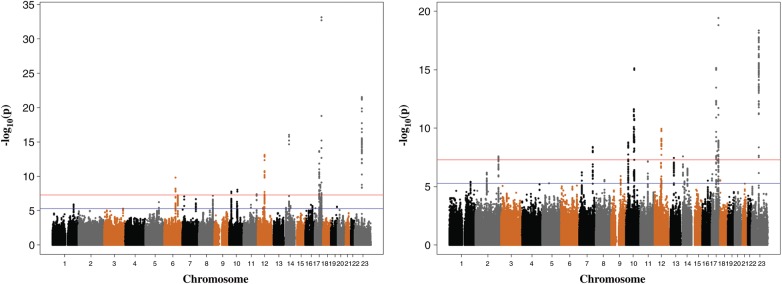
Manhattan plots for meta-analysis of ‘age at first tooth’ and ‘number of teeth’, respectively

We identified 10 loci reaching genome-wide significance (*P* < 5 × 10^−8^) for ‘age at first tooth’ and a further 11 loci for ‘number of teeth’, giving a total of 15 independent loci (Fig. 1). The full GWAS results corresponding to Figure 1 are available from the *Human Molecular Genetics* website. Table 1 shows the top-ranking SNPs for each phenotype at each locus. Eight of these loci are novel associations; the top SNPs at these loci are rs17563 (*BMP4*), rs10740993 (*CACNB2*), rs4937076 (*CDON*), rs1799922 (*CALU*/*OPN1SW*), rs997154 (*AJUBA/C14orf93*), rs7924176 (*ADK*), rs412000 (*TEX14/RAD51C*) and rs9316505 (*DLEU7*). Four of the loci identified confirm previously reported genes/regions (6) (*KCNJ2*, *MSRB3*, *IGF2BP1* and *EDA*). Furthermore, we detected genome-wide significance for the variant rs17101923 in the *HMGA2* region (‘number of teeth’ *P* = 1.1 × 10^−10^, Table 1), rs10932688 in the *2q35* region and the rs6568401 variant in the *6q21* region, which were identified at suggestive levels of significance in a previous study (6). We also note that SNPs at the *RAD51L1* locus reported as genome-wide significant for association with ‘number of teeth’ in Pillas *et al*. (6) did not meet the 5 × 10^−8^ threshold in this study, although there was still suggestive evidence for association at this locus [‘Age at first tooth’ (rs17105278): *P* = 2.1 × 10^−6^; ‘Number of teeth’(rs1956529): *P* = 6.4 × 10^−7^].

**Table 1.** Fifteen loci identified at genome-wide significance in meta-analysis of ‘age at first tooth’ or ‘number of teeth’ in ALSPAC and NFBC1966

Marker	Gene region/locus	Chromosome	Base position	A1/A2	Effect ALSPAC	SE ALSPAC	% var alspac	*P*-value ALSPAC	Effect NFBC	SE NFBC	% var nfbc	*P*-value NFBC	Effect meta	SE meta	*P*-value meta
Age at first tooth															
rs10932688	*2q35*	2	217 571 726	**G**/C	−0.107	0.048	0.05	2.7 × 10^−2^	−0.106	0.037	0.13	4.0 × 10^−3^	−0.106	0.029	2.9 × 10^−4^
rs6568401	*6q21*	6	106 295 511	**C**/T	−0.228	0.049	0.33	3.1 × 10^−6^	−0.168	0.037	0.37	7.1 × 10^−6^	−0.190	0.030	1.5 × 10^−10^
rs1799922	*CALU/OPN1SW*	7	128 202 431	**T**/G	−0.148	0.044	0.16	7.8 × 10^−4^	−0.135	0.037	0.23	3.2 × 10^−4^	−0.140	0.029	8.8 × 10^−7^
rs10740993	*CACNB2*	10	18 482 488	**C**/T	−0.175	0.043	0.24	4.7 × 10^−5^	−0.118	0.035	0.19	6.7 × 10^−4^	−0.141	0.0271	1.9 × 10^−7^
rs7924176	*ADK VCL AP3M1*	10	75 965 795	**A**/G	−0.261	0.043	0.58	1.2 × 10^−9^	−0.081	0.037	0.06	2.5 × 10^−2^	−0.167	0.028	1.8 × 10^−8^
rs4937076	*CDON*	11	125 331 912	**A**/G	−0.186	0.043	0.27	1.8 × 10^−5^	−0.127	0.035	0.21	3.3 × 10^−4^	−0.150	0.027	4.0 × 10^−8^
rs12229918	*MSRB3*	12	64 048 325	**C**/G	−0.273	0.044	0.60	7.3 × 10^−10^	−0.176	0.039	0.37	5.3 × 10^−6^	−0.218	0.029	7.3 × 10^−14^
rs17101923	*HMGA2*	12	64 624 469	**G**/T	−0.282	0.053	0.44	9.99 × 10^−8^	−0.170	0.041	0.30	3.5 × 10^−5^	−0.212	0.033	6.3 × 10^−11^
rs9316505	*DLEU7*	13	50 288 599	**A**/G	−0.122	0.043	0.10	4.8 × 10^−3^	−0.095	0.038	0.08	1.2 × 10^−2^	−0.107	0.028	1.8 × 10^−4^
rs997154	*AJUBA/C14orf93*	14	22 534 322	**G**/A	−0.132	0.051	0.08	1.0 × 10^−2^	−0.142	0.038	0.23	2.2 × 10^−4^	0.138	0.031	6.9 × 10^−6^
rs17563	*BMP4*	14	53 487 272	**G**/A	−0.339	0.043	0.98	4.9 × 10^−15^	−0.160	0.038	0.29	2.9 × 10^−5^	−0.239	0.029	9.1 × 10^−17^
rs1994969	*IGF2BP1*	17	44 435 430	**T**/G	−0.211	0.043	0.36	1.0 × 10^−6^	−0.203	0.035	0.61	4.5 × 10^−9^	−0.206	0.027	2.3 × 10^−14^
rs412000	*TEX14/RAD51C*	17	54 064 057	**C**/G	−0.752	0.0431	0.24	5.0 × 10−^5^	−0.157	0.035	0.34	8.2 × 10−^6^	−0.1641	0.027	1.7 × 10^−9^
rs8080944	*KCNJ2 KCNJ16*	17	65 697 181	**A**/G	−0.378	0.045	1.14	2.8 × 10^−17^	−0.317	0.036	1.45	2.0 × 10^−18^	−0.341	0.028	7.6 × 10^−34^
rs11796357	*FAM155E - EDA*	X	68 581 724	**G**/A	−0.290	0.041	0.81	1.1 × 10^−12^	−0.222	0.033	0.85	2.0 × 10^−11^	−0.250	0.026	3.1 × 10^−22^
Number of teeth															
rs10932688	*2q35*	2	217 571 726	**G**/C	0.118	0.035	0.09	6.4 × 10^−4^	0.173	0.038	0.39	5.8 × 10^−6^	0.143	0.026	2.5 × 10^−8^
rs6568401	*6q21*	6	106 295 511	**C**/T	0.156	0.035	0.25	8.4 × 10^−6^	0.058	0.039	0.03	1.4 × 10^−1^	0.112	0.026	1.6 × 10^−5^
rs1799922	*CALU/OPN1SW*	7	128 202 431	**T**/G	0.138	0.031	0.28	1.0 × 10^−5^	0.152	0.039	0.25	9.6 × 10^−5^	0.144	0.024	4.0 × 10^−9^
rs10740993	*CACNB2*	10	18 482 488	**C**/T	0.132	0.031	0.26	1.7 × 10^−5^	0.153	0.036	0.3	2.2 × 10^−5^	0.141	0.023	1.7 × 10^−9^
rs7924176	*ADK VCL AP3M1*	10	75 965 795	**A**/G	0.248	0.031	0.94	8.8 × 10^−16^	0.109	0.038	0.19	3.9 × 10^−3^	0.193	0.024	7.8 × 10^−16^
rs4937076	*CDON*	11	125 331 912	**A**/G	0.082	0.031	0.11	7.5 × 10^−3^	0.121	0.037	0.19	9.9 × 10^−4^	0.098	0.024	3.1 × 10^−5^
rs12229918	*MSRB3*	12	64 048 325	**C**/G	0.144	0.032	0.30	5.3 × 10^−6^	0.158	0.041	0.21	1.1 × 10^−4^	0.149	0.025	2.3 × 10^−9^
rs17101923	*HMGA2*	12	64 624 469	**G**/T	0.191	0.037	0.36	3.3 × 10^−7^	0.169	0.043	0.29	7.3 × 10^−5^	0.182	0.028	1.1 × 10^−10^
rs9316505	*DLEU7*	13	50 288 599	**A**/G	0.132	0.031	0.24	1.5 × 10^−5^	0.133	0.039	0.17	6.2 × 10^−4^	0.133	0.024	3.4 × 10^−8^
rs997154	*AJUBA/C14orf93*	14	22 534 322	**G**/A	0.124	0.037	0.10	7.0 × 10^−4^	0.181	0.040	0.35	5.8 × 10^−6^	0.150	0.027	2.6 × 10^−8^
rs17563	*BMP4*	14	53 487 272	**G**/A	0.117	0.031	0.16	1.7 × 10^−4^	0.039	0.040	0.03	3.3 × 10^−1^	0.087	0.025	3.6 × 10^−4^
rs1994969	*IGF2BP1*	17	44 435 430	**T**/G	0.189	0.031	0.50	8.5 × 10^−10^	0.190	0.036	0.54	1.6 × 10^−7^	0.190	0.024	7.2 × 10^−16^
rs412000	*TEX14/RAD51C*	17	54 064 057	**C**/G	0.105	0.031	0.12	7 × 10^−4^	0.098	0.037	0.10	7.4 × 10^−3^	0.102	0.024	1.6 × 10^−5^
rs8080944	*KCNJ2 KCNJ16*	17	65 697 181	**A**/G	0.192	0.032	0.54	1.6 × 10^−9^	0.317	0.036	0.92	1.9 × 10^−18^	0.221	0.024	1.5 × 10^−19^
rs11796357	*FAM155E - EDA*	X	68 581 724	**G**/A	0.175	0.029	0.56	2.5 × 10^−9^	0.231	0.035	0.74	2.2 × 10^−11^	0.199	0.022	6.9 × 10^−19^

SNPs showing a genome-wide significance of *P* < 5 × 10^−8^ in the meta-analysis. The *P*-value for each cohort is corrected for gestational age and sex. ALSPAC was also corrected for age at measurement. *P*-values from the meta-analysis were calculated using a fixed effects inverse variance model. All alleles refer to the forward strand. Positions of SNPs reported correspond to HapMap release II build 36. The effect allele A1 (bold) is defined as the allele associated with faster tooth eruption and an increase in the number of teeth.

Each SNP that reached genome-wide significance explained only a small fraction of the overall phenotypic variation in ‘age at first tooth’ (0.05–1.14%, ALSPAC; 0.06–1.45%, NFBC1966) and ‘number of teeth’ (0.09–0.94%, ALSPAC; 0.03–0.92%, NFBC1966). Pooling together the effects of the top SNPs at the genome-wide significant loci (Table 1) into a single allelic score explained 6.06% of the overall phenotypic variation in ‘age at first tooth’ and 4.76% of the variation in ‘number of teeth’. We also report loci displaying suggestive levels of association (Supplementary Material, Tables S1 and S2), 5 × 10^−6^ > *P* > 5 × 10^−8^, which included SNPs in the *TMEM9* region that were reported as genome-wide significant in the study of secondary dentition by Geller *et al*. (7).

Supplementary Material, Figures S2 and S3 show LocusZoom plots of regression analyses for ‘age at first tooth’ and ‘number of teeth’, respectively, at each genome-wide significant locus after meta-analysis (9). For most loci, there appeared to be evidence of secondary signals independent of the lead SNP at the locus. To quantify the evidence for independent secondary signals, we first calculated the effective number of statistical tests in each region using Nyholt's procedure (10). For each locus, we estimated the threshold for a family-wise error rate of 5% by dividing alpha = 0.05 by the corresponding number of effective tests in that region, and used this threshold for declaring a secondary signal as significant. These thresholds as well as the strongest *P*-value in each region after conditioning on the lead SNP are presented in Supplementary Material, Table S3. These analyses showed that there were likely to be independent secondary signals at rs11077486 (*KCNJ2 KCNJ160*), rs2520397 (*FAM155E–EDA*), rs1951867 (*BMP4*), rs1472259 (*HMGA2*) and rs8069452 (*IGF2BP1*) for ‘age at first tooth’ and rs9788982 (*KCNJ2 KCNJ160*), rs2804391 (*FAM155E–EDA*), rs1458991 (*BMP4*), rs9894411 (*IGF2BP1*), rs1976274 (*MSRB3*) and rs1472259 (*HMGA2*) for ‘number of teeth’ (Supplementary Material, Table S3).

We next investigated whether the SNPs at our top loci have pleiotropic effects, specifically whether they are associated with both primary tooth and craniofacial development. A recent GWAS investigated the genetic determinants of 54 measures of craniofacial shape and size recorded in ALSPAC (Supplementary Material, Fig. S4) (11). We used these data to test for association between the top SNPs at genome-wide significance and each of the 54 measures of craniofacial development. Because of the large number of correlated craniofacial phenotypes analysed, and consequently the large number of statistical tests performed, we calculated empirical *P*-values for each SNP permuting each genotype against the 54 phenotypes. This procedure is less conservative than a Bonferroni correction (which assumes that the phenotypes are independent) and ensures that the correlation between phenotypes is properly accounted for in the multiple testing correction. Empirical *P*-values were calculated for each SNP and those with *P* < 0.05 were declared significant (Table 2). Using this procedure, we identified three SNPs, which were associated with 10 of the 54 craniofacial measures. Specifically, the SNP rs17101923 (*HMGA2*) was associated with measurements indexing the width of the upper region of the face and nose (Table 2 and Supplementary Material, Fig. S4). Alleles that were associated with increased face width were associated with increased number of teeth and earlier tooth eruption. The rs7924176 marker (*ADK*) was also associated with measures indexing the width of the nose. Alleles that predisposed to earlier tooth eruption were also associated with a wider nose. Furthermore, rs997154 (*AJUBA*) was associated with an increase in height and prominence of the mid-brow.

**Table 2.** Association results for SNPs that met *P* < 0.05 after permutation in the analysis of craniofacial size and shape

Trait^a^	Marker	Gene/locus	Alleles	Frequency of A1	RSQR	Effect	SE	*P*-value	Permuted *P*-value
Width of eye region									
psL-psR: left-to-right palpebrale superius distance	rs17101923	*HMGA2*	**G**/T	0.78	0.9645	0.11	0.028	0.00011	0.0042
piL-piR: left-to-right palpebrale inferius distance	rs17101923	*HMGA2*	**G**/T	0.78	0.9645	0.109	0.028	0.00012	0.0049
exR.yz: distance of the right exocanthion from the *YZ* plane	rs17101923	*HMGA2*	**G**/T	0.78	0.9645	0.099	0.028	0.00043	0.018
enL.yz: distance of the left endocanthion from the *YZ* plane	rs17101923	*HMGA2*	**G**/T	0.78	0.9645	0.096	0.028	0.00067	0.025
enL-enR: left-to-right endocanthion distance	rs17101923	*HMGA2*	**G**/T	0.78	0.9645	0.094	0.028	0.00074	0.028
exL-exR: left-to-right exocanthion distance	rs17101923	*HMGA2*	**G**/T	0.78	0.9645	0.093	0.028	0.00087	0.033
Width of lower part of nose									
prn-alL: pronasale to left alare distance	rs17101923	*HMGA2*	**G**/T	0.78	0.9645	0.08	0.025	0.0013	0.044
sn-alL: subnasale to left alare	rs7924176	*ADK VCL AP3M1*	**A**/G	0.58	0.9714	0.071	0.022	0.0013	0.049
alL-alR: left-to-right alare distance	rs17101923	*HMGA2*	**G**/T	0.78	0.9645	0.078	0.024	0.0014	0.048
Height and prominence of the mid-brow									
g-men: glabella to mid-endocanthion distance	rs997154	*AJUBA*	**G**/A	0.23	0.9698	0.104	0.027	0.00016	0.0069

All alleles are on the forward strand. The effect allele is displayed in bold font and in each case is also the allele associated with increased the number of teeth at 12 months. Freq1 is the allele frequency of the effect allele.

^a^See Supplementary Material Figure S4 for additional information on landmark positions.

We also looked up the top SNP from each of the 15 genome-wide significant loci in a previous analysis of secondary dentition and found that 7 were at least nominally associated (*P* < 0.05) with the number of permanent teeth between 6 and 14 years old (Supplementary Material, Table S4) (7). For the three loci (i.e. *HMGA2*, *BMP4*, *MSRB3*) associated with ‘age at first tooth’ at genome-wide significance, the allele associated with earlier primary tooth eruption was also associated with a greater number of permanent teeth. Furthermore at the four loci (*ADK/VCL/AP3M1*, *2q35*, *CACNB2*, *6q21*) associated with ‘number of primary teeth’, the allele associated with a greater number of teeth at 1 year was also the allele associated with greater number of permanent teeth (6–14 years).

In order to explore the connection between known height-associated SNPs and teeth phenotypes more deeply, we took 180 robustly associated height variants from the Lango Allen *et al*. (12) Giant Consortium meta-analysis and examined the degree to which these SNPs were associated with tooth eruption (Supplementary Material, Table S5). Several height-associated SNPs showed strong evidence of association with tooth eruption in the expected direction (i.e. the height increaser allele was associated with faster tooth eruption/more teeth), including rs1351394 in *HMGA2* (‘Age at first tooth’: *P* = 5.3 × 10^−7^; ‘Number of teeth’: *P* = 1.0 × 10^−9^), rs12534093 in *IGF2BP3* (‘Age at first tooth’: *P* = 0.0026; ‘Number of teeth’: *P* = 2.7 × 10^−5^), rs1490384 near *C6orf173* (‘Age at first tooth’: *P* = 1.0 × 10^−7^; ‘Number of teeth’: *P* = 0.12) and rs1570106 in *RAD51L1* (‘Age at first tooth’: *P* = 0.00012; ‘Number of teeth’: 2.3 × 10^−6^). Overall, however, the number of height-associated SNPs for which the height increaser allele had a positive effect on faster tooth eruption was not greater than expected by chance (‘Age at first tooth’: 89/180 SNPs in the expected direction *P* = 0.94; ‘Number of teeth’: 92/180 SNPs in the expected direction *P* = 0.71). Likewise, a weighted allelic score of height-associated SNPs did not significantly predict the age at first tooth or the number of teeth (‘Age at first tooth’: *P*_META_ = 0.18; ‘Number of teeth’: *P*_META_ = 0.44).

We also regressed height at 17 years in ALSPAC and at 31 years in NFBC1966 on an allelic score constructed from the genome-wide significant SNPs for ‘Age at first tooth’ and ‘Number of teeth’ listed in Table 1. Allelic scores for ‘Age at first tooth’ (*P*_META_ = 0.0012) and ‘Number of teeth’ (*P*_META_ = 9.8 × 10^−4^) showed moderate evidence of association with height; however, the associations appeared to be driven largely by variants in *HMGA2* and *BMP4*. After these SNPS were removed from the construction of the scores, the evidence for association attenuated markedly (‘Age at first tooth’ *P*_META_ = 0.11; ‘Number of teeth’ *P*_META_ = 0.04).

Finally, we conducted a pathway analysis using the ALLIGATOR software (13). In the pathway analyses, an SNP association *P*-value threshold of 0.005 gave the most significant over-representation of genes in pathways in the ‘age at first tooth’ GWAS (see Materials and Methods and Supplementary Material, Table S6a). The top 20 pathways (of the 2276 considered) from this analysis are shown in Table 3; 11 of these pathways had pathway association *P*-values <0.001 and the *P*-value associated with this degree of overrepresentation is 0.004 (Supplementary Material, Table S6A). However, none of the association *P*-value thresholds applied to the ‘number of teeth’ GWAS resulted in a significant over-representation of pathways (Supplementary Material, Table S6B). In Discussion, we focus on the results from the ‘age at first tooth’ GWAS.

**Table 3.** Pathway analysis

Pathway	Number of significant genes in pathway	Total number of genes in pathway	Expected number of genes on list	*P*-value	Study-wide *P*-value	E hits/study
Signalling events mediated by focal adhesion kinase	70	567	47.11	<10^−4^	0.088	0.13
Trail signalling pathway	71	591	47.01	<10^−4^	0.088	0.13
p53 pathway	22	160	9.24	<10^−4^	0.088	0.13
Signalling events mediated by the hedgehog family	13	51	4.78	<10^−4^	0.088	0.13
Class I PI3K signalling events	66	544	43.87	10^−4^	0.1285	0.19
Syndecan-1-mediated signalling events	72	597	49.57	2.0 × 10^−4^	0.17	0.26
Glypican pathway	92	808	68.2	4.0 × 10^−4^	0.2465	0.42
Hedgehog signalling pathway (KEGG)	11	54	3.68	5.0 × 10^−4^	0.2795	0.51
Signalling events mediated by hepatocyte growth factor receptor (c-Met)	71	585	49.13	6.0 × 10^−4^	0.3135	0.59
Class I PI3K signalling events mediated by AKT	54	457	35.99	7.0 × 10^−4^	0.3485	0.66
Glypican 1 network	79	685	57.33	8.0 × 10^−4^	0.3845	0.76
Endothelins	49	364	33.3	0.0011	0.4665	1.03
EGF receptor (ErbB1) signalling pathway	79	697	59.01	0.0019	0.6565	1.8
Internalization of ErbB1	79	697	59.01	0.0019	0.6565	1.8
ErbB1 downstream signalling	79	697	59.01	0.0019	0.6565	1.8
Integrins in angiogenesis	13	63	5.7	0.002	0.671	1.89
Prostate cancer (KEGG)	14	79	6.4	0.002	0.671	1.89
Downstream signalling in naïve CD8+ T cells	9	42	3.87	0.0027	0.758	2.54
1 and 2 methylninaphthalene degradation (KEGG)	3	7	0.31	0.0027	0.758	2.54
Immunoregulatory interactions between A lymphoid and a non-lymphoid cell	6	34	1.59	0.003	0.7875	2.81

Results of top 20 pathways from the ALIGATOR analyses of the ‘age at first tooth’ GWAS. *P*-value threshold of 0.005. There were 1358 genes significant at this threshold from a total of 19 887 genes included in our analysis. All pathways presented are from the NCI pathway interaction database unless stated.

## DISCUSSION

We report genetic variants at 15 loci associated with primary tooth eruption at genome-wide significant levels, including 8 novel variants within or near the following genes: *BMP4*, *CACNB2*, *CDON*, *CALU/OPN1SW*, *AJUBA*, *DLEU7*, *TEX14/RAD51C* and *ADK*. We confirm association with six loci previously associated with primary tooth eruption (*KCNJ2/KCNJ16*, *EDA*, *IGF2BP1*, *MSRB3*, *Chr6q21*, *2q35*) (6). The SNPs from the *ADK* and *2q35* associations have also been previously associated with secondary tooth eruption (7).

Two genes identified in this study that have been implicated repeatedly in animal and human models of tooth development are *BMP4* and *EDA*. *BMP4* is a member of the transforming growth factor beta-1 superfamily of secretory signalling molecules that play essential roles in embryonic development, including mesoderm induction, tooth development, limb formation, bone induction and fracture repair (14). Mutations in *BMP4* can cause eye, brain and digit developmental anomalies (14). *BMP4* is expressed early in tooth development and has an expression profile which coincides with the shift of odontogenic potential from the epithelium to the mesenchyme during development of the tooth bud (15). Recent data suggest that *BMP4* signalling suppresses tooth developmental inhibitors in the tooth mesenchyme, including *Dkk2* and *Osr2*, and synergizes with *Msx1* to activate mesenchymal odontogenic potential for tooth morphogenesis and sequential tooth formation (16). Given *BMP4*'s important role in tooth development, it is perhaps not surprising that SNPs at this locus also associate with timing of tooth eruption. Interestingly, variants within *BMP4* have previously been associated with Parkinson's disease (17) and colorectal cancer in other GWASs (18) suggesting pleiotropic actions of this gene.

*EDA* is a member of the tumour necrosis factor family that signals through a receptor expressed locally in the placodes of all ectodermal appendages as well as in primary and secondary enamel knots (4,19). In humans, mutations in the gene encoding *EDA* can cause hypohidrotic ectodermal dysplasia-1 (20). This syndrome is characterized by a variety of ectodermal abnormalities, including missing teeth and defects in tooth morphology in that crowns of the remaining teeth lack cusps and are conical in shape (20). The ‘Tabby’ mouse (i.e. *EDA* null mutant mouse) represents the murine equivalent of hypohidrotic ectodermal dysplasia-1 (21). These mice often lack incisors and third molars and typically express simplified tooth morphology, including missing or fused cusps (22). Conversely, mice that overexpress *EDA* in the epithelium develop an extra tooth in front of the molars (23). The *EDA* locus was implicated in our previous GWA study of tooth eruption in humans (6). Our results confirm that SNPs at this locus are also associated with subtle effects on tooth development, including alterations in the timing of tooth eruption.

*CACNB2* (rs10740993) is a member of the voltage-gated calcium channel superfamily, and the third ion channel gene to be implicated in tooth eruption (24). Mutations in *CACNB2* have been implicated in a form of Brugada syndrome, a genetic disease characterized by electrocardiogram abnormalities (25). Variants in the gene have also been associated with hypertension, systolic and diastolic blood pressure in GWASs (26). Interestingly, the top SNP from the present study is in LD (*r*^2^ > 0.7) with an SNP associated with blood pressure from the Ehret *et al*. study; the allele associated with earlier tooth eruption is on the same haplotype as the allele associated with lower blood pressure.

Variants in *DLEU7* have also been associated with height in three GWASs (27–29), although the LD between the topmost SNPs from these studies and the topmost SNP from the present study is low (*r*^2^ < 0.01), suggesting that the underlying signals are independent of each other. Transcription factors of *DLEU7* are known to have roles in cell proliferation and differentiation (30).

*CDON* (rs4937076) is involved in muscle cell differentiation and cell adhesion. This gene is part of a cell-surface receptor complex that mediates cell–cell interactions (31). Cell adhesion molecules have been implicated in several processes, including cell migration, growth control and tumour genesis. Cole and Krauss (32) generated mice lacking CDON, 60% of which failed to survive beyond weaning at postpartum day 21. CDON−/− mice displayed the hallmark facial defects associated with microforms of holoprosencephaly, including lack of or solitary central maxillary incisors.

*CALU* (rs1799922) is a calcium-binding protein found in the endoplasmic reticulum. It is involved in protein folding and sorting (33). The gene has no known functions related to tooth eruption, and variants within this gene have not been associated with other phenotypes in GWASs.

*AJUBA* belongs to a group of cell adhesion complexes. It is involved in cell fate determination (34) and is an important regulator of the *WNT* signalling pathway (35). As well as being associated with the number of teeth at 12 months/15 months, the variant rs997154 was also associated with G-men distance (i.e. distance from the glabella to the mid-endocanthion point), suggesting that this gene might be pleiotropically involved in other aspects of craniofacial development besides dentition.

SNPs at three loci (*HMGA2*, *ADK* and *AJUBA*) showed evidence of association with craniofacial distances, particularly those indexing facial width. The SNP rs17101923 is located in an intron of the gene *HMGA2* which is known to contain genetic variants associated with height (29), head circumference (36), intracranial volume (37) and permanent dentition (7). The top variant from our study (rs17101923) is in moderate to high LD with these genetic variants, which could reflect the pleiotropic action on growth, in general, of a single causal variant. Ligon *et al*. (38) report the case of an 8-year-old boy with a *de novo* pericentric inversion of chromosome 12 that truncated the *HMGA2* gene. The patient exhibited multiple clinical features, including premature dentition, enlarged and supernumerary teeth, as well as macrocephaly, flat supraorbital ridges, widely spaced eyes and prominent alveolar ridges. Our results suggest that common SNPs at this locus can also contribute to normal variation in the timing of tooth eruption and craniofacial distances.

We examined the degree to which known height SNPs were associated with tooth eruption, and similarly whether SNPs associated with tooth eruption explained variance in height. Our analyses suggest there exists a subset of known height-associated variants, including those in the *HMGA2*, *IGF2BP3*, *C6orf173* and *RAD51L1* loci that are also associated with tooth eruption. This may be due to these variants exerting a generalized pleiotropic effect on many aspects of growth. For example, SNPs in *HMGA2* have been previously associated with other growth-related phenotypes, including head circumference (36), intracranial volume (37) and birth weight (39) as well as height (12). Likewise, SNPs at the *C6orf173* locus have been associated with age at menarche (40). Despite robust associations of a few, the majority of height-related SNPs were not strongly related to tooth eruption. A weighted allelic score of height-associated variants was not strongly related to tooth eruption and there seemed to be little consistency in the direction of allelic effects for 180 height-associated SNPs across height and tooth eruption phenotypes. Likewise, the majority of genome-wide significant tooth eruption SNPs did not appear to be strongly related to height. The exceptions were SNPs in *HMGA2* and *BMP4*, both which appear to have pleiotropic actions that have been discussed previously. Our results suggest that *BMP4* is likely to contain novel height-associated variants and could also be followed up in this context.

The RAD51 family of genes encode a strand-transfer protein which is thought to be involved in recombinational repair of DNA damage and in meiotic recombination; variants in two of these genes have been highlighted in this study. A variant near *RAD51C* was genome-wide significant for tooth eruption; this gene has been implicated in a Fanconi anaemia-like disorder (41) as well as in rare monogenic forms of breast and ovarian cancer (42), but not in tooth development. Further, a variant in *RAD51L1* reported in Lango Allen *et al*. (12) as being associated with height also showed suggestive evidence of association with primary tooth eruption in this study.

The top 20 pathways identified from the pathway analysis are mostly related to growth and/or cancer. The three genes (*BMP4*, *CDON*, *IGF2BP1*) associated with ‘age at first tooth’ in the GWAS meta-analysis are part of the hedgehog-signalling pathway (*P* = 5 × 10^−4^), signalling events mediated by the Hedgehog family (*P* < 10^−4^) and glypican pathway (*P* = 4 × 10^−4^). Hedgehog signalling has been well described in tooth development (43), along with heparan sulphate proteoglycans (44). Growth factors also play a major role in the interaction between dental epithelium and mesenchyme, as well as cell–cell interactions within these tissues during tooth development (45). Several growth factor receptor signalling pathways for the epidermal growth factor receptor and the hepatocyte growth factor receptor were significant in our analysis [signalling events mediated by the hepatocyte growth factor receptor (c-Met) *P* < 6 × 10^−4^, *EGF* receptor (ErbB1) signalling pathway, internalization of ErbB1, ErbB1 downstream signalling, *P* < 0.0019], which is of interest as their ligands HGF and EGF have been shown to play a role in root development in mice (46,47).

Of the four loci associated with primary tooth development in Pillas *et al*. (6) and confirmed in this study, three have known developmental functions: *KCNJ2*, *EDA* and *IGF2BP1*. The link between normal development and cancer has been noted previously (48), with both involving shifts between cell proliferation and differentiation. Five of the 15 loci identified by our study have been implicated in cancer. As noted earlier, rare mutations in *RAD51C* have been implicated in breast and ovarian cancer. Likewise, a variant in *BMP4* has been found to be associated with colorectal cancer; also a variant in *2q35* has been found to be associated with breast cancer. In both cases, the reported SNP is in high LD with the lead SNP at the respective locus in our study and was also associated with primary tooth eruption. However, whereas the allele associated with increased risk of colorectal cancer was associated with earlier time to tooth eruption, the allele associated with increased risk of breast cancer was associated with fewer teeth at 12 months. Expression of *HMGA2* has been implicated in bladder and lung cancers (49,50). Expression of *IGF II* mRNA-binding protein produced by *IGF2BP1* has been implicated in ovarian cancer (51). Furthermore, the pathway analysis implicated many pathways associated with cancer.

In summary, we have identified eight new loci affecting primary tooth eruption, which together with previously identified loci explain 6.06% of the variation in ‘age of first tooth’ and 4.76% of the variation in ‘number of teeth’. These estimates compare favourably with larger studies on human height; for example, using a total sample size of 39 509, Gudbjartsson *et al*. discovered 27 loci associated with human height, which together explained 3.7% of the variation in human height (27). Several of these variants also appear to exhibit pleiotropic actions, including effects on craniofacial development, height and potentially on disease development in later life. Furthermore, we report a number of genes belonging to pathways involved in growth/development and cancer. A thorough understanding of how the functional variants underlying these associations mediate their effects is likely to yield rich rewards not only in terms of understanding tooth eruption and craniofacial development, but also potentially about how disease develops across the life course.

## MATERIALS AND METHODS

### Participants and phenotypes

Genome-wide association analyses of primary tooth eruption variables were based on data collected from two prospective birth cohorts: ALSPAC and NFBC1966.

#### ALSPAC

ALSPAC is a population-based birth cohort study consisting of 14 541 women and their children recruited in the county of Avon, UK, in the early 1990s (52). Both mothers and children have been extensively followed from the eighth gestational week onwards using a combination of self-reported questionnaires, medical records and physical examinations. Biological samples including DNA have been collected from the participants. Ethical approval was obtained from the ALSPAC Law and Ethics Committee and relevant local ethics committees, and written informed consent provided by all parents. Tooth eruption phenotypes of the children were derived from questionnaires completed by the mothers and included items regarding the ‘age at first tooth’ (assessed at 15months) and the ‘number of teeth’ in the child's mouth (at 15 months).

#### NFBC1966

NFBC1966 followed pregnancies with an expected delivery date in the year 1966 in the Oulu and Lapland provinces of Finland (53). A total of 5403 samples were available for analysis from NFBC1966. In NFBC1966, ‘age at first tooth’ and ‘number of teeth’ were gathered by public health professionals during the children's monthly visits to child welfare centres. ‘Age at first tooth’ was recorded as the month of visit at which the first tooth was observed (so that the first tooth could have erupted at any time between the end of the previous month and the recorded month, i.e. ‘interval censoring’). The number of teeth was recorded at 12 months. All aspects of the study were reviewed and approved by the Ethics Committee of the University of Oulu and by the respective local research committees. Participants gave written informed consent to be included in the study.

### Genotyping

#### ALSPAC

In total, 9912 participants were genotyped using the Illumina HumanHap550 quad genome-wide SNP genotyping platform by 23andMe subcontracting the Wellcome Trust Sanger Institute, Cambridge, UK, and the Laboratory Corporation of America, Burlington, NC, USA. Individuals were excluded from analyses on the basis of excessive or minimal heterozygosity, gender mismatch, individual missingness (>3%), cryptic relatedness as measured by identity by descent (genome-wide IBD >10%) and sample duplication. Individuals were assessed for population stratification using multi-dimensional scaling modelling seeded with HapMap Phase II release 22 reference populations. Individuals of non-European ancestry were removed from further analysis. SNPs with a final call rate of <95%, minor allele frequency (MAF) <1% and evidence of departure from Hardy–Weinberg equilibrium (HWE) (*P* < 5 × 10^−7^) were also excluded from analyses. After data cleaning, 5998 and 6609 individuals had complete phenotype and genotype data for the analysis of ‘age at first tooth’ and ‘number of teeth’, respectively. Individuals were imputed to HapMap Phase II (Build 36, release 22) using the Markov Chain Haplotyping software (MACH v.1.0.16) (32). X chromosome imputation was carried out on the non-pseudo autosomal region of the X chromosome only, using CEU individuals from HapMap Phase III (release 2). Only SNPs exceeding an rsq imputation quality metric of 0.3 and an MAF of >1% were included in subsequent analyses.

#### NFBC1966

The Illumina HumanCNV370-Duo DNA Analysis BeadChip was used for genotyping NFBC1966. SNPs were excluded from the analysis if the call rate in the final sample was <95%, if there was a lack of HWE (*P* < 5 × 10^−4^) or if the MAF was <1%; more details of genotyping and quality control procedures can be found in Sabatti *et al*. (53). After quality control, 328 077 SNPs were available for imputation. Imputation was carried out using IMPUTEv1 with CEU haplotypes from HapMap Phase II (release 21) as the reference panel. X chromosome imputation was carried out in the non-pseudo-autosomal region of the X chromosome (54). Only SNPs exceeding an ‘info’ metric of 0.3 and an MAF of >1% were included in subsequent analyses. After data cleaning, 5120 and 4904 individuals had complete phenotype and genotype data for the analysis of ‘age at first tooth’ and ‘number of teeth’, respectively.

### Statistical analysis

In order to account for censoring of the data, the association between expected allelic dosage and ‘age at first tooth’ was analysed using parametric survival analysis, with the Gaussian distribution used to model event time. The ALSPAC data were modelled as ‘right censored’, whereas the data in NFBC1966 were modelled as ‘interval censored’. The association between expected allelic dosage and the number of teeth was analysed using proportional odds logistic regression (ordinal regression). Teeth are known to erupt in pairs; hence Poisson regression (which assumes that the events of interest are independent) was not appropriate. Analyses were adjusted for sex (ALSPAC and NFBC1966), gestational age (ALSPAC and NFBC1966) and age of completion (ALSPAC only, all NFBC1966 measurements were recorded at 12 months). In addition, in NFBC1966, the top 10 ancestry-derived principal components were tested for association with the phenotypes and were included in the GWAS of that phenotype if they associated at *P* < 0.05. This resulted in the inclusion of the second principal component in the NFBC1966 analysis of tooth eruption, and no principal components in the analysis of ‘number of teeth’. Data were analysed using the R software package 2.9.1.

Results from both studies were combined using a fixed effects inverse variance meta-analysis, using the software package METAL (55). This approach weights effect size estimates according to the inverse of their standard errors. Variance explained by each SNP was calculated as 1 − var(res.full)/var(res.null)*100 of the model (proportional odds logistic regression/survival regression) with age at measurement, sex and gestational age (var, variance; res, residuals). To correct for over-fitting each individuals, phenotype was estimated from a model that did not include that individual (6).

In order to investigate the possibility of secondary signals at loci that met the criteria for genome-wide significance (defined as *P* < 5 × 10^−8^), conditional regression analyses were performed conditioning on the most strongly associated SNP in each region. We then applied the Nyholt method for multiple testing correction to derive a threshold for determining statistical significance based on the number of SNPs tested and taking into account LD across the region (10). These regions were defined based on locations of nearby recombination hot spots. In absence of these, we defined a region as ±250 kb from the top SNP.

We then investigated whether any of our genome-wide significant loci exerted pleiotropic actions by looking at their association with height, craniofacial shape and size and permanent tooth eruption. In the case of height, we conducted linear regression of height measured at age 17 (in ALSPAC) and age 31 (in NFBC) on genome-wide significant SNPs from Table 1. For craniofacial shape and size, we looked up genome-wide significant SNPs from the present study in the results from a previous GWAS of the 54 variables characterizing different facial features consisting of facial height, width, convexity and prominence of landmark in respect to facial planes (11) (see Supplementary Material, Fig. S4, for a list of distances examined). To account for multiple testing, empirical levels of significance were determined using permutation analyses, where for each SNP, genotype was permuted with respect to the 54 craniofacial variables. In this way, an adjusted *P*-value could be calculated for each SNP, which took into account the fact that association had been tested across the 54 correlated variables.

Analyses involving secondary tooth eruption were performed using data from the Danish National Birth Cohort (7). The genotype data were derived from two on-going GWASs of preterm birth (56) and obesity (57). The study combined all observations between age 6 and 14 years (starting with the 6th and stopping with the 14th birthday), the time period when eruption of permanent dentition usually occurs. For each visit to the dentist, the total number of permanent teeth (excluding third molars) was recorded, and regressed on age. The resulting residuals were then standardized, and for each individual the mean residual across all available records was used as the phenotype. Genotypes for the two GWASs were imputed separately using MACH (54). The resulting imputed genotypes were analysed separately and meta-analysed with METAL (55).

Pathway analyses of the ‘age at first tooth’ and ‘number of teeth’ GWASs were performed using the ALIGATOR method (13). The implementation of ALIGATOR described in Holmans *et al*. (13) maps genes to gene ontology categories; however, the method is equally applicable to other gene to pathway mappings and we used ALIGATOR to test for the enrichment of significant genes within biological pathways; significant genes are defined by the method as those with one or more SNPs with an association *P*-value less than a predefined threshold within the gene. We considered 2276 pathways curated by the Broad Institute (http://www.broadinstitute.org/gsea/), as well as pathways from ‘Pathway Commons’ (http://www.pathwaycommons.org/pc/home.do) and additional inflammatory pathways (58,59). All genotyped and imputed SNPs with minor allele frequencies of >0.05 were included in the analyses. The method corrects for variable gene size, and multiple testing of non-independent pathways using permutation. All ALIGATOR analyses used 10 000 simulated replicate gene lists and 2000 simulated replicate studies. We compared results using *P*-value thresholds for association at 0.005, 0.001 and 0.0005 and 0.0001, and as suggested Holmans *et al*. (13) report results from the analysis showing the most significant enrichment of pathways.

## FUNDING

This work and D.M.E. were supported by a Medical Research Council New Investigator Award (MRC G0800582). G.F. is funded by a Wellcome Trust 4-year PhD studentship in molecular, genetic and life course epidemiology (WT083431MA). The UK Medical Research Council (grant 74882), the Wellcome Trust (grant 076467) and the University of Bristol provide core support ALSPAC. G.F., L.P., J.P.K., N.J.T., G.D.S. and D.M.E. work in a centre that receives funds from the UK Medical Research Council (G0600705) and the University of Bristol. C.J.H. and V.J.W. are funded by European Union's Seventh Framework Programme under EC-GA no. 279185 (EUCLIDS). The research of I.P. is funded through the European Community's Seventh Framework Programme (FP7/2007-2013), ENGAGE project, grant agreement HEALTH-F4-2007-201413. Northern Finland Birth Cohort 1966 (NFBC1966) received financial support from the Academy of Finland (project grants 104781, 120315, 129269, 1114194, 139900/24300796, Center of Excellence in Complex Disease Genetics and SALVE), University Hospital Oulu, Biocenter, University of Oulu, Finland (75617), the European Commission (EURO-BLCS, Framework 5 award QLG1-CT-2000-01643), NHLBI grant 5R01HL087679-02 through the STAMPEED programme (1RL1MH083268-01), NIH/NIMH (5R01MH63706:02), ENGAGE project and grant agreement HEALTH-F4-2007-201413, the Medical Research Council, UK (G0500539, G0600705, G0600331, PrevMetSyn/SALVE, PS0476) and the Wellcome Trust (project grant GR069224, WT089549), UK. Replication genotyping was supported in part by MRC grant G0601261, Wellcome Trust grants 085301, 090532 and 083270, and Diabetes UK grants RD08/0003704 and BDA 08/0003775. GOYA is a nested study within The Danish National Birth Cohort, which was established with major funding from the Danish National Research Foundation. Additional support for this cohort has been obtained from the Pharmacy Foundation, the Egmont Foundation, The March of Dimes Birth Defects Foundation, the Augustinus Foundation and the Health Foundation. Funding to pay the Open Access publication charges for this article was provided by the Wellcome Trust.

## Supplementary Material

Supplementary Data
