# Supplementary material for: Genome-wide association study of primary tooth eruption identifies pleiotropic loci associated with height and craniofacial distances
Source: Hum Mol Genet. 2013 May 23;22(18):3807–17. doi: 10.1093/hmg/ddt231 (PMC3749866; doi:10.1093/hmg/ddt231)
Supplement: Supplementary Data [file supp_22_18_3807__index.html]

Genome-wide association study of primary tooth eruption identifies pleiotropic loci associated with height and craniofacial distances — Supplementary Data 

# Genome-wide association study of primary tooth eruption identifies pleiotropic loci associated with height and craniofacial distances

## 

Supplementary Data

**Files in this Data Supplement:**

- Supplementary Data - Pdf file
