# Supplementary material for: Genome-wide association study of primary tooth eruption identifies pleiotropic loci associated with height and craniofacial distances
Source: Hum Mol Genet. 2013 May 23;22(18):3807–17. doi: 10.1093/hmg/ddt231 (PMC3749866; doi:10.1093/hmg/ddt231)
Supplement: Supplementary Data [file supp_ddt231_ddt231supp.pdf]

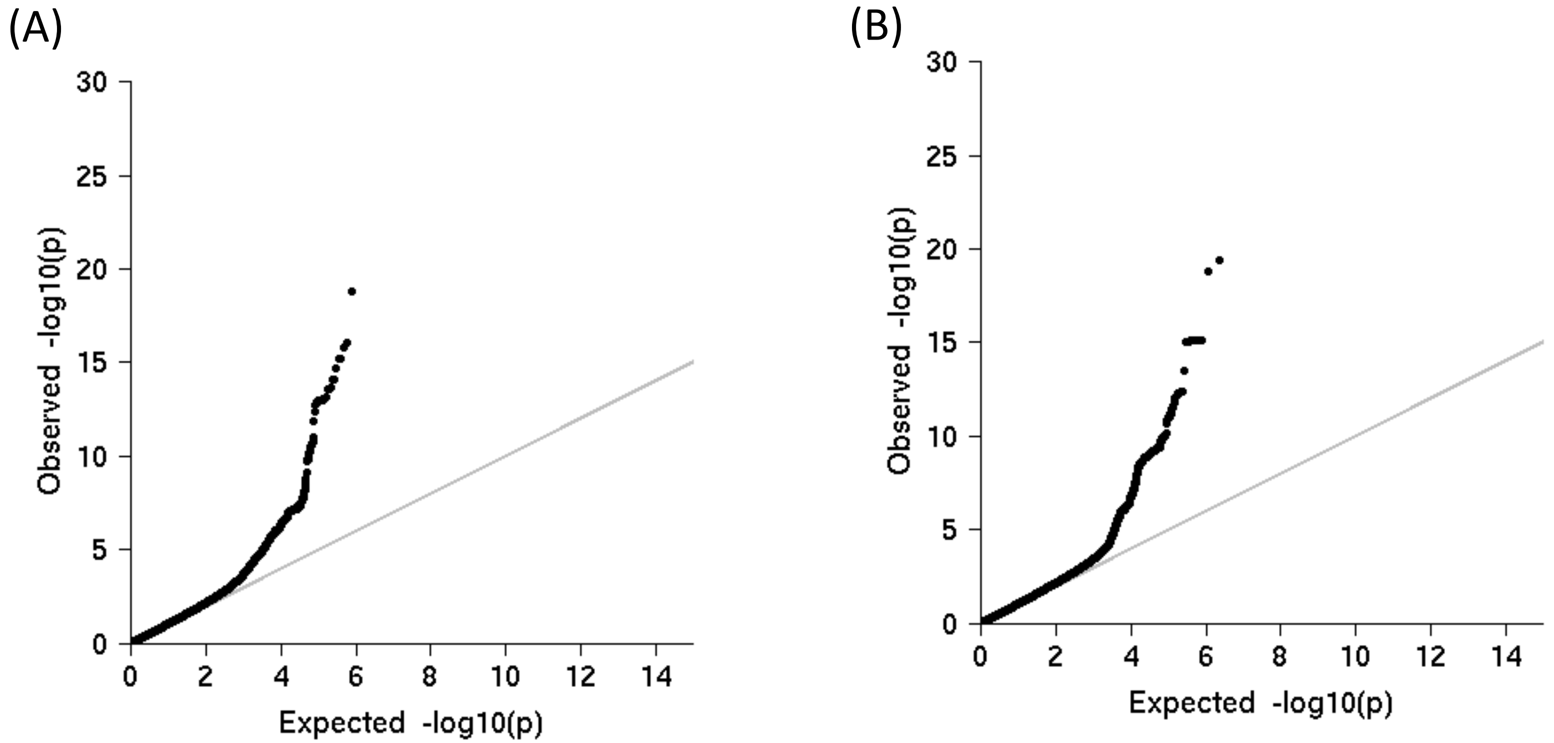


**Supplementary Figure 1**: QQ plot for the combined meta-analysis of (a) ‘Age at first tooth’ and (b) ‘number of teeth’ respectively. Both QQ plots show substantial deviation from the line of expectation for low p values only, suggesting the existence of common genetic variants influencing primary tooth eruption, but not substantial population stratification. The genomic inflation factors were $\lambda = 1.07$ for ‘age at first tooth’, and $\lambda = 1.06$ for ‘number of teeth’.

(A)

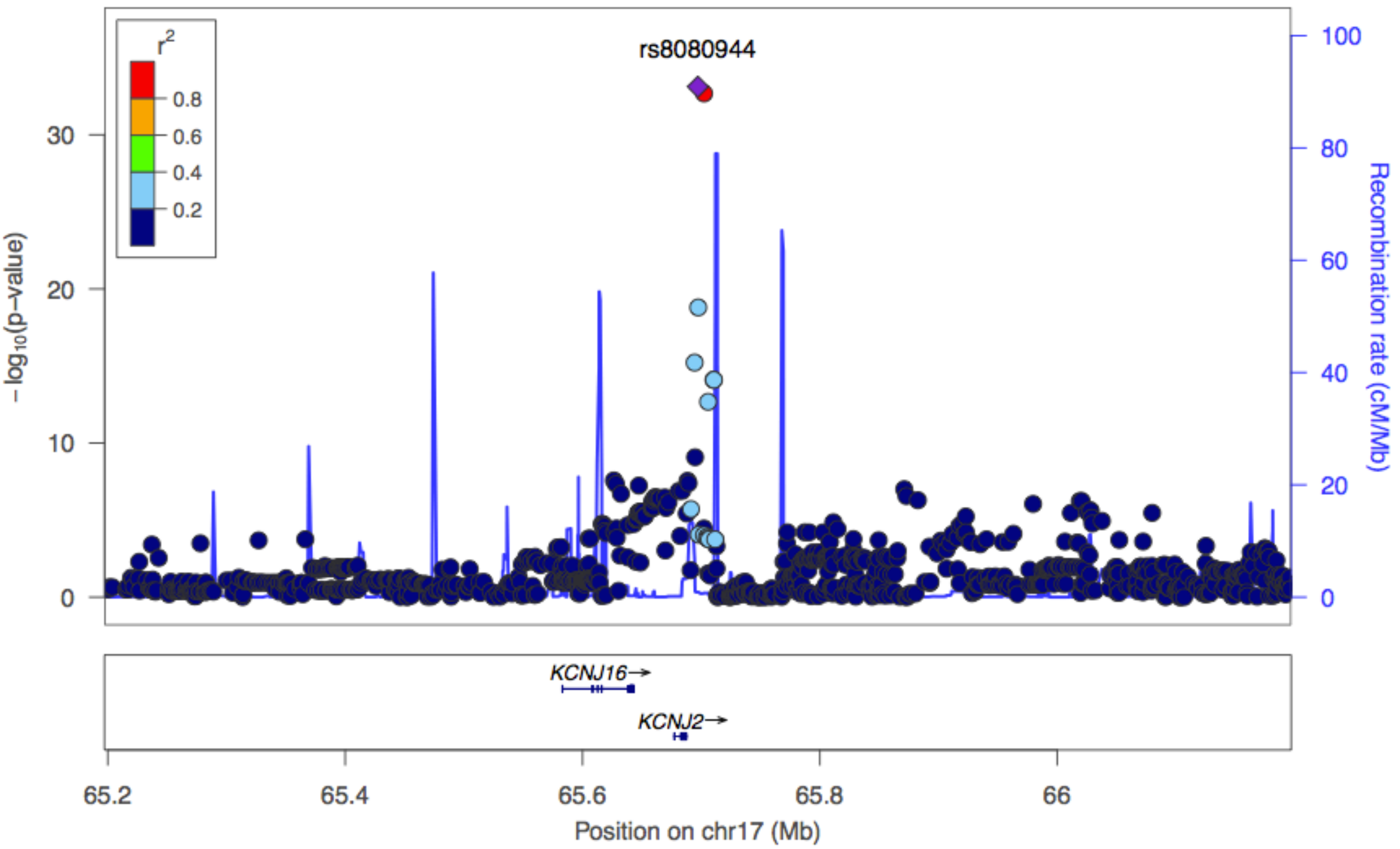

r2
0.8
0.6
0.4
0.2
rs8080944
30
20
10
0
−log10(p−value)
100
80
60
40
20
0
Recombination rate (cM/Mb)
KCNJ16→
KCNJ2→
65.2
65.4
65.6
65.8
66
Position on chr17 (Mb)

(B)

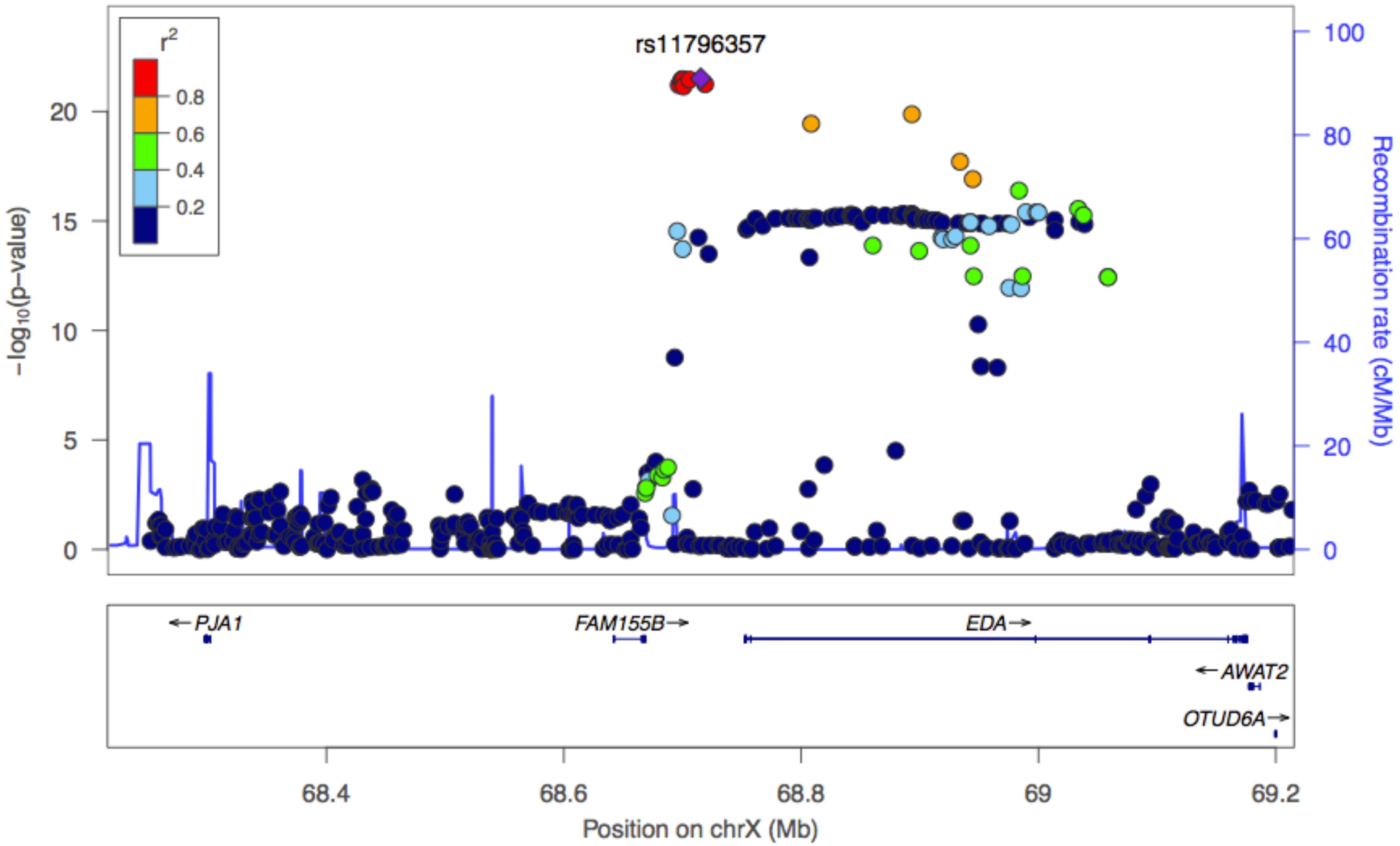

r2
0.8
0.6
0.4
0.2
rs11796357
-log10(p-value)
Recombination rate (cM/Mb)
0
5
10
15
20
100
80
60
40
20
0
PJA1
FAM155B
EDA
AWAT2
OTUD6A
68.4
68.6
68.8
69
69.2
Position on chrX (Mb)

(C)

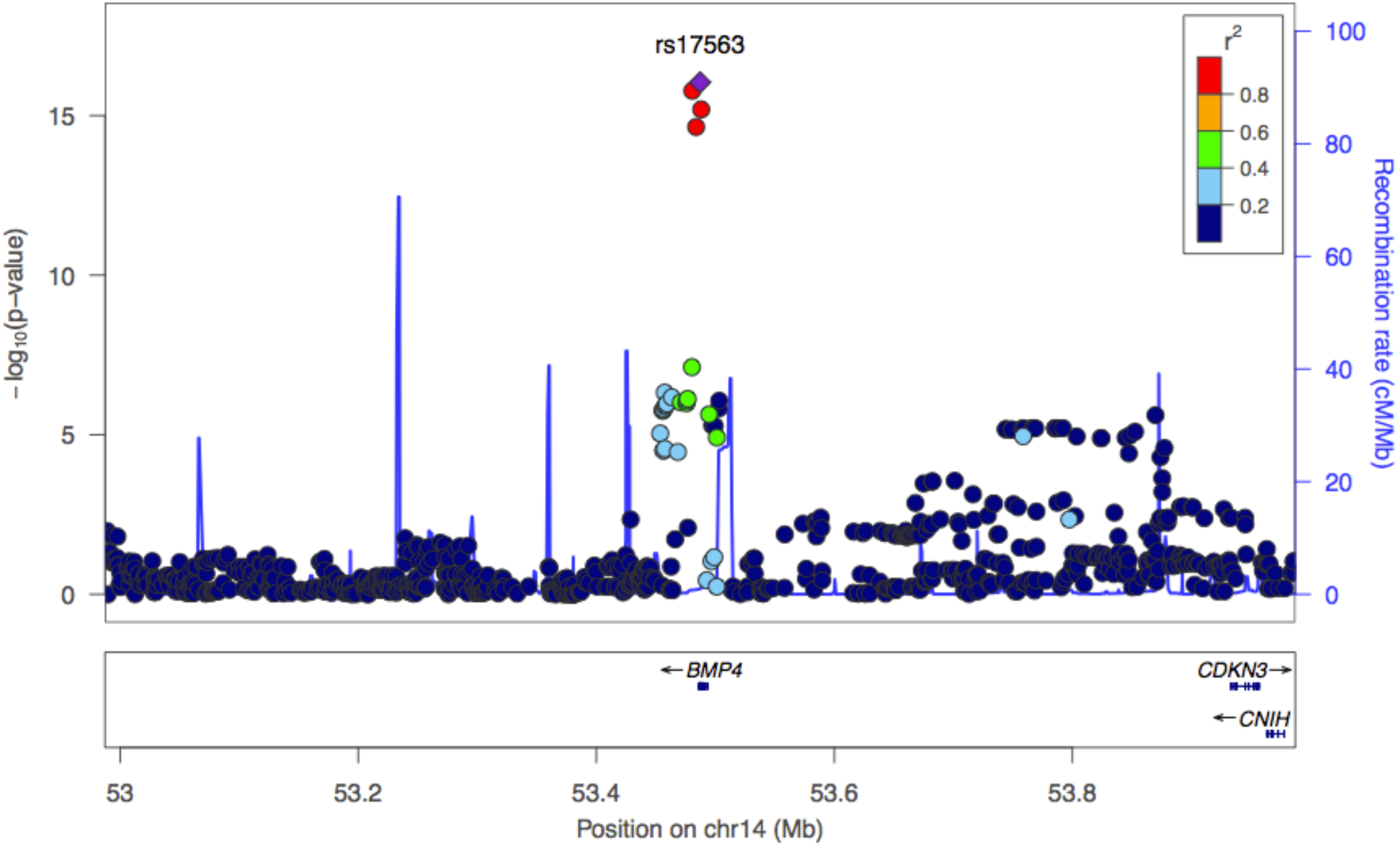

rs17563
r²
0.8
0.6
0.4
0.2
15
10
5
0
− log₁₀(p−value)
100
80
60
40
20
0
Recombination rate (cM/Mb)
← BMP4
CDKN3 →
← CNIH
53
53.2
53.4
53.6
53.8
Position on chr14 (Mb)

(D)

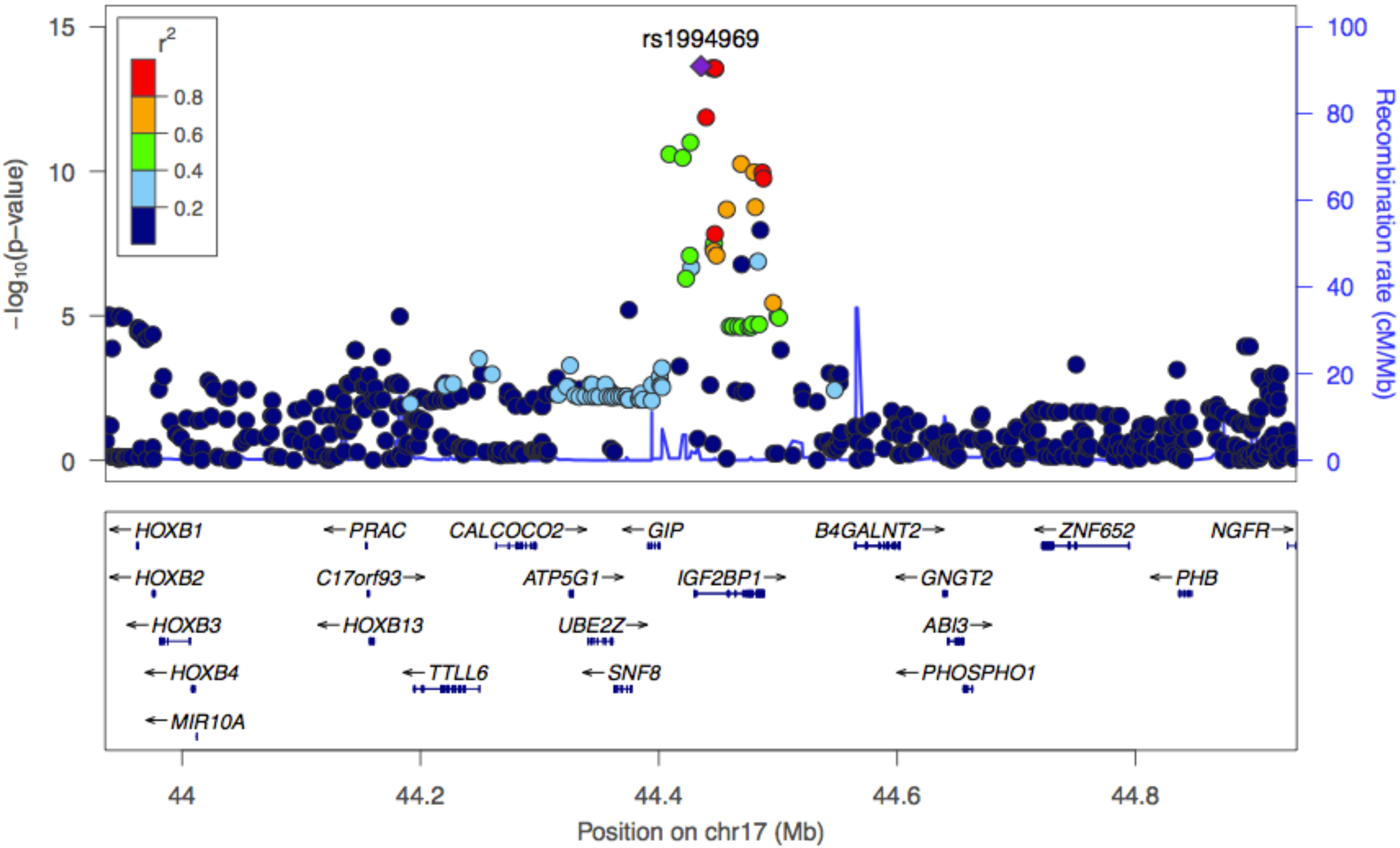

rs1994969
r2
0.8
0.6
0.4
0.2
15
10
5
0
−log10(p−value)
100
80
60
40
20
0
Recombination rate (cM/Mb)
HOXB1
HOXB2
HOXB3
HOXB4
MIR10A
PRAC
C17orf93
HOXB13
TTLL6
CALCOCO2
ATP5G1
UBE2Z
SNF8
GIP
IGF2BP1
B4GALNT2
GNGT2
ABI3
PHOSPHO1
ZNF652
NGFR
PHB
44
44.2
44.4
44.6
44.8
Position on chr17 (Mb)

(E)

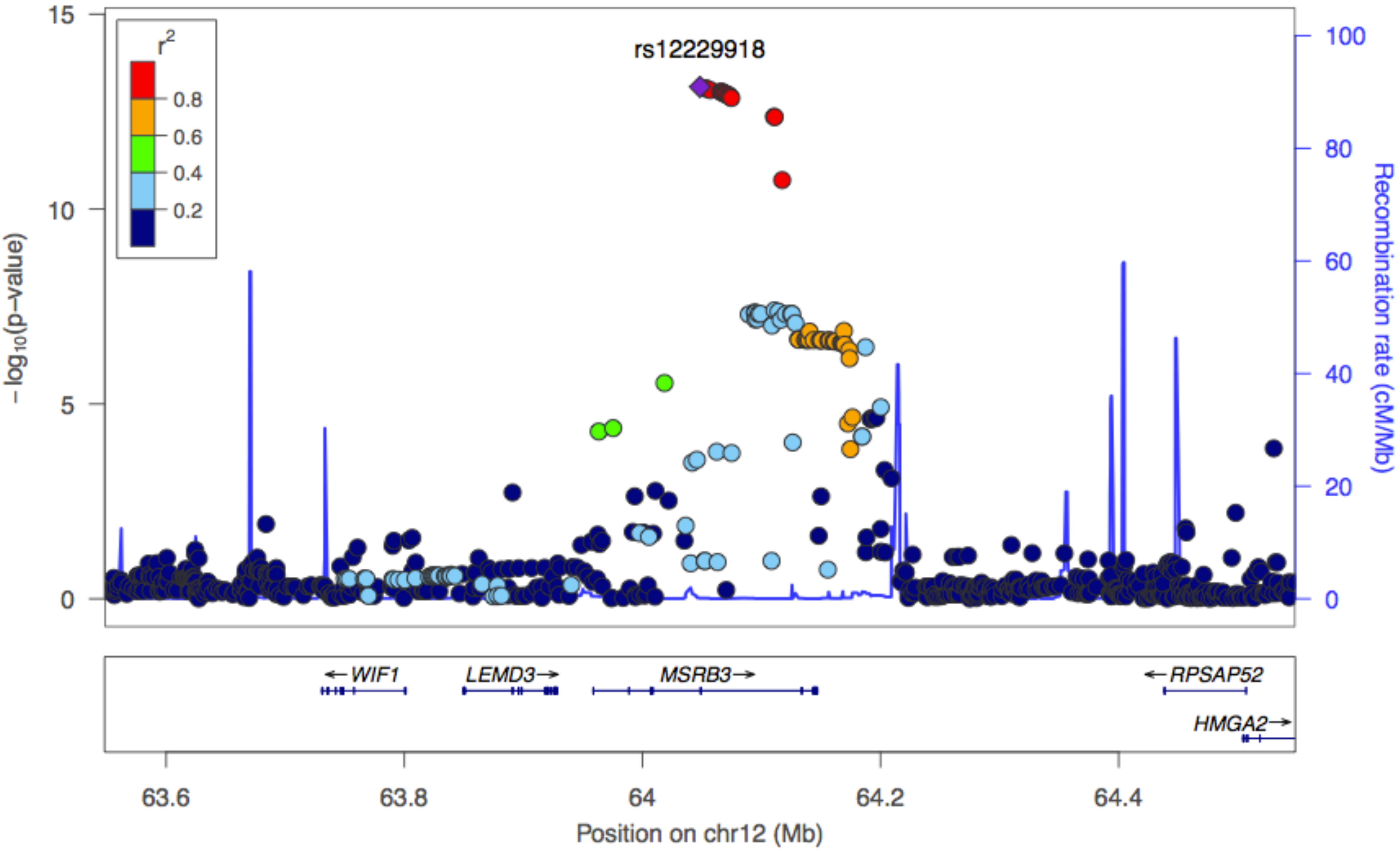
rs12229918
r²
0.8
0.6
0.4
0.2
15
10
5
0
− log10(p−value)
100
80
60
40
20
0
Recombination rate (cM/Mb)
←WIF1
LEMD3→
MSRB3→
←RPSAP52
HMGA2→
63.6
63.8
64
64.2
64.4
Position on chr12 (Mb)

(F)

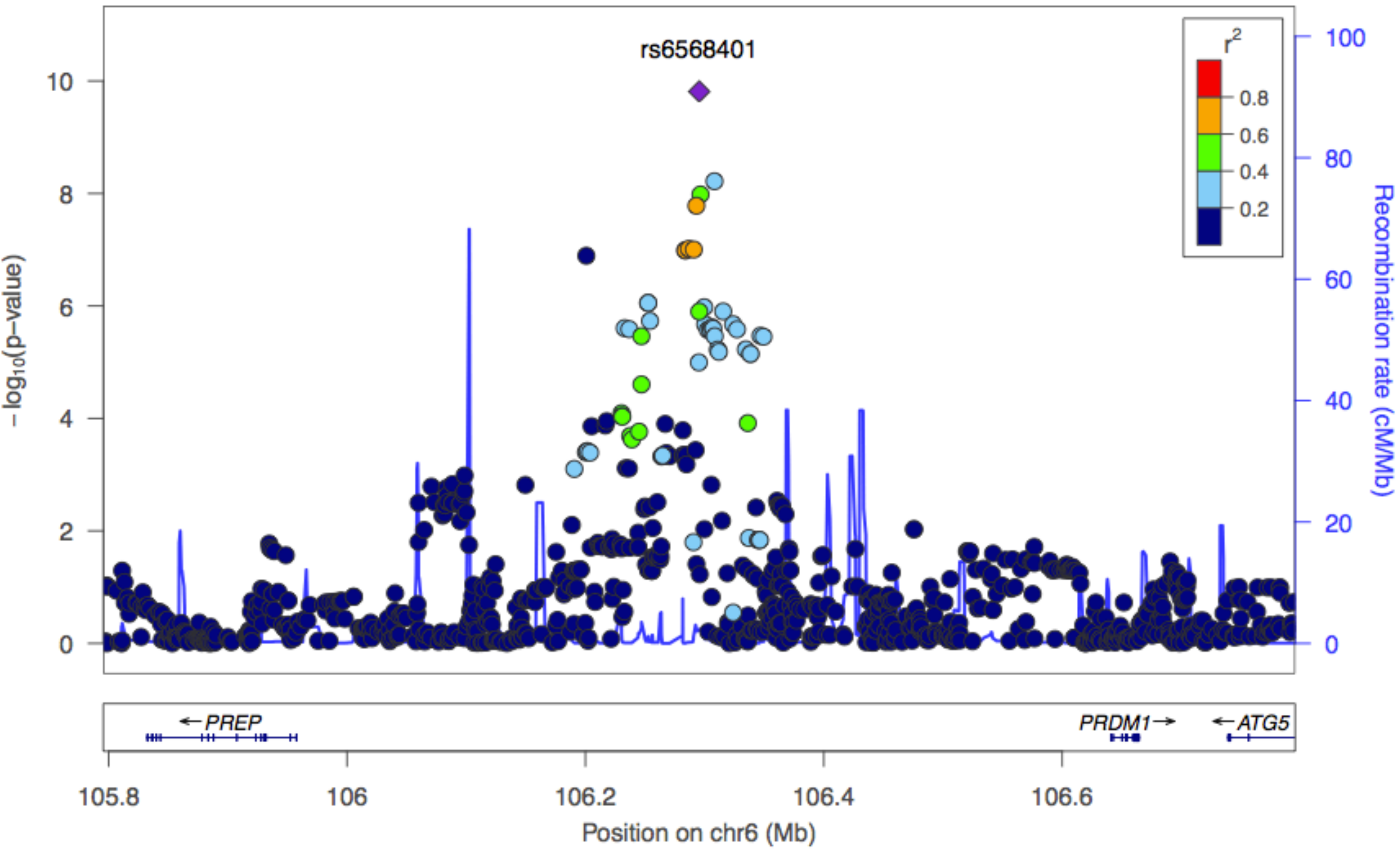
rs6568401
r²
0.8
0.6
0.4
0.2
10
8
6
4
2
0
− log10(p−value)
100
80
60
40
20
0
Recombination rate (cM/Mb)
← PREP
PRDM1 →
← ATG5
105.8
106
106.2
106.4
106.6
Position on chr6 (Mb)

(G)

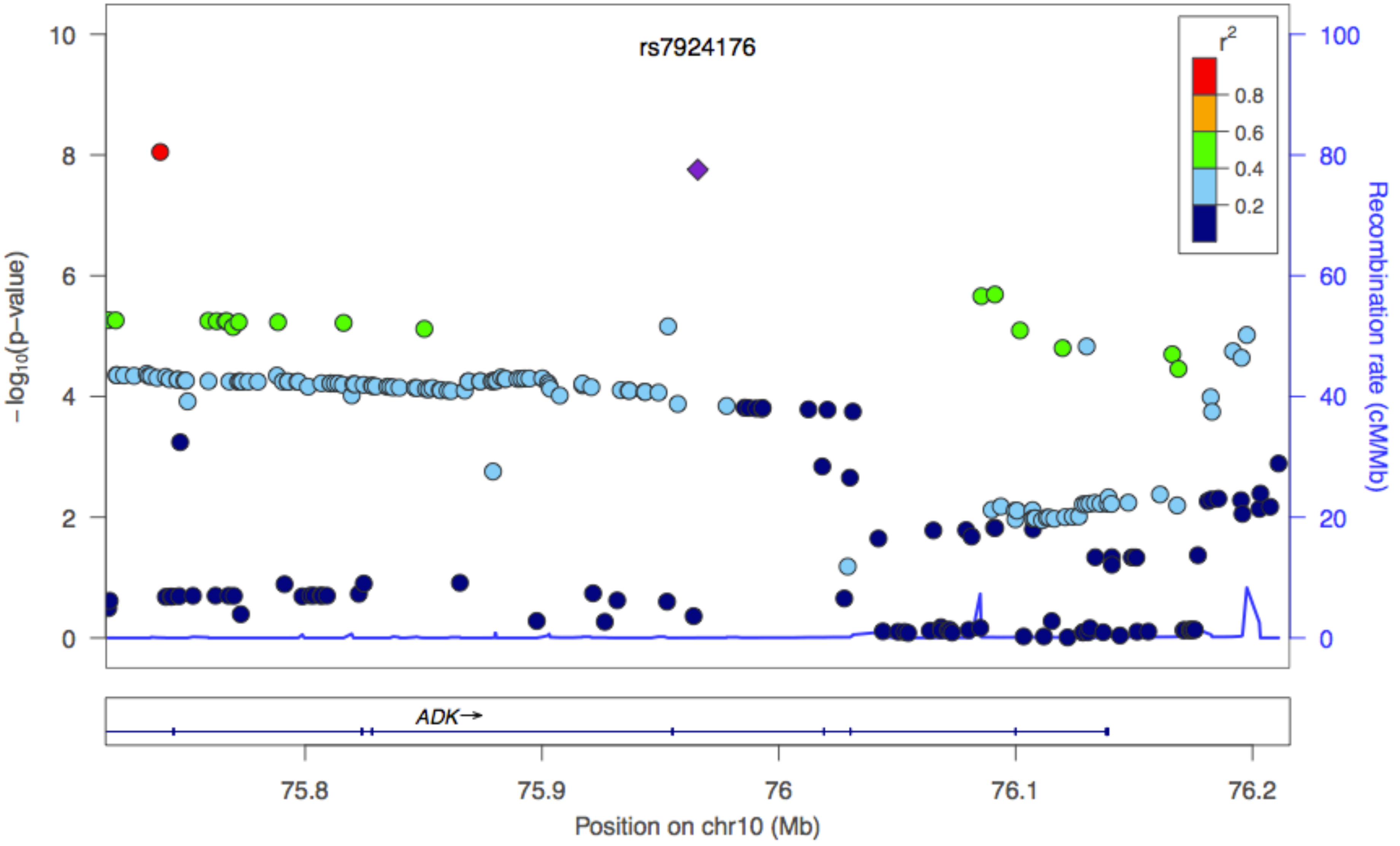
rs7924176
r2
0.8
0.6
0.4
0.2
10
8
6
4
2
0
−log10(p−value)
100
80
60
40
20
0
Recombination rate (cM/Mb)
ADK→
75.8
75.9
76
76.1
76.2
Position on chr10 (Mb)

(H)

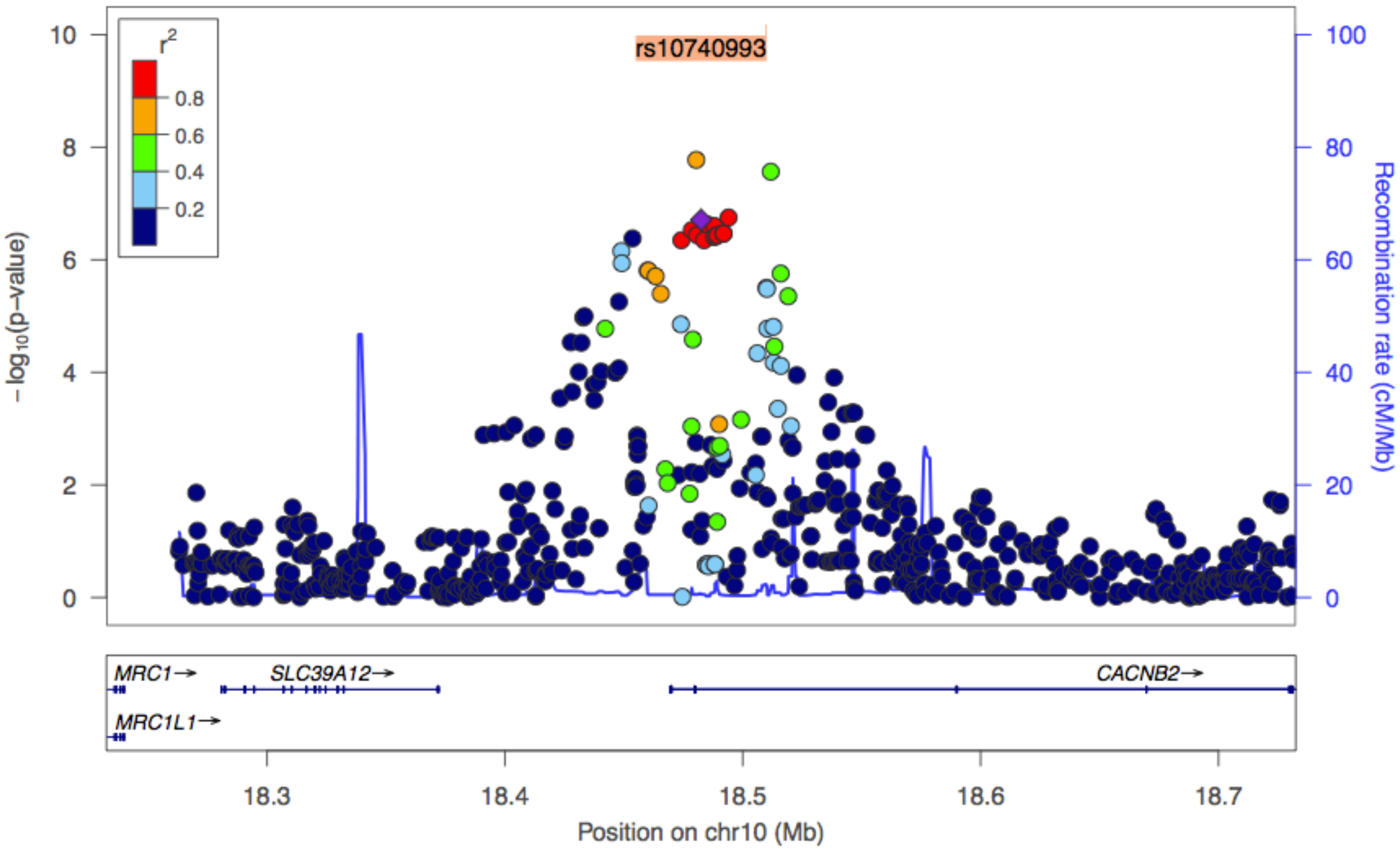

rs10740993
r²
0.8
0.6
0.4
0.2
10
8
6
4
2
0
100
80
60
40
20
0
−log10(p−value)
Recombination rate (cM/Mb)
MRC1→
SLC39A12→
CACNB2→
MRC1L1→
18.3
18.4
18.5
18.6
18.7
Position on chr10 (Mb)

(I)

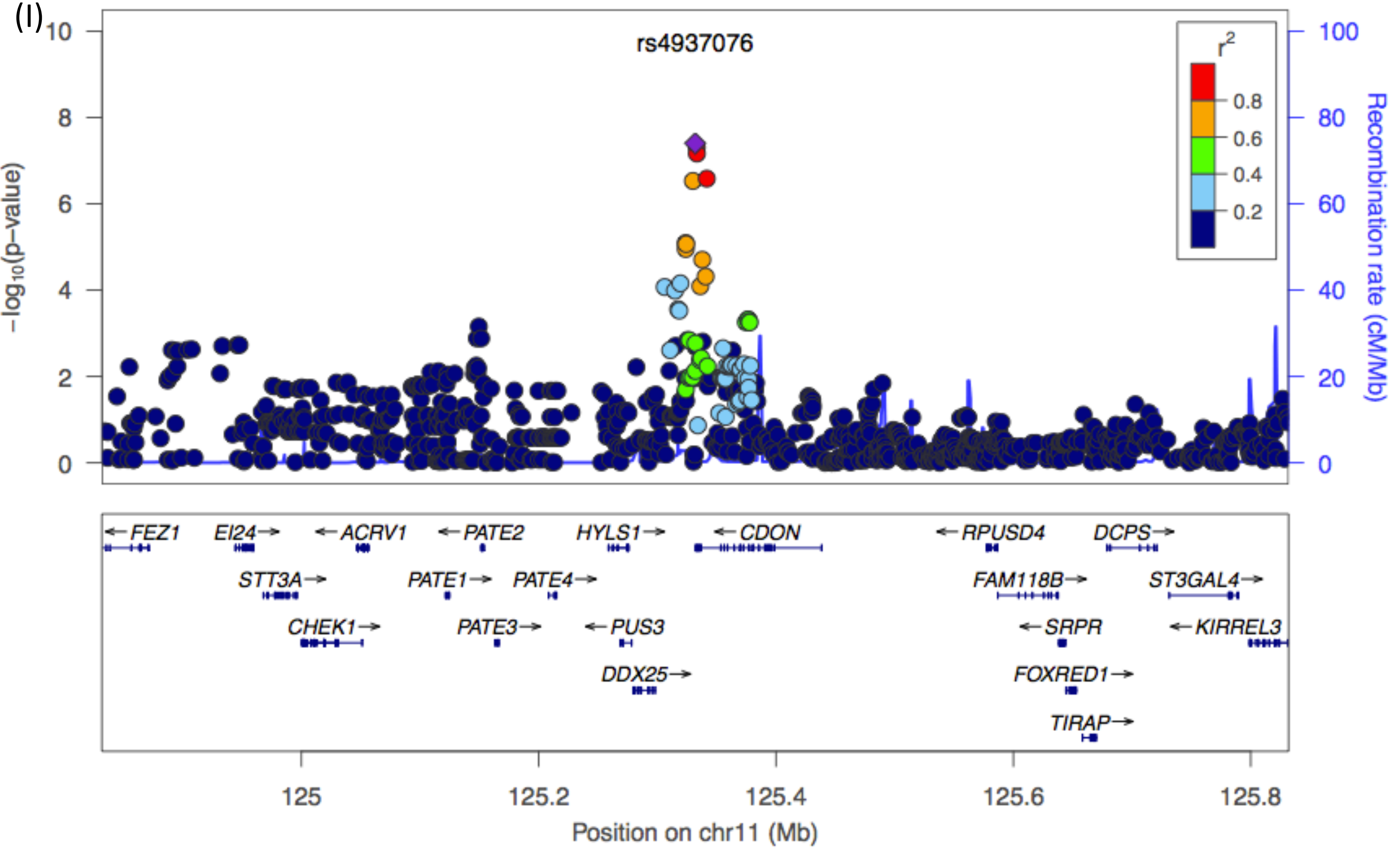

rs4937076
r2
0.8
0.6
0.4
0.2
−log10(p−value)
Recombination rate (cM/Mb)
0
2
4
6
8
10
20
40
60
80
100
FEZ1
EI24
ACRV1
PATE2
HYLS1
CDON
RPUSD4
DCPS
STT3A
PATE1
PATE4
FAM118B
ST3GAL4
CHEK1
PATE3
PUS3
SRPR
KIRREL3
DDX25
FOXRED1
TIRAP
125
125.2
125.4
125.6
125.8
Position on chr11 (Mb)

(J)

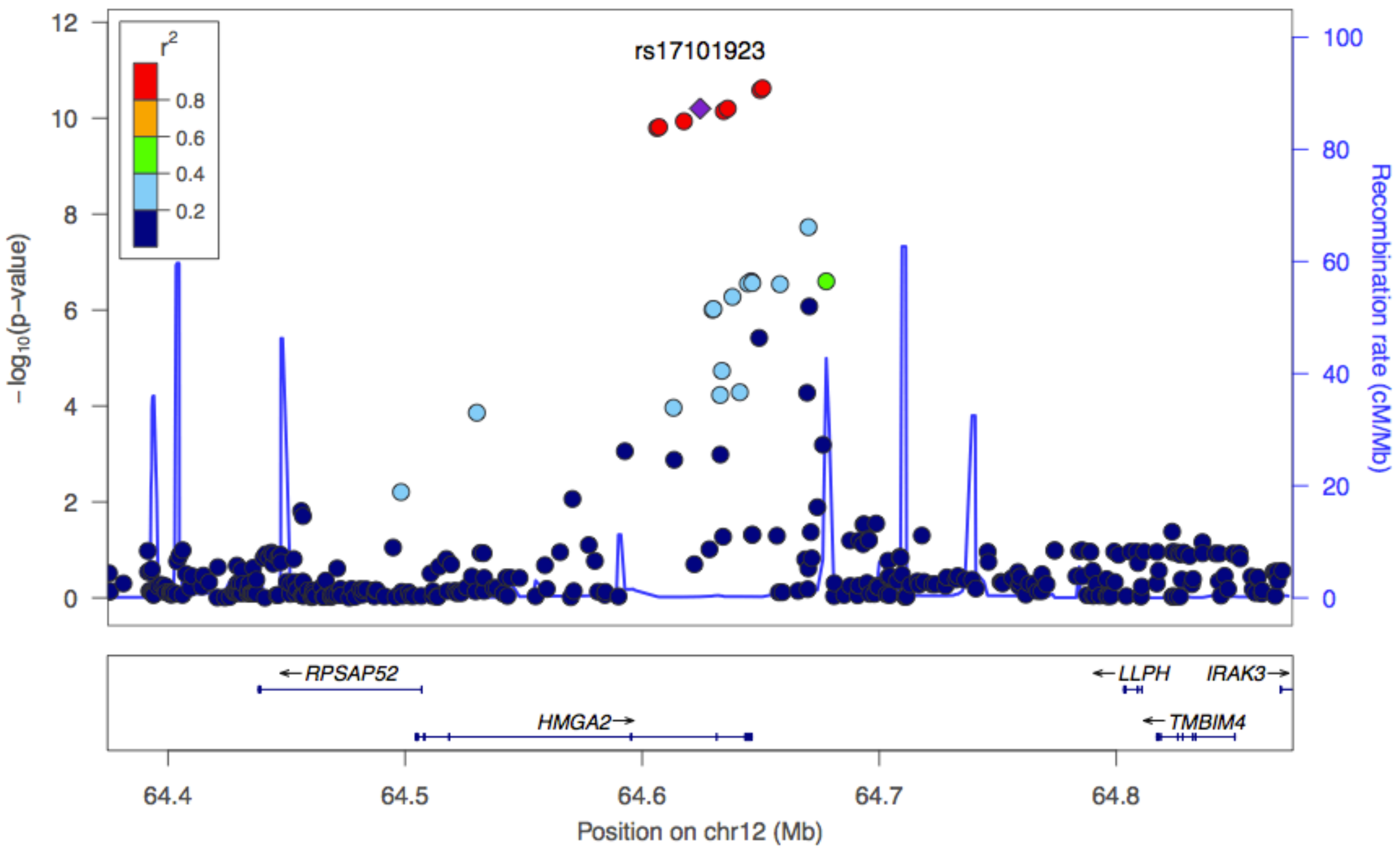

rs17101923
r2
0.8
0.6
0.4
0.2
12
10
8
6
4
2
0
− log10(p−value)
100
80
60
40
20
0
Recombination rate (cM/Mb)
← RPSAP52
HMGA2 →
← LLPH
IRAK3 →
← TMBIM4
64.4
64.5
64.6
64.7
64.8
Position on chr12 (Mb)

(K)

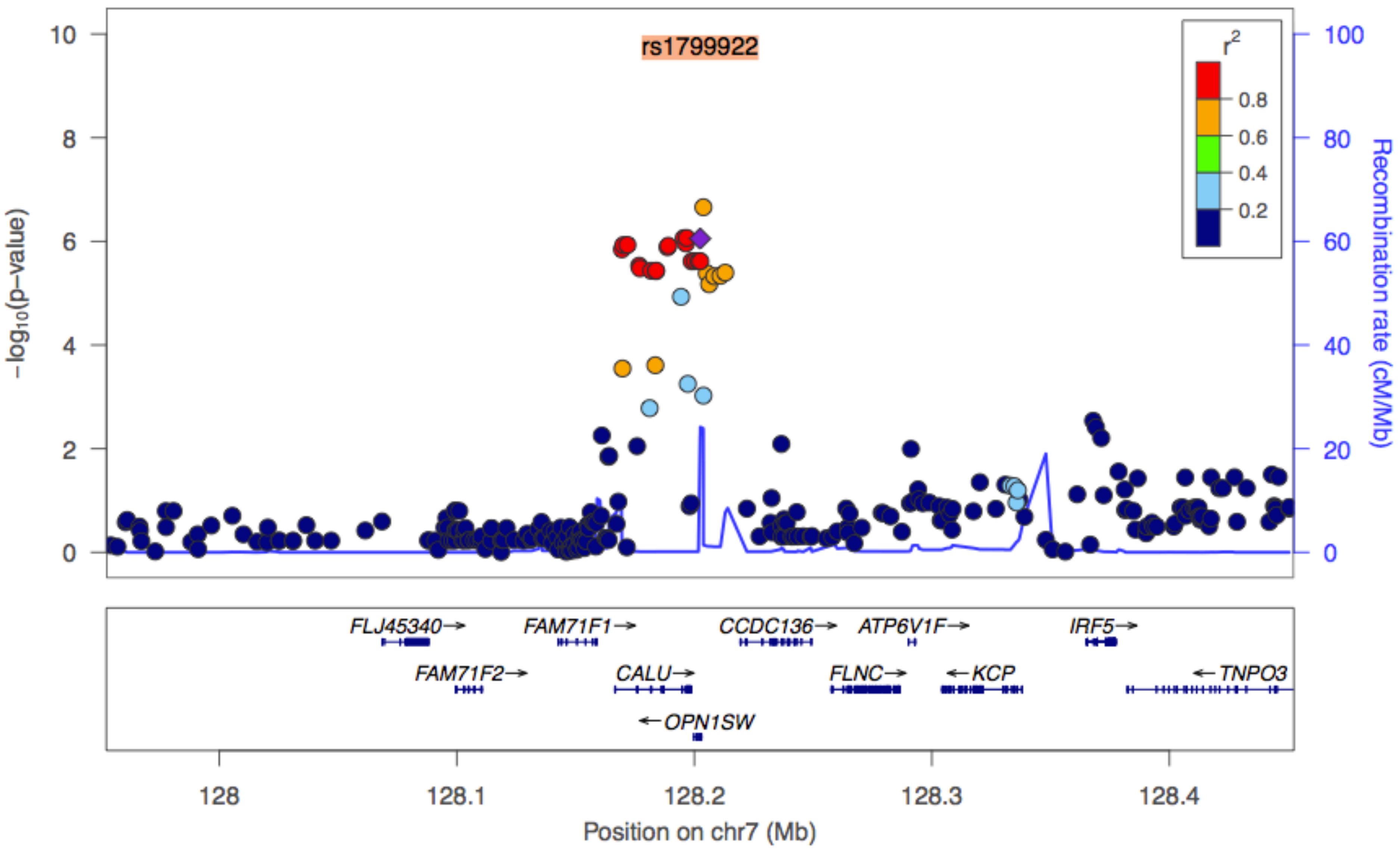
rs1799922
r2
0.8
0.6
0.4
0.2
−log10(p−value)
Recombination rate (cM/Mb)
FLJ45340→
FAM71F1→
CCDC136→
ATP6V1F→
IRF5→
FAM71F2→
CALU→
FLNC→
←KCP
←TNPO3
←OPN1SW
128
128.1
128.2
128.3
128.4
Position on chr7 (Mb)

(L)

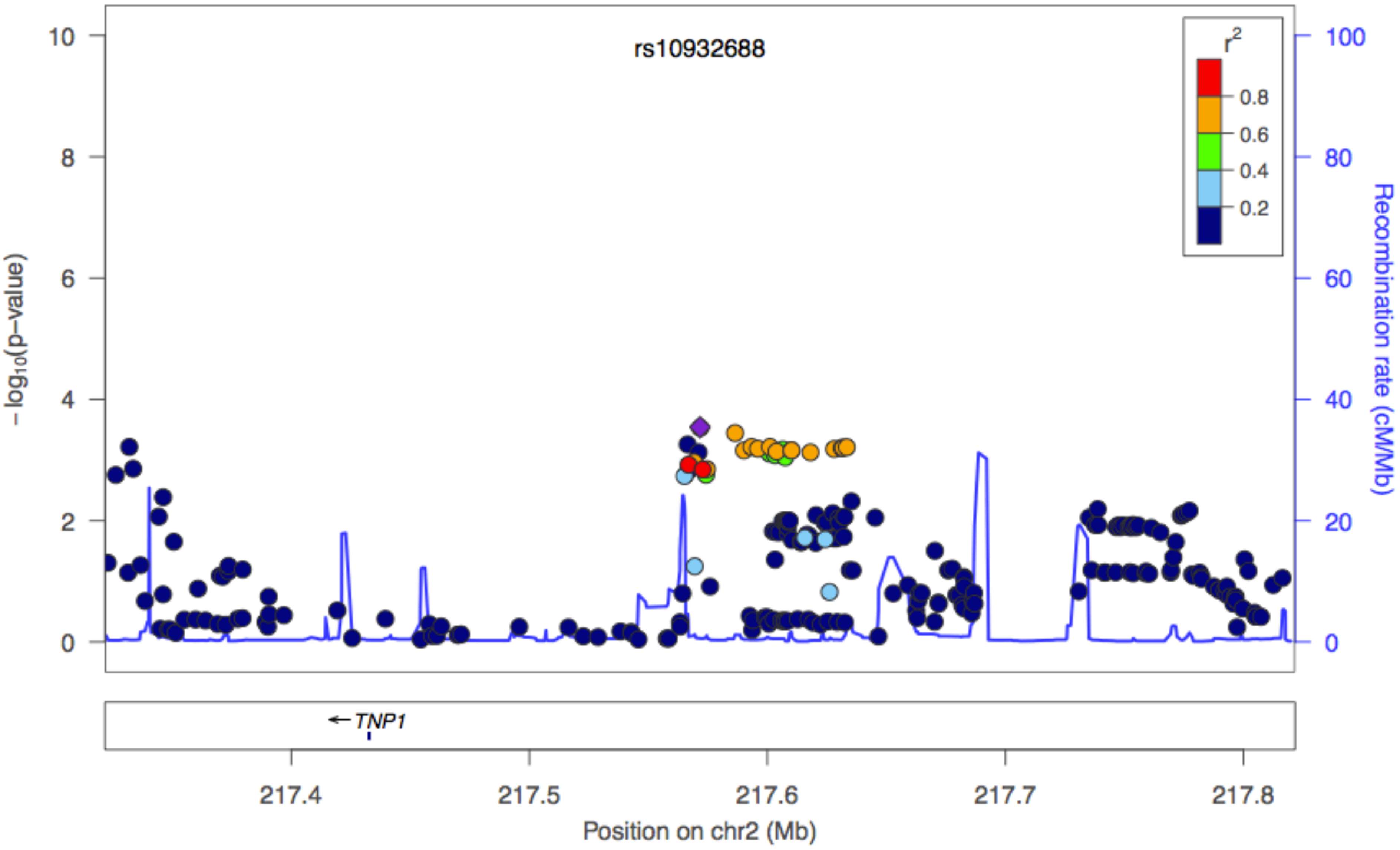
rs10932688
r2
0.8
0.6
0.4
0.2
10
8
6
4
2
0
100
80
60
40
20
0
−log10(p−value)
Recombination rate (cM/Mb)
← TNP1
217.4
217.5
217.6
217.7
217.8
Position on chr2 (Mb)

(M)

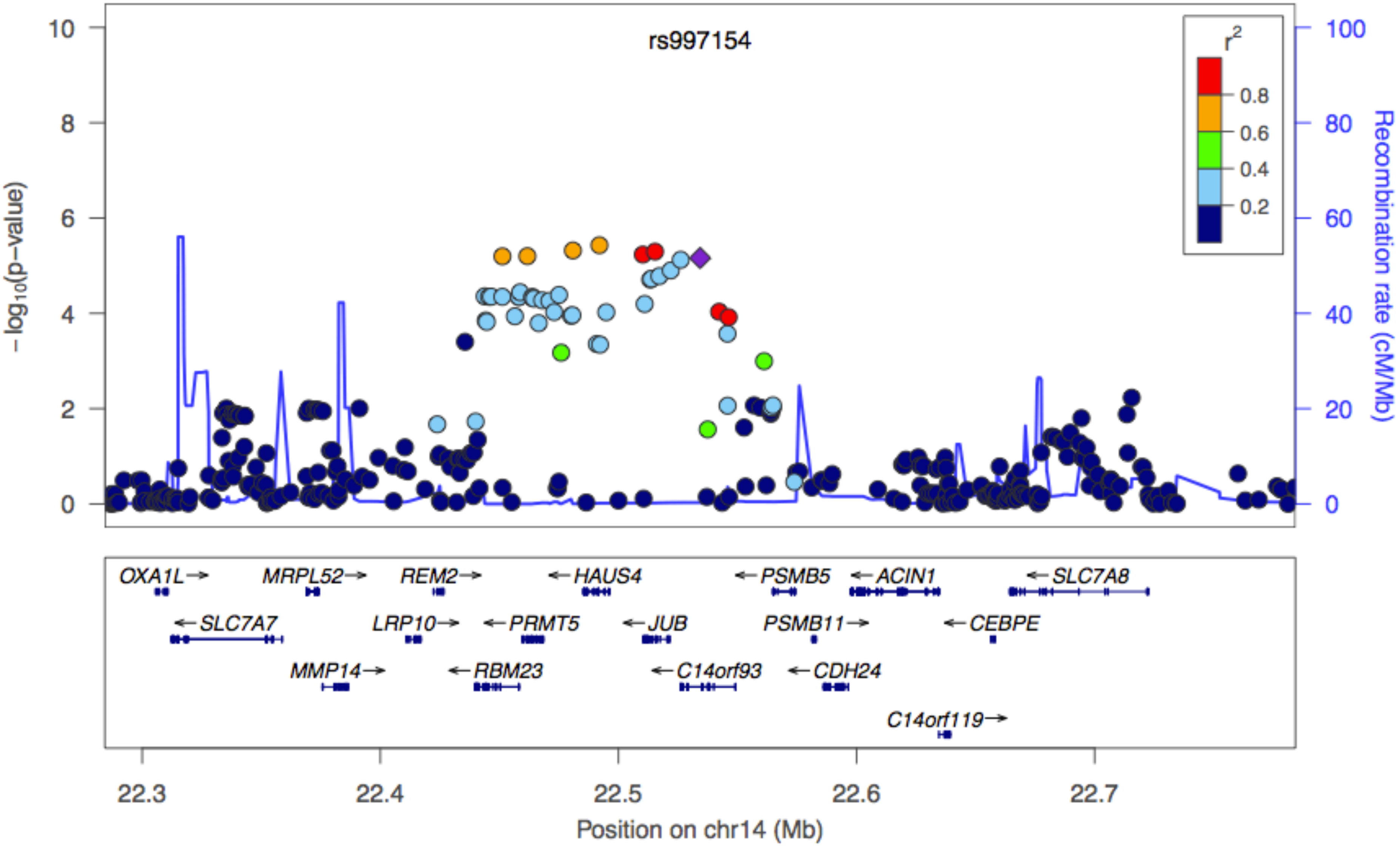

rs997154
r2
0.8
0.6
0.4
0.2
10
8
6
4
2
0
100
80
60
40
20
0
−log10(p−value)
Recombination rate (cM/Mb)
OXA1L→
MRPL52→
REM2→
←HAUS4
←PSMB5
←ACIN1
←SLC7A8
←SLC7A7
LRP10→
←PRMT5
←JUB
PSMB11→
←CEBPE
MMP14→
←RBM23
←C14orf93
←CDH24
C14orf119→
22.3
22.4
22.5
22.6
22.7
Position on chr14 (Mb)

(N)

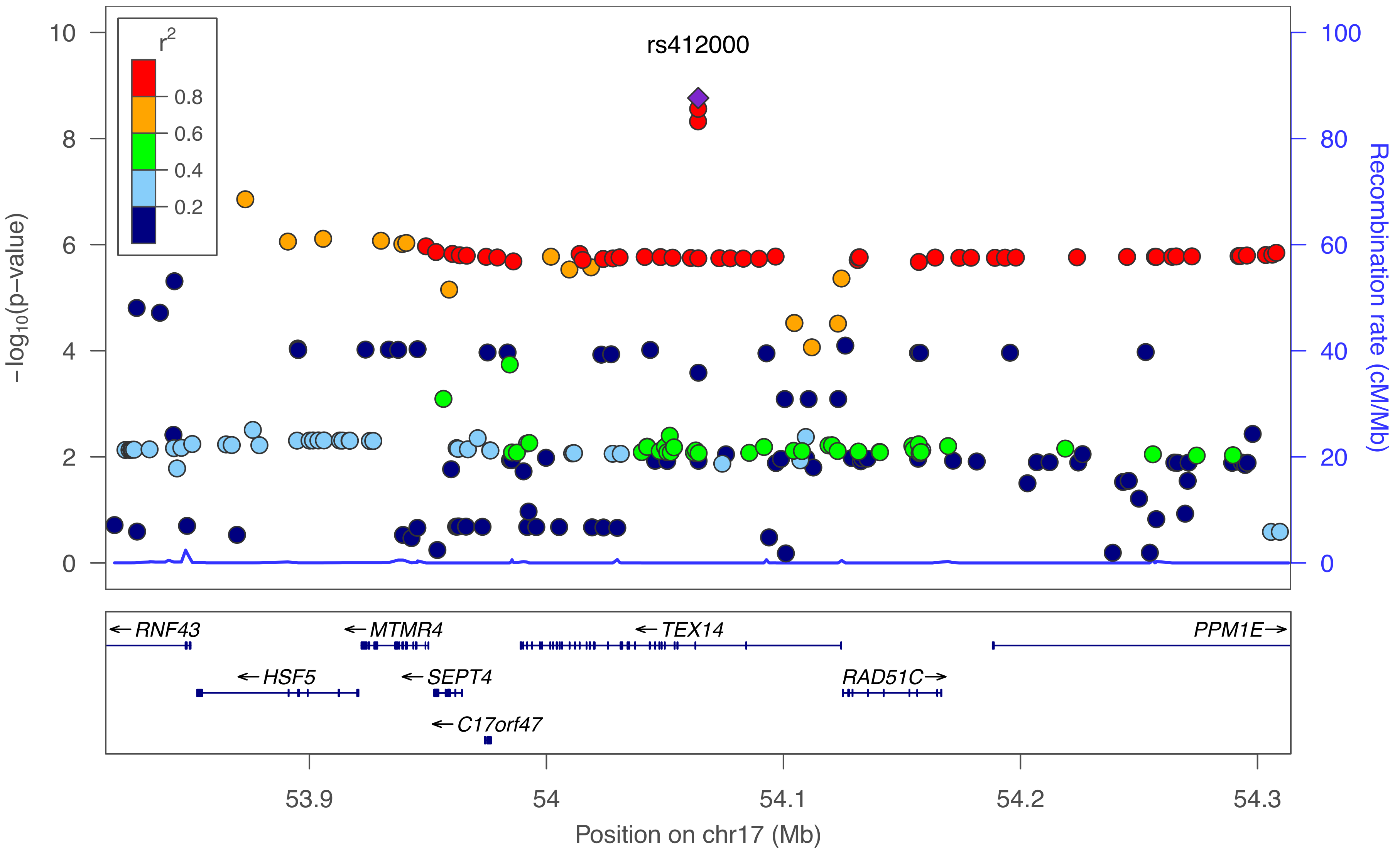

rs412000
r²
0.8
0.6
0.4
0.2
10
8
6
4
2
0
−log10(p−value)
100
80
60
40
20
0
Recombination rate (cM/Mb)
RNF43
MTMR4
TEX14
PPM1E
HSF5
SEPT4
RAD51C
C17orf47
53.9
54
54.1
54.2
54.3
Position on chr17 (Mb)

(O)

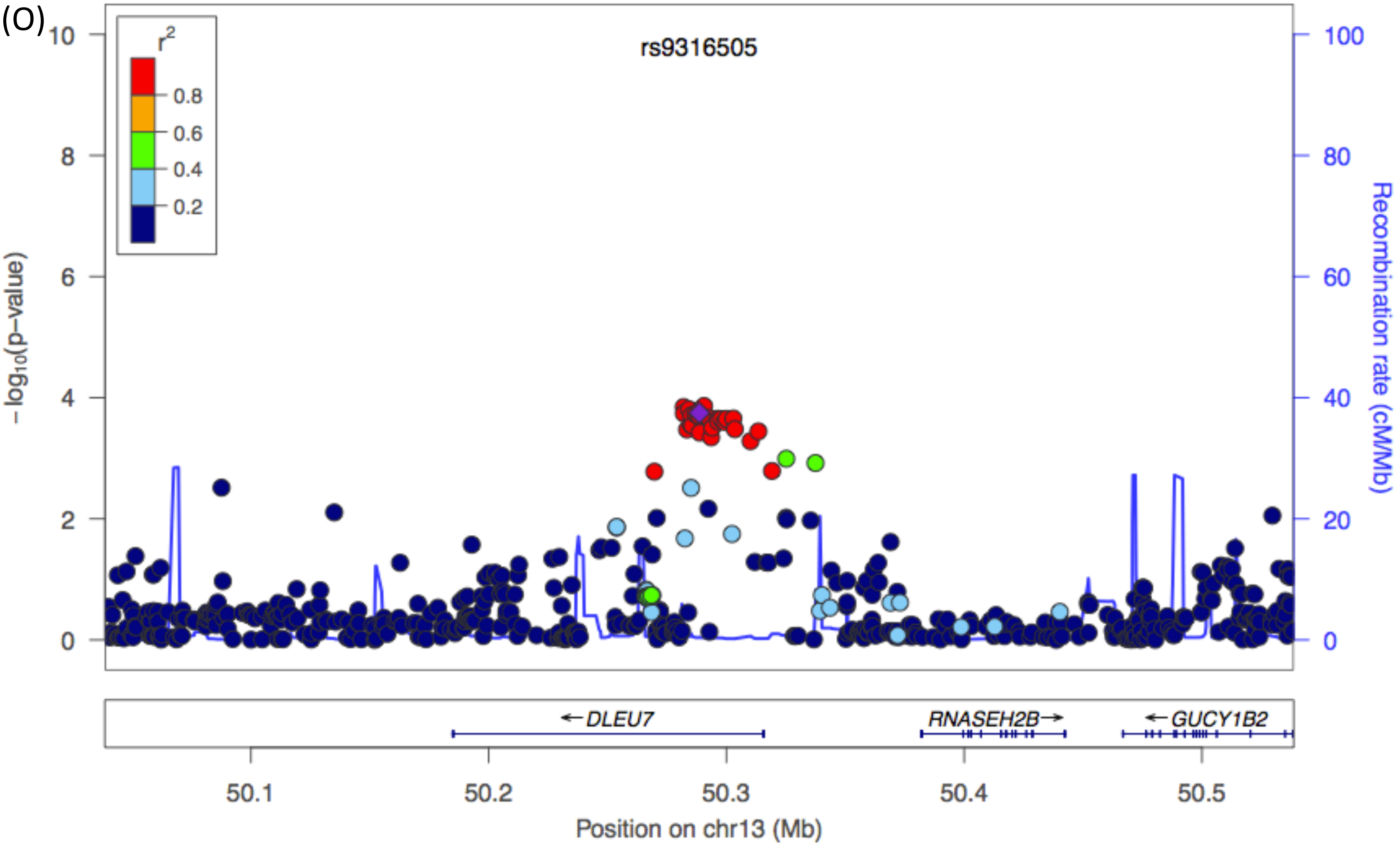


**Supplementary Figure 2**: Linkage disequilibrium and Association plot of loci reaching genome-wide significance for 'age at first tooth' in meta-analysis of ALSPAC and NFBC (A) rs8080944 *KCNJ16-KCNJ2* region (B) rs11796357 *FAM155- EDA* gene region (C) rs17563 *BMP4* gene region (D) rs1994969 *IGF2BP1* gene region (E) rs12229918 gene *MSRB3* (F) rs6568401 (G) rs7924176 *ADK* gene region (H) rs10740993 *CACNB2* gene region (I) rs4937076 *CDON* gene region. (J) rs17101923 *HMGA2* gene region (K) rs1799922 *CALU* (L)rs10932688 *2q35* region (M) rs997154 *AJUBA* gene region (N) rs412000 TEX14/RAD51C region (O) rs9316505 gene region. The most significant SNPs in each region plotted in purple. Genes are based on Genome browser (RefSeq Genes). Arrows on genes give direction of transcription.

(A)

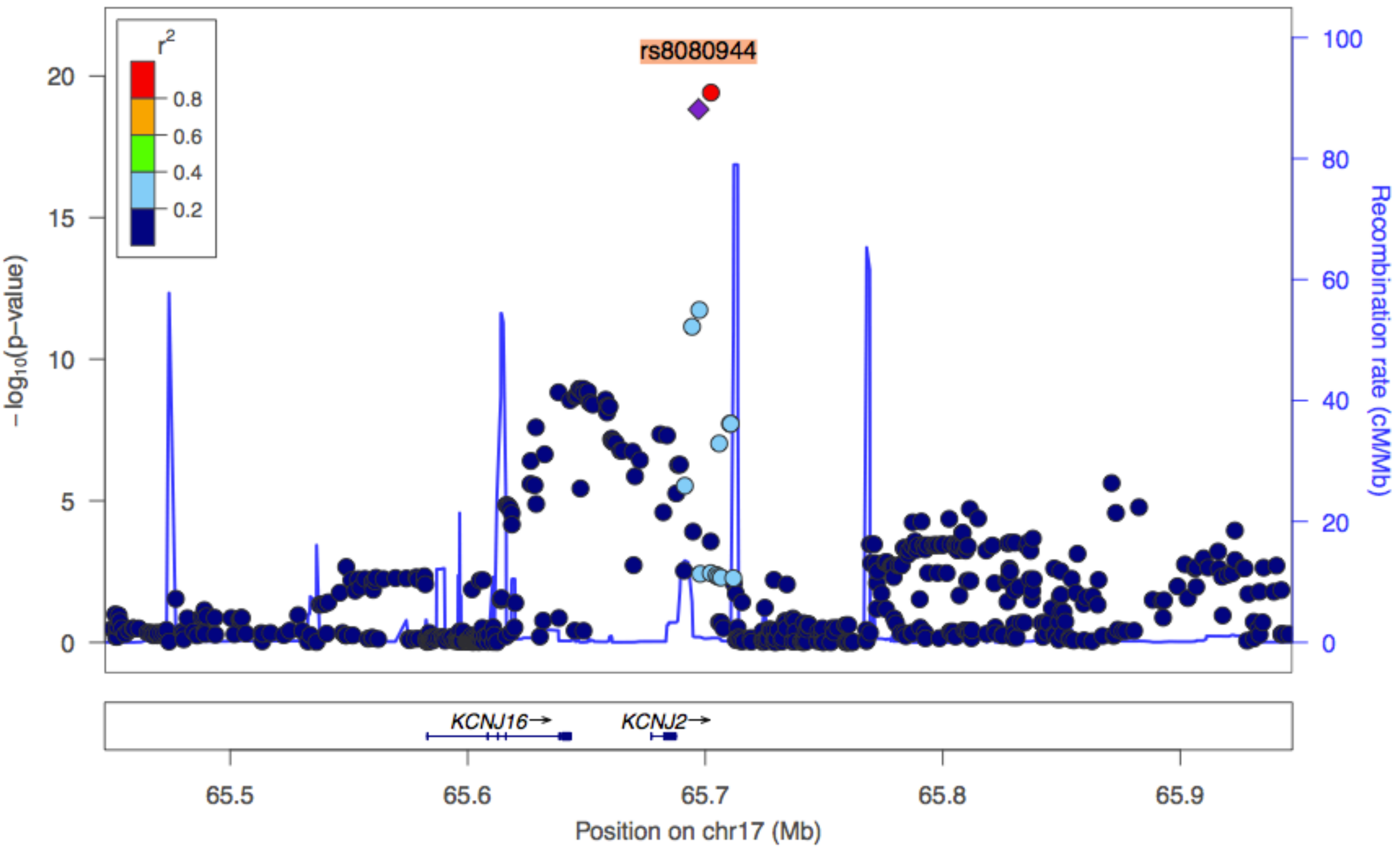

rs8080944
r²
0.8
0.6
0.4
0.2
20
15
10
5
0
− log10(p−value)
100
80
60
40
20
0
Recombination rate (cM/Mb)
KCNJ16→
KCNJ2→
65.5
65.6
65.7
65.8
65.9
Position on chr17 (Mb)

(B)

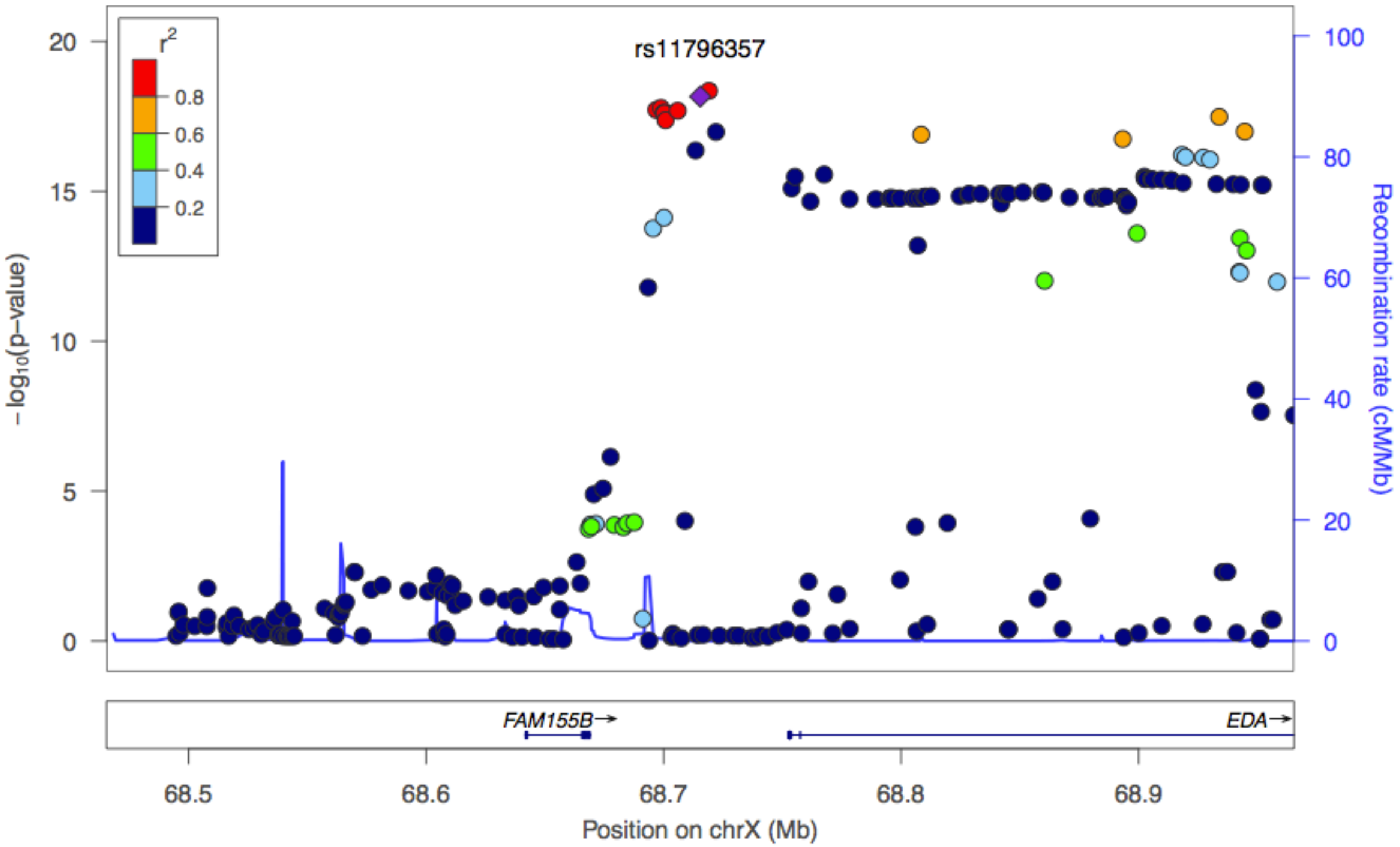

r²
0.8
0.6
0.4
0.2
rs11796357
20
15
10
5
0
−log10(p−value)
100
80
60
40
20
0
Recombination rate (cM/Mb)
FAM155B→
EDA→
68.5
68.6
68.7
68.8
68.9
Position on chrX (Mb)

(C)

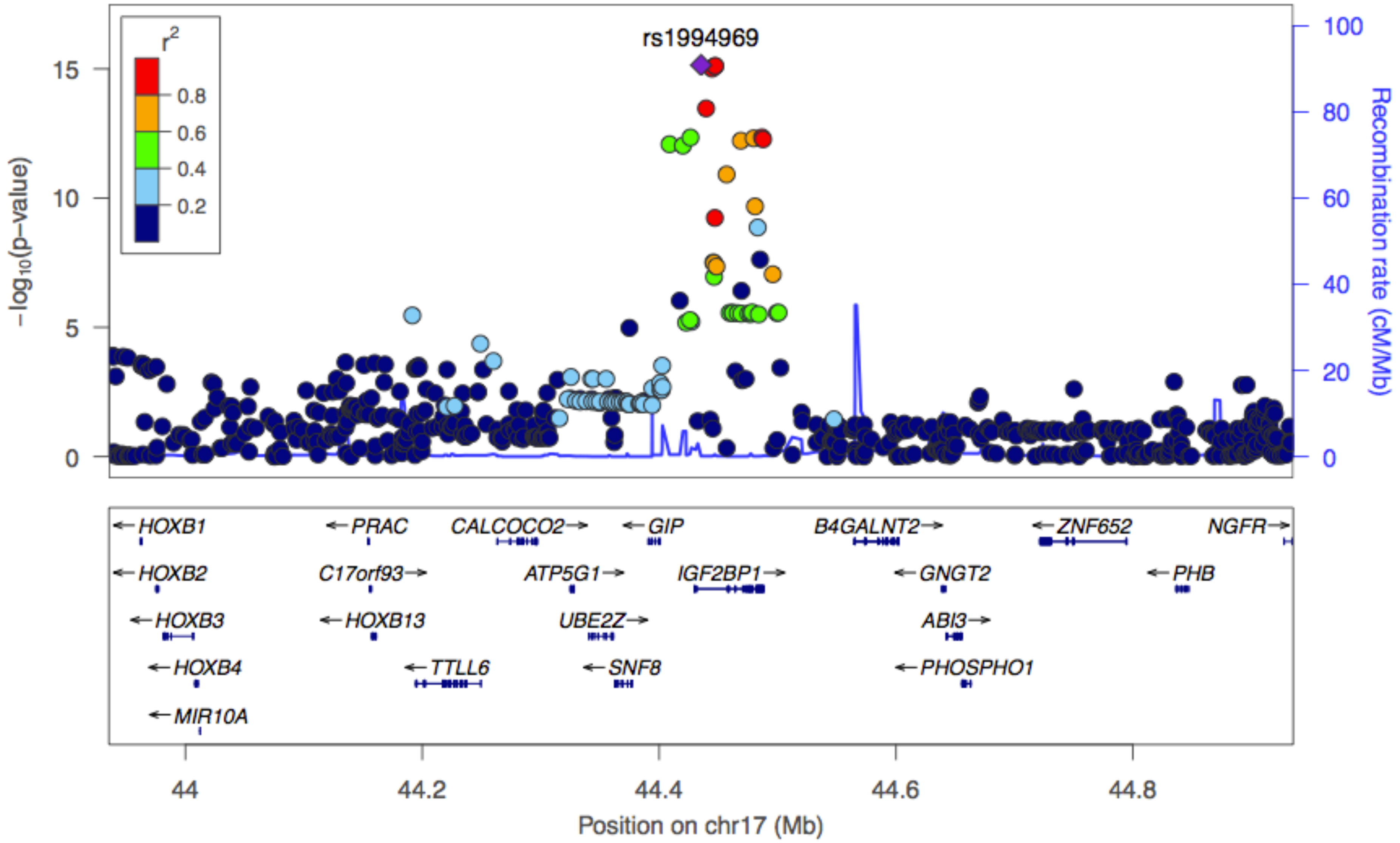

rs1994969
r2
0.8
0.6
0.4
0.2
−log10(p−value)
Recombination rate (cM/Mb)
HOXB1
PRAC
CALCOCO2
GIP
B4GALNT2
ZNF652
NGFR
HOXB2
C17orf93
ATP5G1
IGF2BP1
GNGT2
PHB
HOXB3
HOXB13
UBE2Z
ABI3
HOXB4
TTLL6
SNF8
PHOSPHO1
MIR10A
44
44.2
44.4
44.6
44.8
Position on chr17 (Mb)

(D)

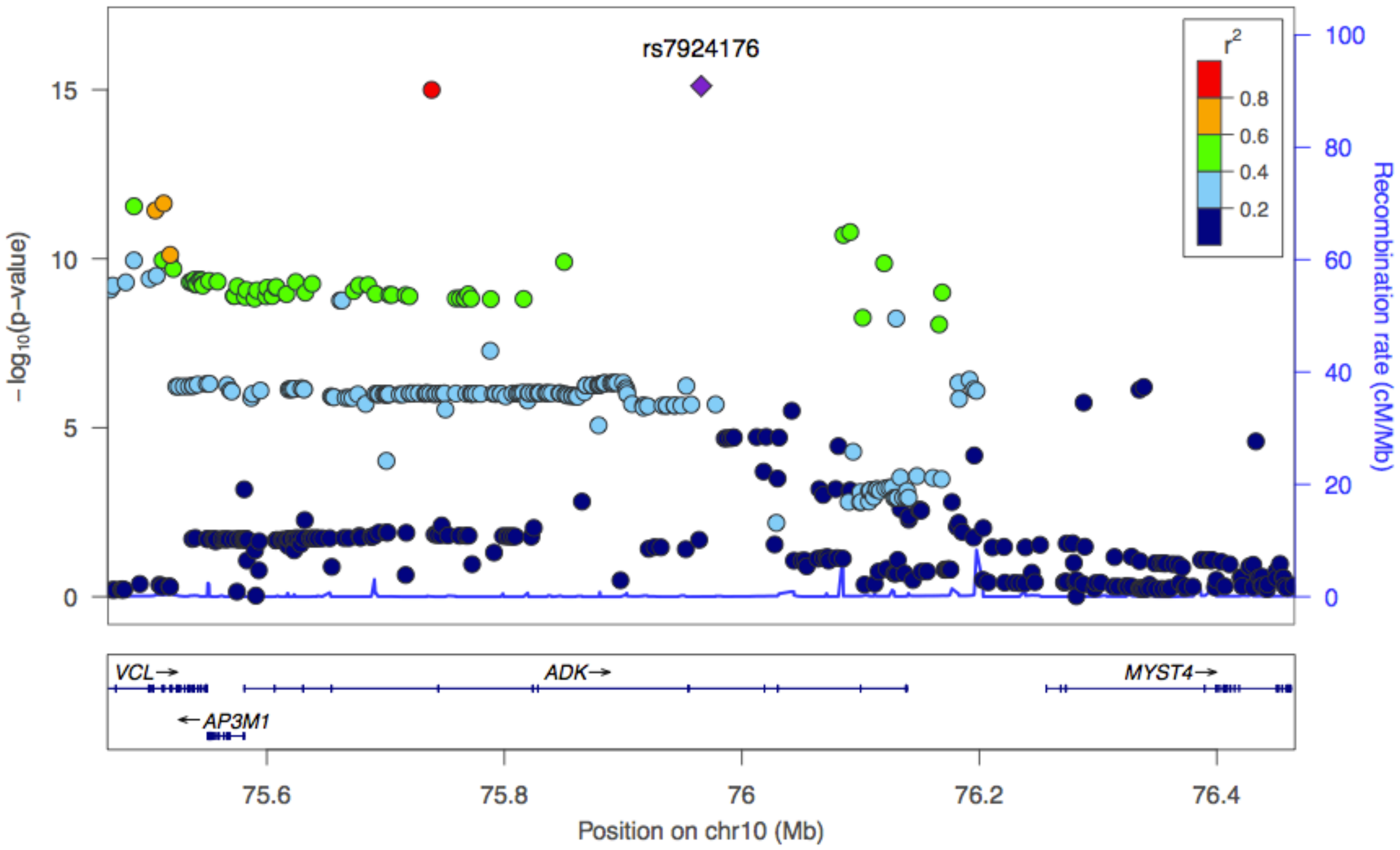
rs7924176
r²
0.8
0.6
0.4
0.2
15
10
5
0
100
80
60
40
20
0
− log10(p−value)
Recombination rate (cM/Mb)
VCL→
ADK→
MYST4→
←AP3M1
75.6
75.8
76
76.2
76.4
Position on chr10 (Mb)

(E)

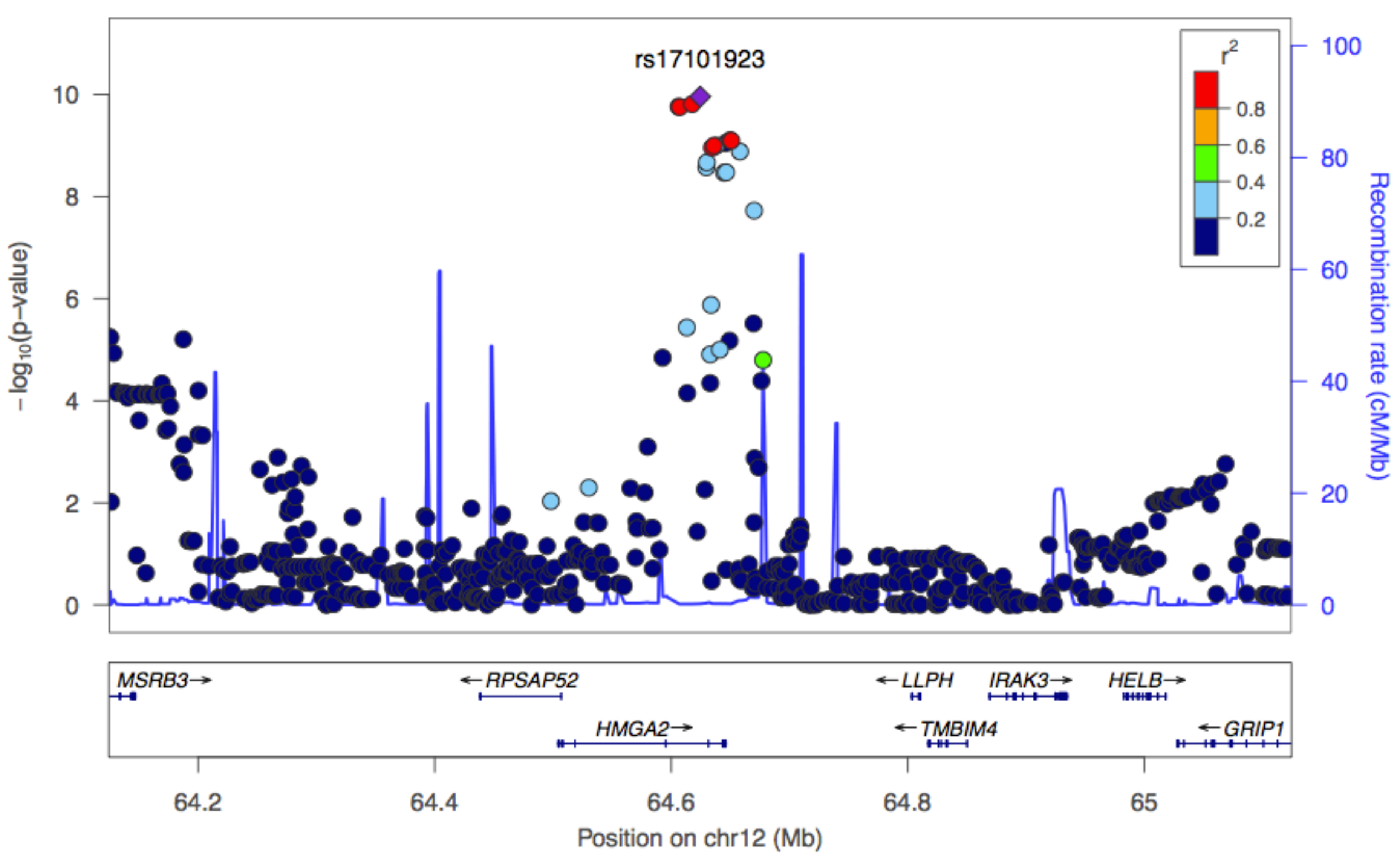

rs17101923
r2
0.8
0.6
0.4
0.2
10
8
6
4
2
0
- log10(p-value)
100
80
60
40
20
0
Recombination rate (cM/Mb)
MSRB3→
←RPSAP52
←LLPH
IRAK3→
HELB→
HMGA2→
←TMBIM4
←GRIP1
64.2
64.4
64.6
64.8
65
Position on chr12 (Mb)

(F)

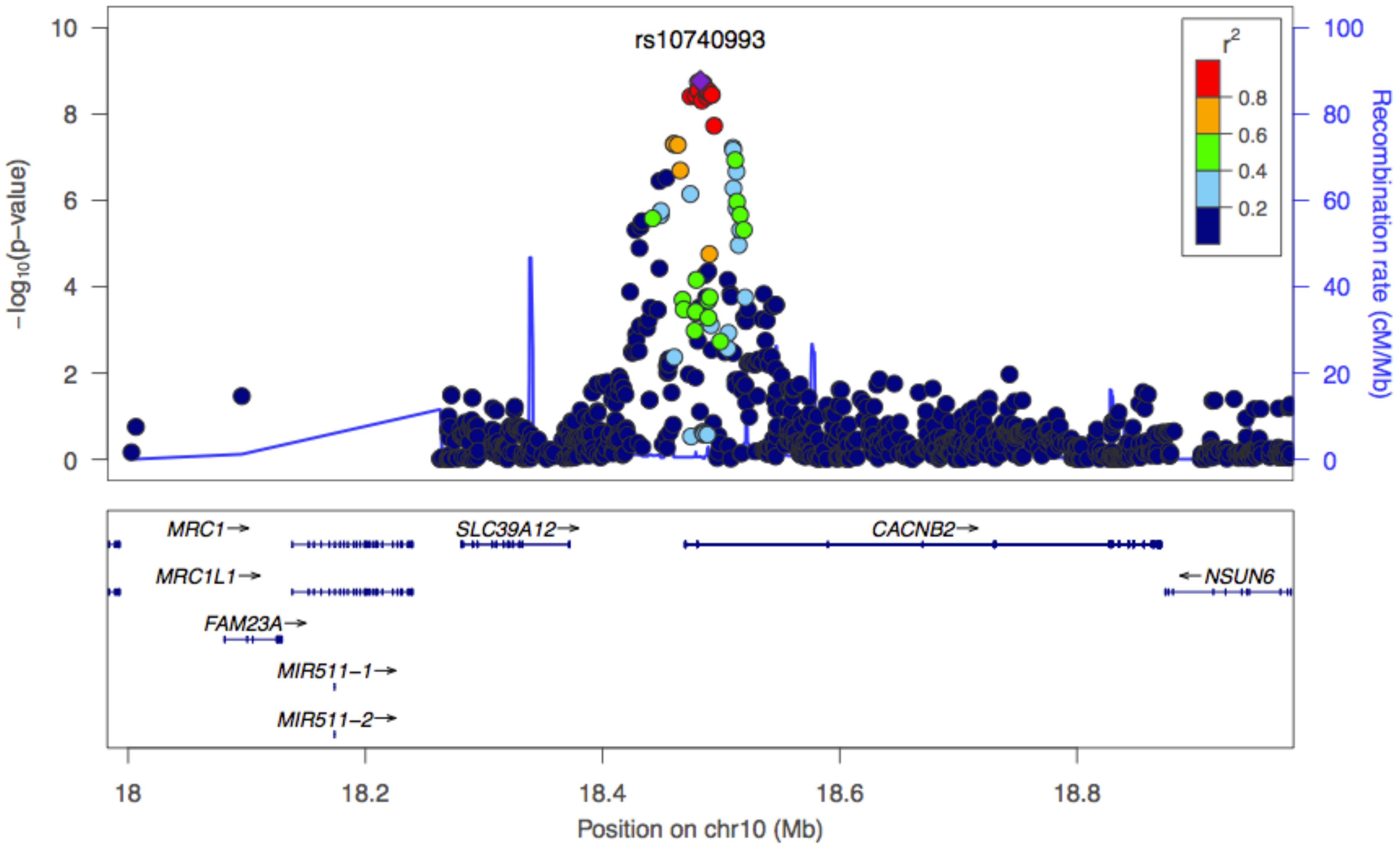
rs10740993
r2
0.8
0.6
0.4
0.2
−log10(p−value)
Recombination rate (cM/Mb)
10
8
6
4
2
0
100
80
60
40
20
0
MRC1→
MRC1L1→
FAM23A→
MIR511−1→
MIR511−2→
SLC39A12→
CACNB2→
←NSUN6
18
18.2
18.4
18.6
18.8
Position on chr10 (Mb)

(G)

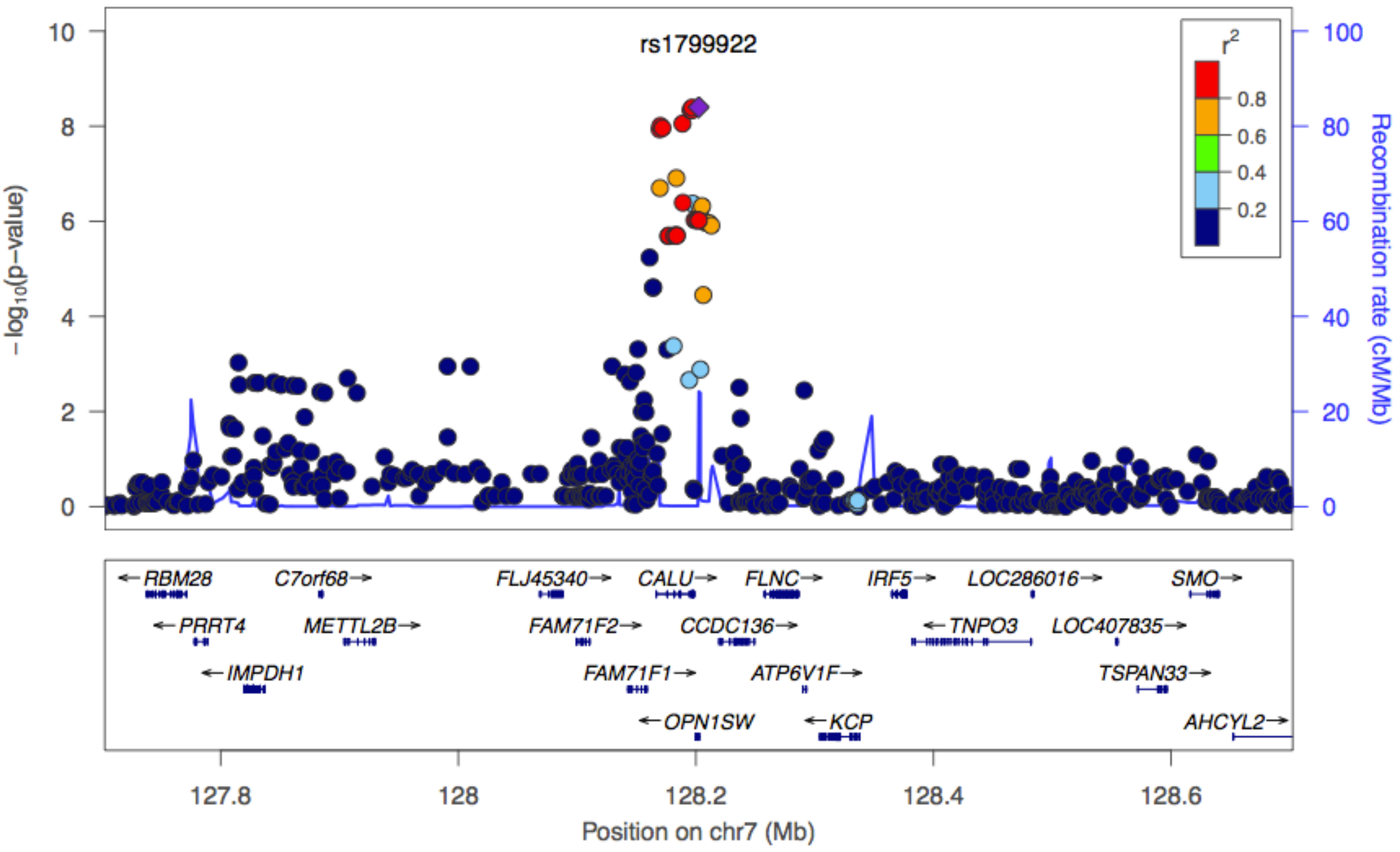
rs1799922
r2
0.8
0.6
0.4
0.2
10
8
6
4
2
0
100
80
60
40
20
0
− log10(p−value)
Recombination rate (cM/Mb)
←RBM28
C7orf68→
FLJ45340→
CALU→
FLNC→
IRF5→
LOC286016→
SMO→
←PRRT4
METTL2B→
FAM71F2→
CCDC136→
←TNPO3
LOC407835→
←IMPDH1
FAM71F1→
ATP6V1F→
TSPAN33→
←OPN1SW
←KCP
AHCYL2→
127.8
128
128.2
128.4
128.6
Position on chr7 (Mb)

(H)

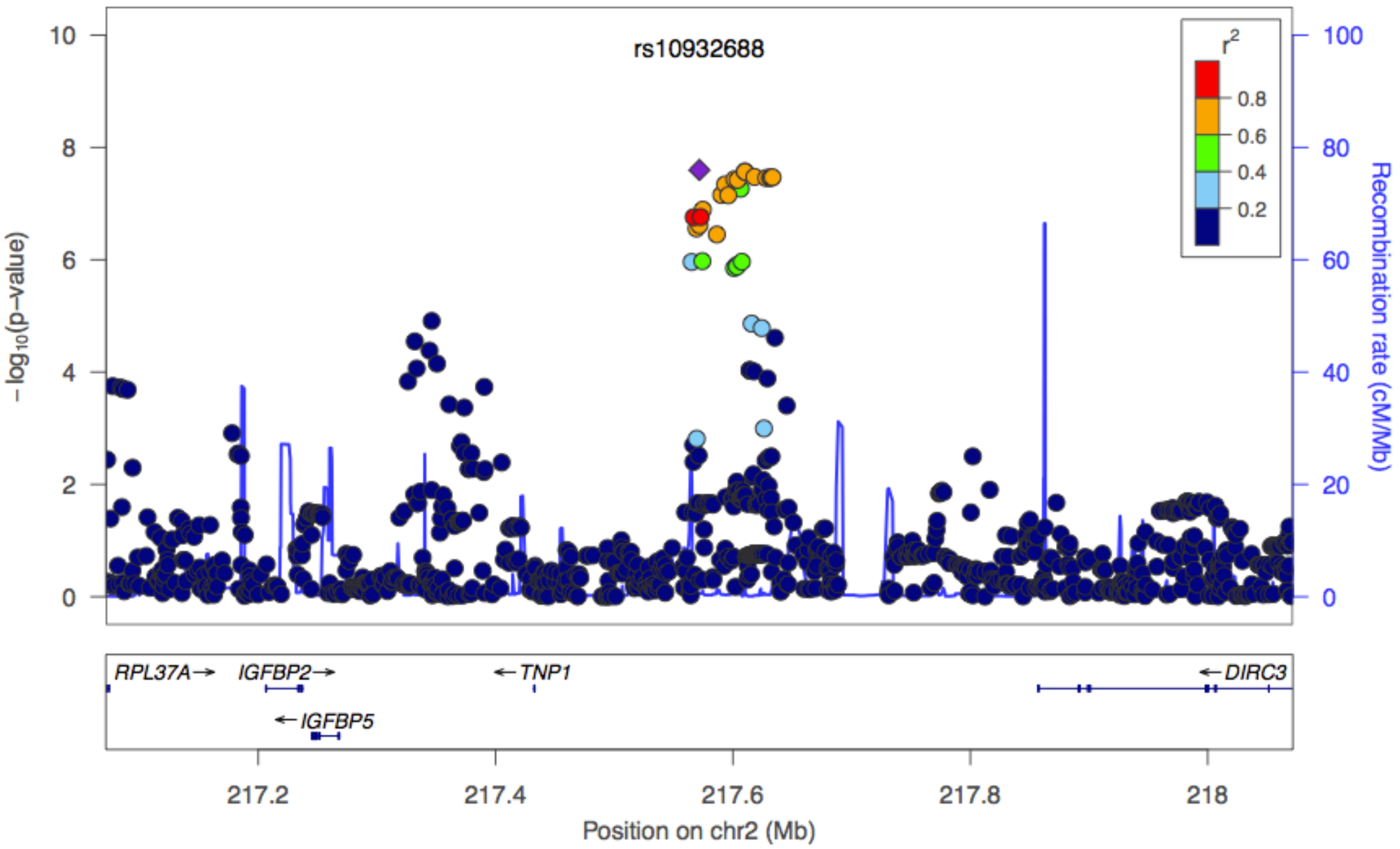
rs10932688
r2
0.8
0.6
0.4
0.2
10
8
6
4
2
0
− log10(p−value)
100
80
60
40
20
0
Recombination rate (cM/Mb)
RPL37A→
IGFBP2→
←IGFBP5
←TNP1
←DIRC3
217.2
217.4
217.6
217.8
218
Position on chr2 (Mb)

(I)

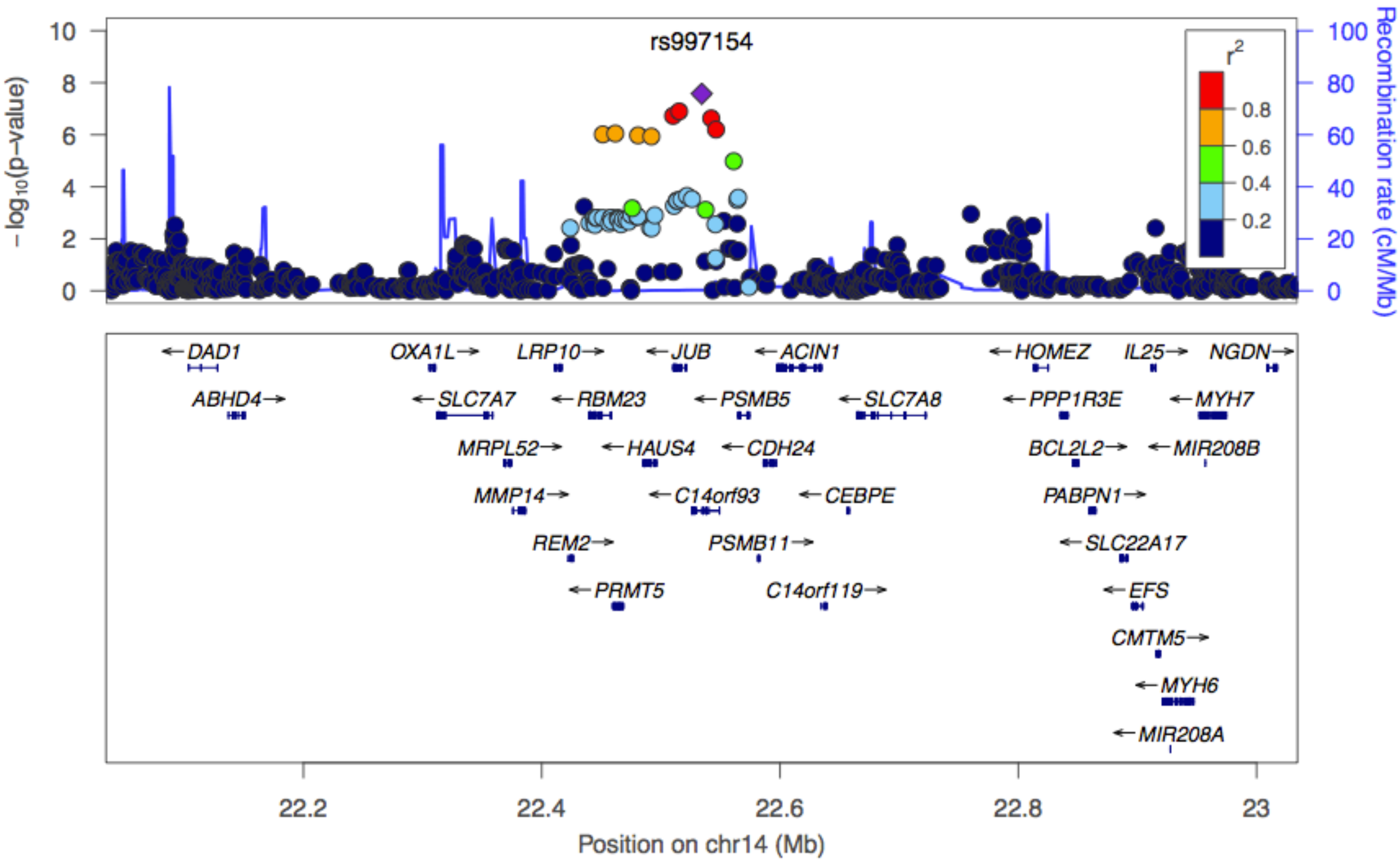

rs997154
−log10(p−value)
Recombination rate (cM/Mb)
r2
0.8
0.6
0.4
0.2
DAD1
ABHD4
OXA1L
SLC7A7
MRPL52
MMP14
LRP10
RBM23
REM2
PRMT5
HAUS4
JUB
C14orf93
PSMB5
CDH24
PSMB11
ACIN1
C14orf119
CEBPE
SLC7A8
HOMEZ
PPP1R3E
BCL2L2
PABPN1
SLC22A17
EFS
CMTM5
MYH6
MIR208A
IL25
NGDN
MYH7
MIR208B
22.2
22.4
22.6
22.8
23
Position on chr14 (Mb)

(J)

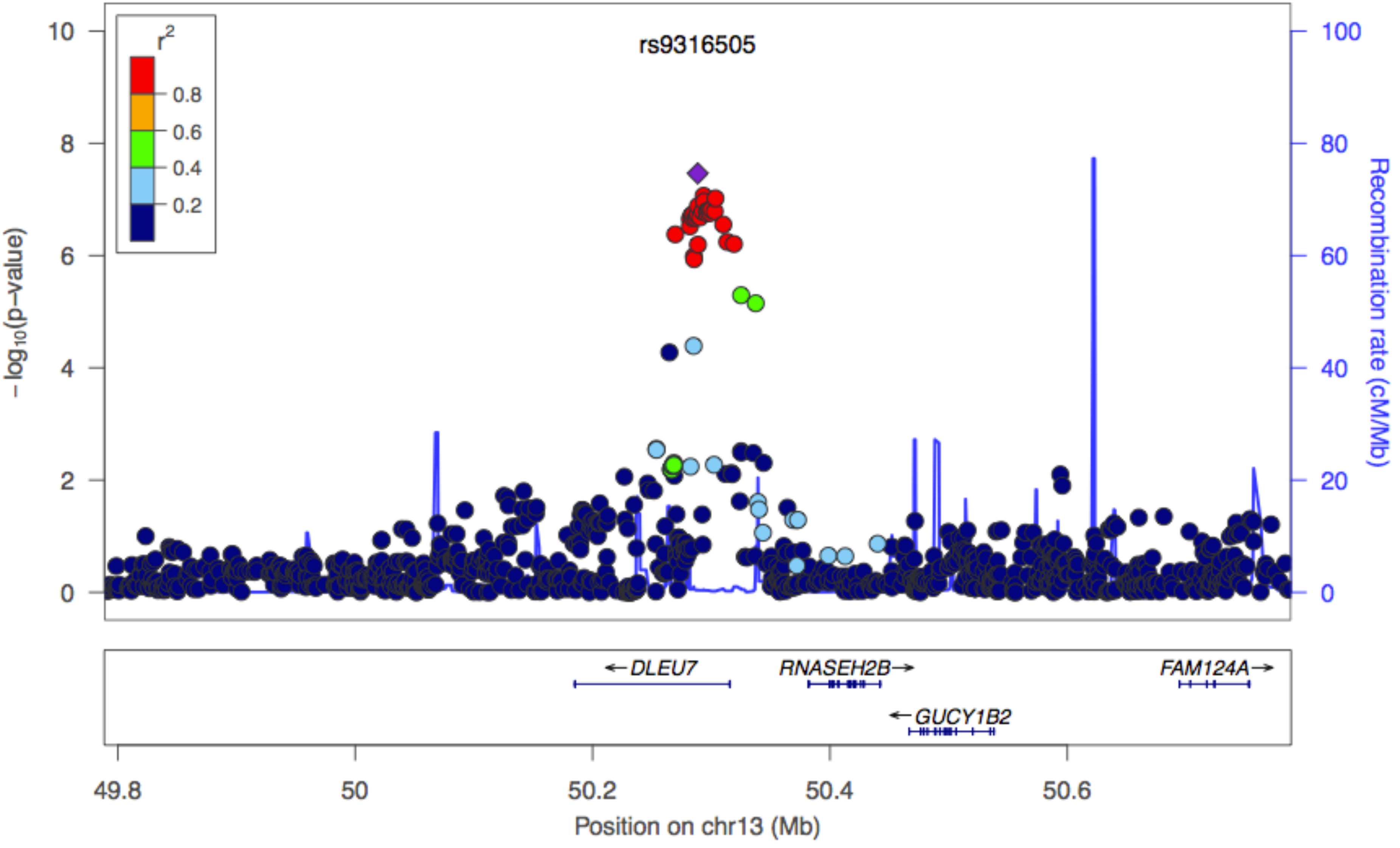
rs9316505
r2
0.8
0.6
0.4
0.2
10
8
6
4
2
0
- log10(p-value)
100
80
60
40
20
0
Recombination rate (cM/Mb)
← DLEU7
RNASEH2B →
FAM124A →
← GUCY1B2
49.8
50
50.2
50.4
50.6
Position on chr13 (Mb)

(K)

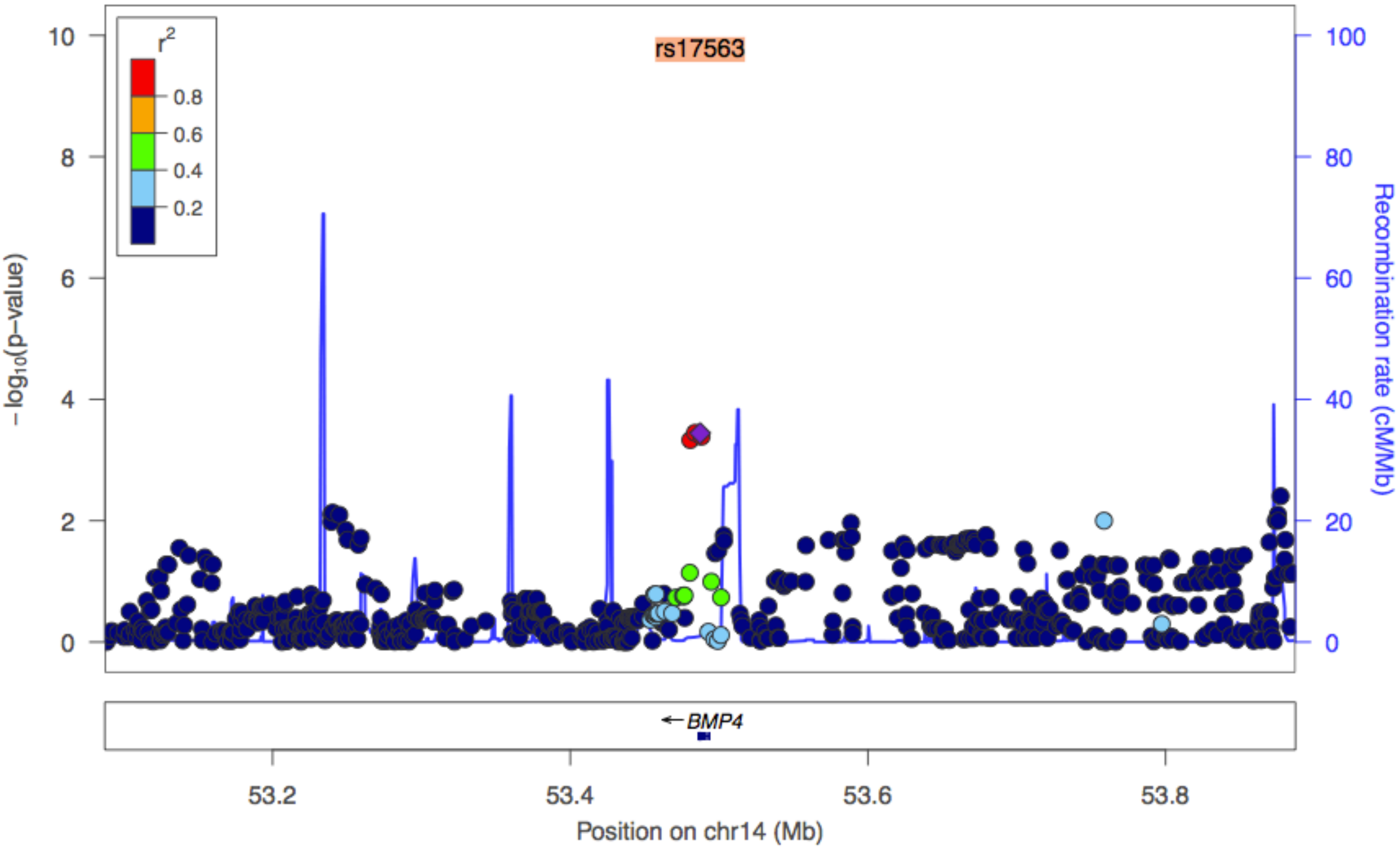

rs17563
r^2
0.8
0.6
0.4
0.2
10
8
6
4
2
0
- log10(p-value)
100
80
60
40
20
0
Recombination rate (cM/Mb)
← BMP4
53.2
53.4
53.6
53.8
Position on chr14 (Mb)

(L)

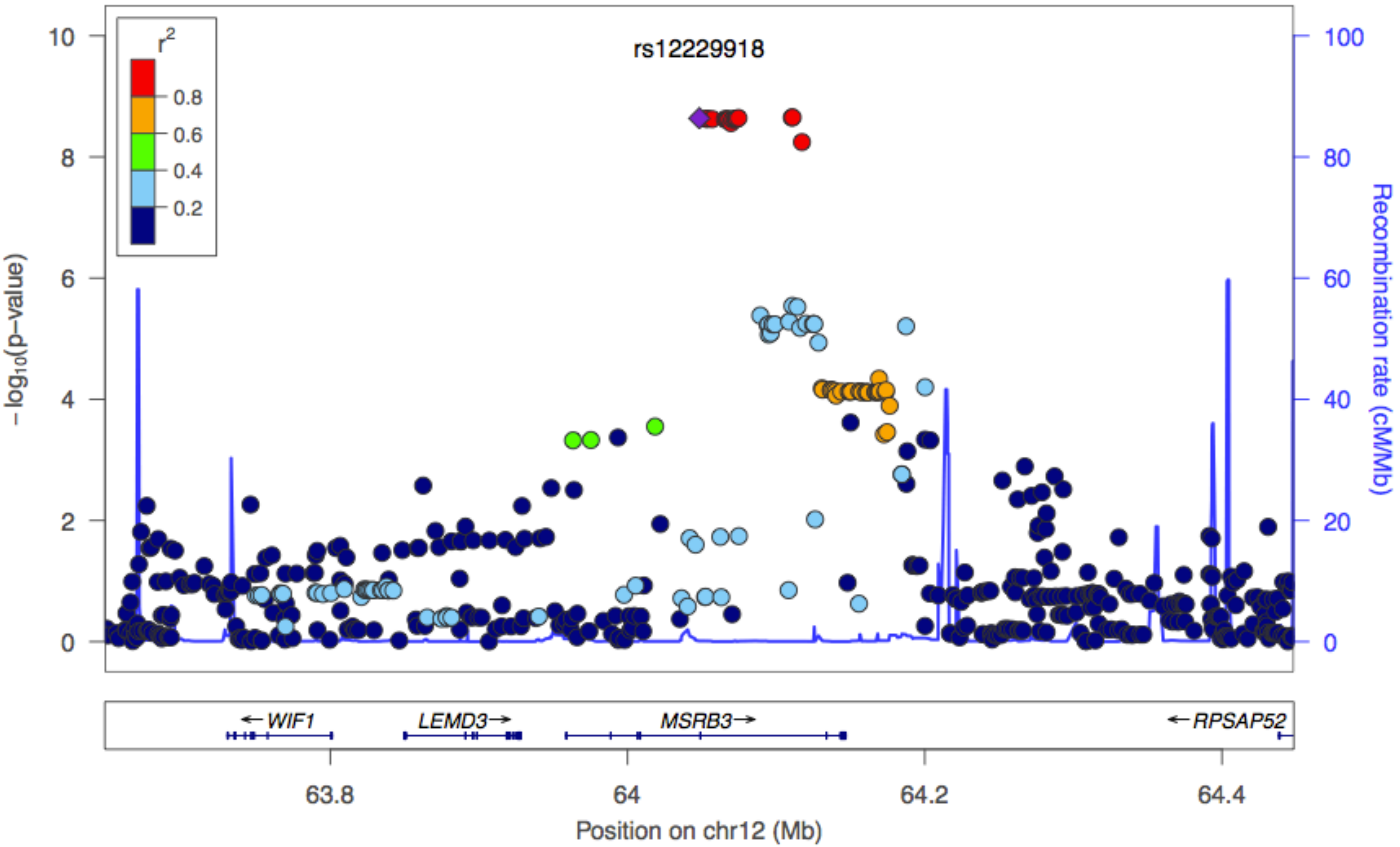

rs12229918
r2
0.8
0.6
0.4
0.2
10
8
6
4
2
0
100
80
60
40
20
0
− log10(p−value)
Recombination rate (cM/Mb)
← WIF1
LEMD3→
MSRB3→
← RPSAP52
63.8
64
64.2
64.4
Position on chr12 (Mb)

(M)

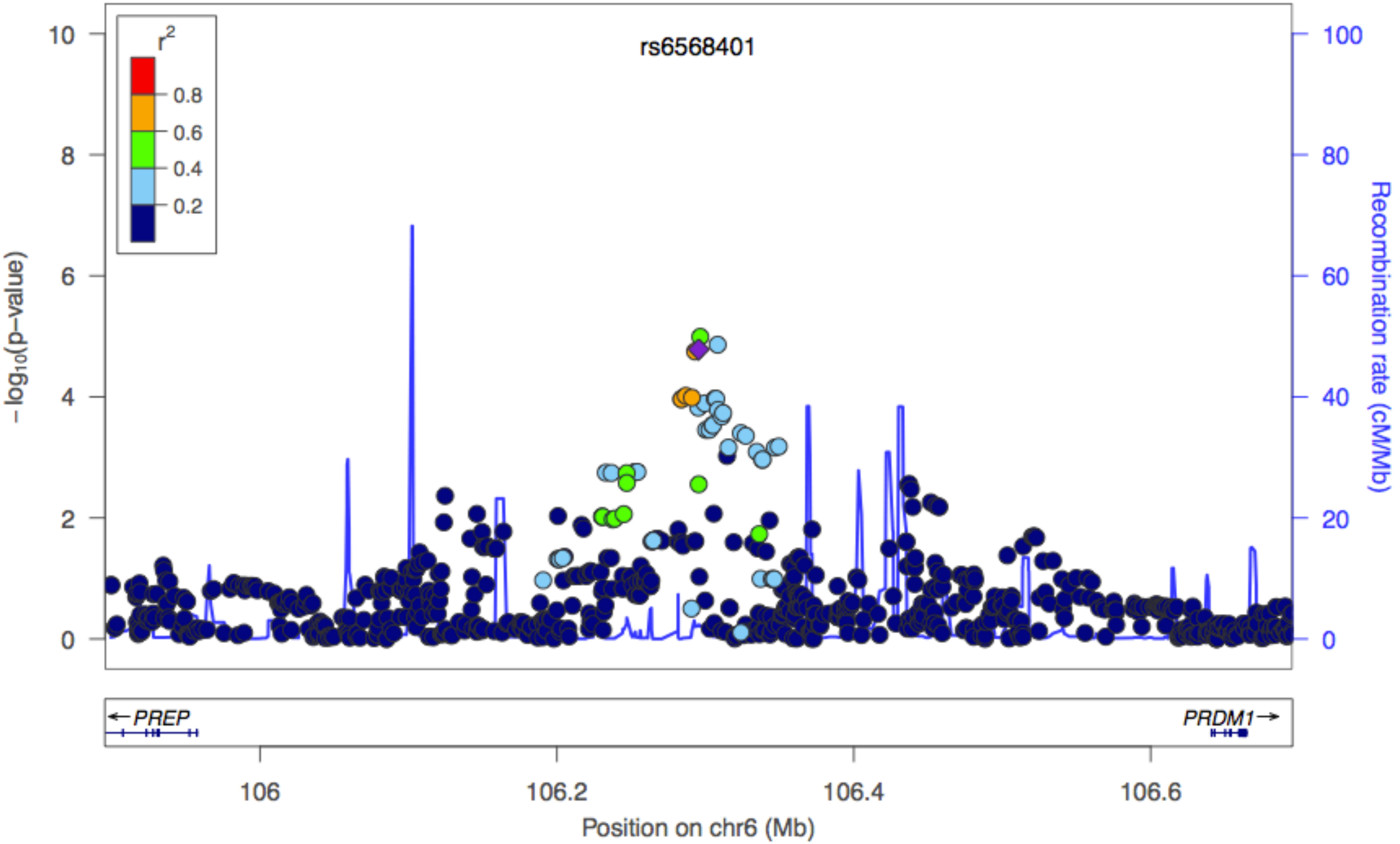
rs6568401
r²
0.8
0.6
0.4
0.2
10
8
6
4
2
0
- log₁₀(p-value)
100
80
60
40
20
0
Recombination rate (cM/Mb)
PREP
PRDM1
106
106.2
106.4
106.6
Position on chr6 (Mb)

(N)

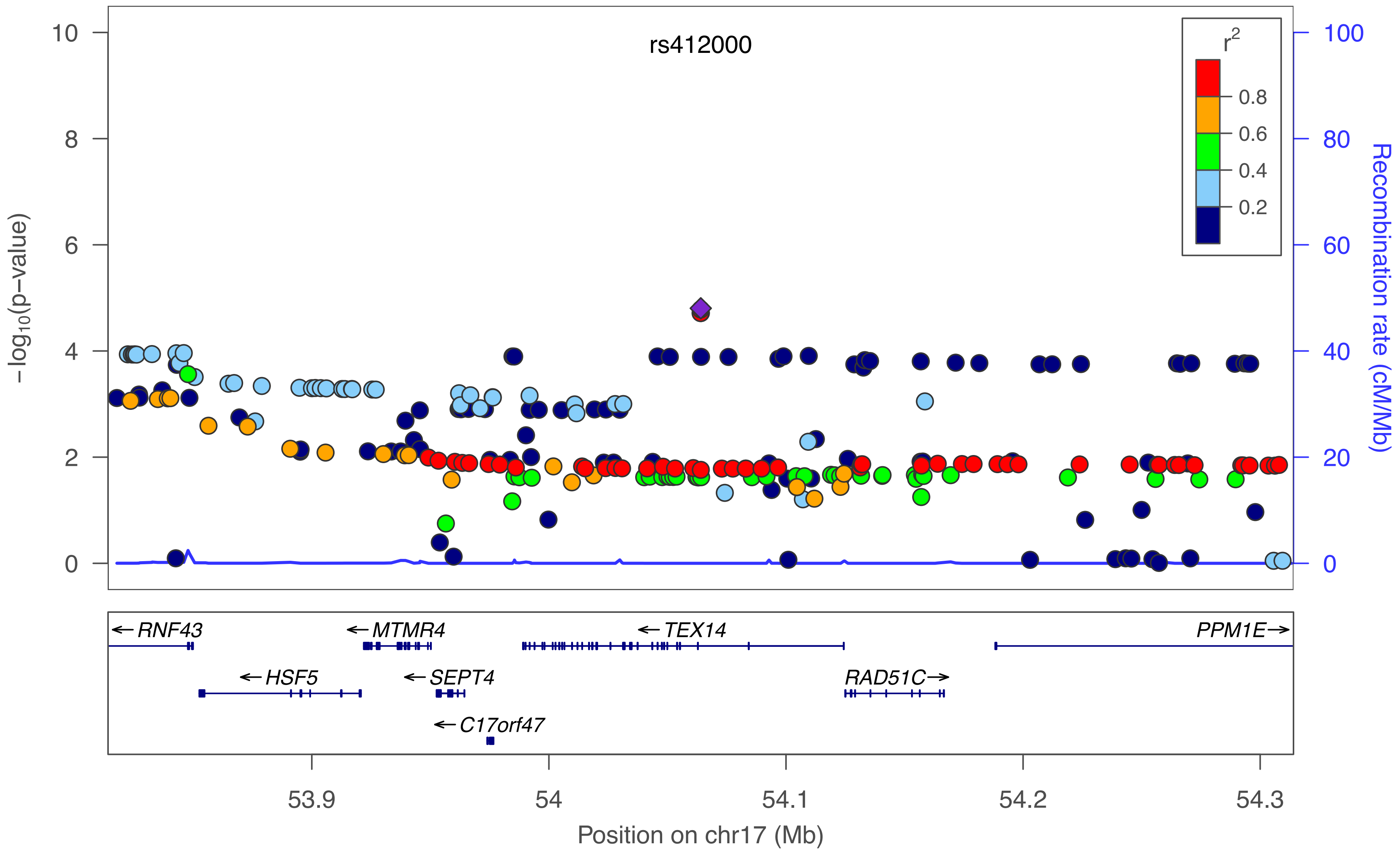

rs412000
r2
0.8
0.6
0.4
0.2
10
8
6
4
2
0
−log10(p−value)
100
80
60
40
20
0
Recombination rate (cM/Mb)
← RNF43
← MTMR4
← TEX14
PPM1E→
← HSF5
← SEPT4
RAD51C→
← C17orf47
53.9
54
54.1
54.2
54.3
Position on chr17 (Mb)

(O)

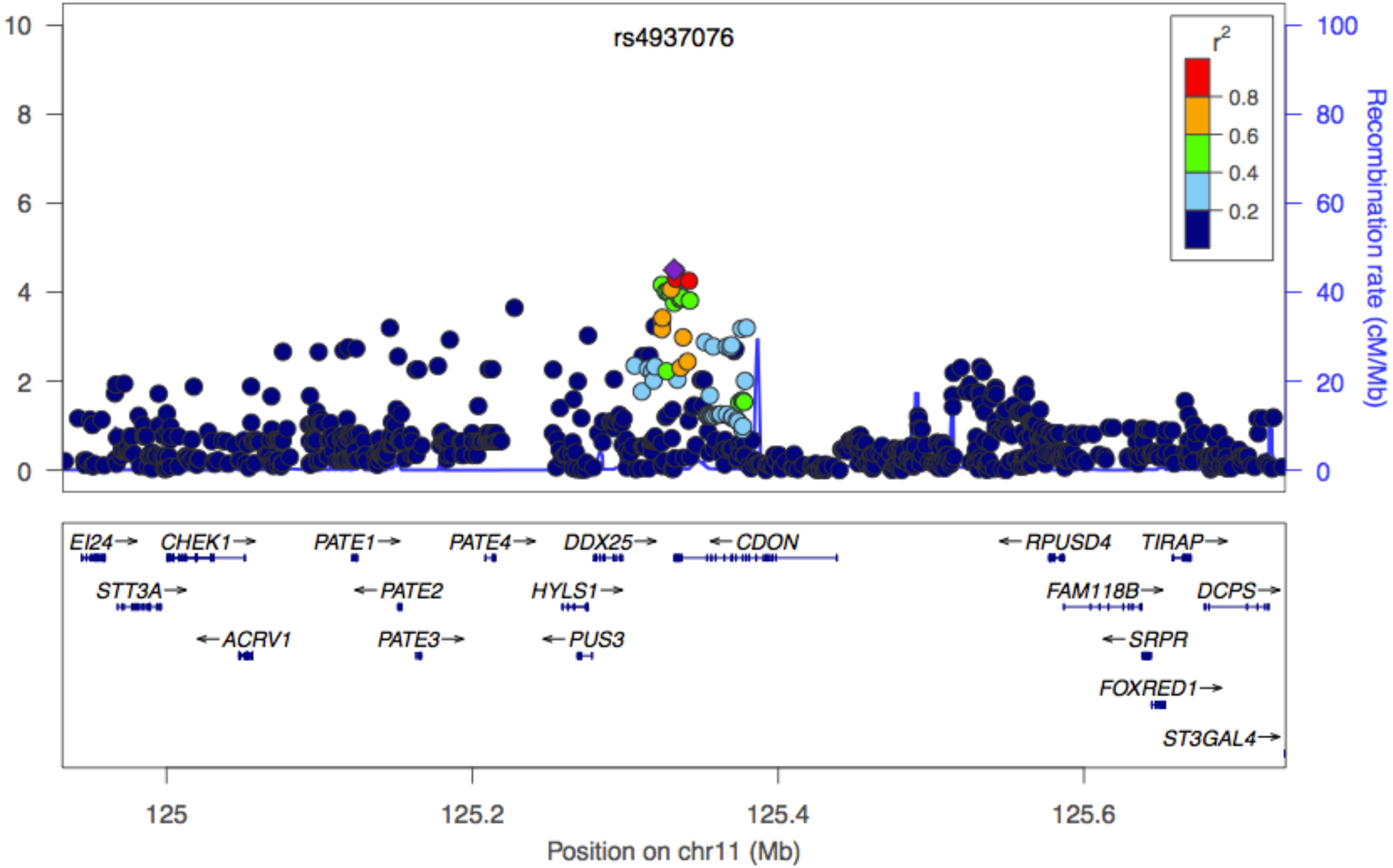


**Supplementary Figure 3**: Linkage disequilibrium and Association plot of loci reaching genome-wide significance for 'number of teeth' at one year in meta-analysis of ALSPAC and NFBC. (A) rs8080944 *KCNJ16-KCNJ2* region (B) rs11796357 *FAM155- EDA* gene region (C) rs1994969 IGF3BP1 gene region (D) rs7924176 ADK gene region (E) rs17101923 HMGA2 gene region (F) rs10740993 CACNB2 gene region (G) rs1799922 CALU (H) rs10932688 2q35 (I) rs997154 AJUBA gene region (J) rs9316505 DLEU7 gene region (K) rs17563 *BMP4* gene region (L) rs12229918 *MSRB3* gene region (M) rs6568401 region(N) rs412000 TEX14/RAD51C region (O) rs4937076 *CDON* gene region. The most significant SNPs in each region plotted in purple. Genes are based on Genome browser (RefSeq Genes). Arrows on genes give direction of transcription.

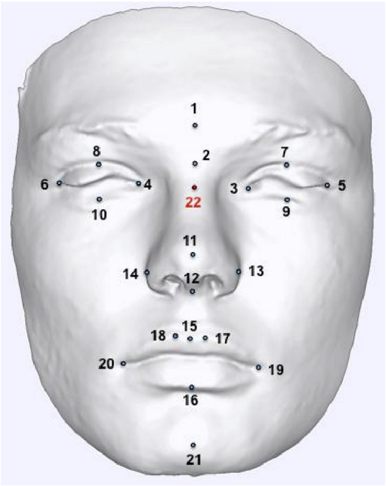


| No | Abbreviation | Name |
|---|---|---|
| 1 | g | Glabella |
| 2 | n | Nasion |
| 3 | enL | Left endocanthion |
| 4 | enR | Right endocanthion |
| 5 | exL | Left exocanthion |
| 6 | exR | Right exocanthion |
| 7 | psL | Left palpebrale superius |
| 8 | psR | Right palpebrale superius |
| 9 | piL | Left palpebrale inferius |
| 10 | piR | Right palpebrale inferius |
| 11 | prn | Pronasale |
| 12 | sn | Subnasale |
| 13 | alL | Left alare |
| 14 | alR | Right alare |
| 15 | ls | Labiale superius |
| 16 | li | Labiale inferius |
| 17 | cphL | Left crista philtri |
| 18 | cphR | Right crista philtri |
| 19 | chL | Left cheilion |
| 20 | chR | Right cheilion |
| 21 | pg | Pogonion |
| 22 | men | Mid-endocanthion point |

| Forehead & Eyes | | Lips & Mouth | | Nose | | Chin | |
|---|---|---|---|---|---|---|---|
| 1-22 | g-men* | 12-15 | sn-ls | 2-22 | n-men* | 21-22 | pg-men* |
| 1-2 | g-n | 15-16 | ls-li | 12-22 | sn-men* | 21-1 | pg-g |
| 3-4 | enL-enR | 16-21 | li-pg | 2-11 | n-prn | 21-2 | pg-n |
| 5-6 | exL-exR | 15-21 | ls-pg | 2-12 | n-sn | 21-12 | pg-sn |
| 7-8 | psL-psR | 15-17 | ls-cphL | 2-13 | n-alL | 21-19 | pg-chL |
| 9-10 | piL-piR | 15-18 | ls-cphR | 2-14 | n-alR | 21-20 | pg-chR |
| 3-5 | enL-exL | 17-18 | cphL-cphR | 13-14 | alL-alR | | |
| 4-6 | enR-exR | 19-20 | chL-chR | 11-12 | prn-sn | | |
| 7-9 | psL-piL | 19-17 | chL-cphL | 11-13 | prn-alL | | |
| 8-10 | psR-piR | 19-16 | chL-li | 11-14 | prn-alR | | |
| 5-XZ | exL-xz | 20-18 | chR-cphR | 12-13 | sn-alL | | |
| 6-XZ | exR-xz | 20-16 | chR-li | 12-14 | sn-alR | | |
| 5-XY | exL-xy | 15-22 | ls-men* | | | | |
| 6-XY | exR-xy | 16-22 | li-men* | | | | |
| 5-YZ | exL-yz | | | | | | |
| 6-YZ | exR-yz | | | | | | |
| 3-XZ | enL-xz | | | | | | |
| 4-XZ | enR-xz | | | | | | |
| 3-XY | enL-xy | | | | | | |
| 4-XY | enR-xy | | | | | | |
| 3-YZ | enL-yz | | | | | | |
| 4-YZ | enR-yz | | | | | | |

**Supplementary Figure 4: Position and Definition of the 22 Landmarks and the 54 parameters analysed in Paternoster et al. (2012):** Parameters with pairs of numbers denote the direct 3D distance between pairs of landmarks. Those with "xz", "xy" or "yz" denote the prominence of the landmark from the xz, xy, or yz planes. *"men" (point 22) denotes the midendocanthion or midintercanthal point (the midpoint between left and right endocanthi); this point does not lie on the facial surface

| MARKER | GENE REGION/LOCUS | CHR | BP | A1 | A2 | EFFECT ALSPAC | SE ALSPAC | PVALUE ALSPAC | EFFECT NFBC | SE NFBC | PVALUE NFBC | EFFECT META | SE META | PVALUE META |
|---|---|---|---|---|---|---|---|---|---|---|---|---|---|---|
| rs2057579 | TMEM9 | 1 | 199365898 | **T** | C | -0.067 | 0.045 | 1.4x10-01 | -0.176 | 0.036 | 1.3x10-06 | -0.133 | 0.028 | 2.6x10-06 |
| rs11956881 | PP187 | 5 | 127951662 | **C** | T | -0.26 | 0.091 | 4.4x10-03 | -0.324 | 0.078 | 3.4x10-05 | -0.297 | 0.059 | 5.7x10-07 |
| rs9388501 | AK127472 | 6 | 126944704 | **C** | T | -0.236 | 0.043 | 2.7x10-08 | -0.085 | 0.035 | 1.3x10-02 | -0.145 | 0.027 | 5.9x10-08 |
| rs10225279 | CR623750 | 7 | 19099597 | **T** | G | -0.19 | 0.043 | 1.0x10-05 | -0.117 | 0.036 | 9.5x10-04 | -0.147 | 0.027 | 8.5x10-08 |
| rs339039 | OPN1SW | 7 | 128203797 | **A** | G | -0.176 | 0.045 | 9.4x10-05 | -0.149 | 0.043 | 6.0x10-04 | -0.162 | 0.031 | 2.2x10-07 |
| rs1157136 | CR600271,CR593328 | 8 | 129971092 | **T** | C | -0.181 | 0.043 | 2.4x10-05 | -0.131 | 0.038 | 5.0x10-04 | -0.153 | 0.028 | 6.5x10-08 |
| rs10824218 | ADK | 10 | 76091222 | **A** | T | -0.195 | 0.043 | 6.0x10-06 | -0.086 | 0.036 | 1.6x10-02 | -0.131 | 0.028 | 2.0x10-06 |
| rs7103884 | FGF3 | 11 | 69372742 | **T** | C | -0.195 | 0.043 | 7.4x10-06 | -0.09 | 0.035 | 1.1x10-02 | -0.132 | 0.027 | 1.5x10-06 |
| rs421007 | KRT6B | 12 | 51137112 | **G** | T | -0.115 | 0.043 | 7.5x10-03 | -0.132 | 0.035 | 1.5x10-04 | -0.125 | 0.027 | 3.7x10-06 |
| rs10877809 | FAM19A2 | 12 | 60873839 | **A** | G | -0.134 | 0.048 | 5.5x10-03 | -0.177 | 0.046 | 1.3x10-04 | -0.156 | 0.033 | 2.7x10-06 |
| rs17105278 | RAD51L1 | 14 | 67798232 | **C** | T | -0.122 | 0.049 | 1.3x10-02 | -0.157 | 0.039 | 4.4x10-05 | -0.144 | 0.03 | 2.1x10-06 |
| rs12708948 | IRX5 | 16 | 53635746 | **T** | C | -0.183 | 0.046 | 8.0x10-05 | -0.128 | 0.044 | 3.5x10-03 | -0.154 | 0.032 | 1.4x10-06 |
| rs11860308 | CES8,UNQ440/PRO873 | 16 | 65593020 | **C** | T | -0.238 | 0.082 | 3.8x10-03 | -0.206 | 0.055 | 1.7x10-04 | -0.216 | 0.046 | 2.2x10-06 |
| rs2429369 | HSF5 | 17 | 53856419 | **G** | A | -0.196 | 0.045 | 1.1x10-05 | -0.118 | 0.037 | 1.3x10-03 | -0.15 | 0.028 | 1.4x10-07 |
| rs7502947 | FAM33A | 17 | 54584498 | **A** | G | -0.182 | 0.045 | 4.4x10-05 | -0.128 | 0.036 | 4.3x10-04 | -0.15 | 0.028 | 1.1x10-07 |
| rs1993104 | FPR1 | 19 | 56932061 | **C** | T | -0.107 | 0.049 | 2.8x10-02 | -0.184 | 0.043 | 1.7x10-05 | -0.151 | 0.032 | 2.8x10-06 |

**Supplementary Table 1: SNPs showing suggestive association in the meta-analysis for 'age at first tooth' ($5x10^{-6}$> p >$5x10^{-8}$). Variants reported are ≥ 500kb apart. The P-value for each cohort is corrected for gestational age and sex. ALSPAC was also corrected for age at measurement. P-values were calculated using a fixed effects inverse variance meta-analysis. All alleles refer to the forward strand. Positions of SNPs reported correspond to HapMap release II build 36. The effect allele (A1) is defined as the allele associated with earlier tooth eruption**

| SNP | GENE REGION/LOCUS | CHR | BP | A1 | A2 | EFFECT ALSPAC | SE ALSPAC | PVALUE ALSPAC | EFFECT NFBC | SE NFBC | PVALUE NFBC | EFFECT META | SE META | PVALUE META |
|---|---|---|---|---|---|---|---|---|---|---|---|---|---|---|
| rs6704363 | TMEM9 | 1 | 199363006 | **A** | G | 0.08 | 0.032 | 1.2x10-02 | 0.156 | 0.037 | 2.9x10-05 | 0.111 | 0.024 | 3.8x10-06 |
| rs183780 | EDAR | 2 | 108961614 | **T** | C | 0.115 | 0.036 | 1.5x10-03 | 0.174 | 0.044 | 7.1x10-05 | 0.139 | 0.028 | 6.3x10-07 |
| rs6967145 | IGF2BP3 | 7 | 23483993 | **G** | C | 0.081 | 0.031 | 9.9x10-03 | 0.179 | 0.038 | 2.3x10-06 | 0.121 | 0.024 | 5.8x10-07 |
| rs2390854 | STK31 | 7 | 23984902 | **C** | T | 0.064 | 0.035 | 6.5x10-02 | 0.234 | 0.043 | 4.7x10-08 | 0.132 | 0.027 | 1.1x10-06 |
| rs11768140 | MPP6 | 7 | 24518518 | **A** | G | 0.119 | 0.038 | 1.7x10-03 | 0.148 | 0.042 | 4.8x10-04 | 0.132 | 0.028 | 3.1x10-06 |
| rs1445570 | PAG1 | 8 | 82002247 | **A** | G | 0.134 | 0.031 | 1.2x10-05 | 0.078 | 0.037 | 3.7x10-02 | 0.111 | 0.024 | 2.6x10-06 |
| rs12379293 | ZCCHC6 | 9 | 88264170 | **A** | C | 0.152 | 0.036 | 1.9x10-05 | 0.103 | 0.042 | 1.4x10-02 | 0.132 | 0.027 | 1.2x10-06 |
| rs451722 | BC093087 | 9 | 89011338 | **G** | A | 0.092 | 0.035 | 7.5x10-03 | 0.159 | 0.04 | 8.7x10-05 | 0.121 | 0.026 | 4.6x10-06 |
| rs7103884 | FGF3 | 11 | 69372742 | **T** | C | 0.178 | 0.031 | 1.0x10-08 | 0.058 | 0.037 | 1.1x10-01 | 0.128 | 0.024 | 6.8x10-08 |
| rs11173690 | FAM19A2 | 12 | 59671837 | **C** | G | 0.142 | 0.039 | 2.4x10-04 | 0.132 | 0.051 | 1.0x10-02 | 0.138 | 0.031 | 7.5x10-06 |
| rs17732182 | PPM1H | 12 | 61312398 | **T** | C | 0.165 | 0.04 | 3.9x10-05 | 0.202 | 0.058 | 5.6x10-04 | 0.177 | 0.033 | 9.0x10-08 |
| rs7146941 | PAX9 | 14 | 36180966 | **A** | C | 0.1 | 0.032 | 1.9x10-03 | 0.129 | 0.037 | 4.9x10-04 | 0.112 | 0.024 | 3.7x10-06 |
| rs1956529 | RAD51L1 | 14 | 67858677 | **C** | T | 0.079 | 0.032 | 1.3x10-02 | 0.176 | 0.037 | 2.0x10-06 | 0.12 | 0.024 | 6.4x10-07 |
| rs4131946 | AF275804 | 16 | 53419975 | **A** | T | 0.114 | 0.034 | 8.7x10-04 | 0.127 | 0.039 | 1.1x10-03 | 0.12 | 0.026 | 3.1x10-06 |
| rs400688 | SLC25A11 | 17 | 4780674 | **G** | A | 0.137 | 0.04 | 6.3x10-04 | 0.139 | 0.042 | 9.3x10-04 | 0.138 | 0.029 | 2.0x10-06 |
| rs4130668 | DNAH2 | 17 | 7680389 | **T** | C | 0.128 | 0.037 | 6.1x10-04 | 0.133 | 0.038 | 4.1x10-04 | 0.13 | 0.027 | 8.6x10-07 |
| rs2680700 | RNF43 | 17 | 53795541 | **T** | G | 0.122 | 0.032 | 1.1x10-04 | 0.138 | 0.036 | 1.3x10-04 | 0.129 | 0.024 | 5.6x10-08 |
| rs2240899 | FLJ35776,DLGAP1 | 18 | 3585011 | **C** | G | 0.147 | 0.031 | 2.9x10-06 | 0.066 | 0.044 | 1.4x10-01 | 0.12 | 0.026 | 2.8x10-06 |

**Supplementary Table 2: SNPs showing suggestive association in the meta-analysis for 'number of teeth' ($5x10^{-6} > p > 5x10^{-8}$) . Variants reported are ≥ 500kb apart. The P-value for each cohort is corrected for gestational age and sex. ALSPAC was also corrected for age at measurement. P-values were calculated using a fixed effects inverse variance meta-analysis. All alleles refer to the forward strand. Positions of SNPs reported correspond to HapMap release II build 36. The effect allele (A1) is defined as the allele associated with an increase in the number of teeth.**

| | | | AGE AT FIRST TOOTH | | | NUMBER OF TEETH | | |
|---|---|---|---|---|---|---|---|---|
| **PRIMARY SNP** | **LOCUS** | **SIGNIFICANCE THRESHOLD CONDITIONAL ANALYSES** | **MOST STRONGLY ASSOCIATED SNP AFTER CONDITIONING ON PRIMARY SNP** | **P VALUE OF SNP BEFORE CONDITIONAL ANALYSIS** | **P VALUE OF SNP AFTER CONDITIONAL ANALYSIS** | **MOST STRONGLY ASSOCIATED SNP AFTER CONDITIONING ON PRIMARY SNP** | **P VALUE OF SNP BEFORE CONDITIONAL ANALYSIS** | **P VALUE OF SNP AFTER CONDITIONAL ANALYSIS** |
| rs8080944 | *KCNJ2 KCNJ16* | $4.5x10^{-4}$ | rs11077486 | $6.201x10^{-05}$ | $2.024x10^{-05}$ | rs9788982 | $3.413x10^{-09}$ | $8.103x10^{-07}$ |
| rs11796357 | *FAM155E – EDA* | $1.0x10^{-3}$ | rs2520397 | $4.800x10-^{16}$ | $8.449x10^{-06}$ | rs2804391 | $1.254x10^{-06}$ | $9.654x10^{-07}$ |
| rs17563 | *BMP4* | $2.5x10^{-4}$ | rs1951867 | $8.000x10^{-03}$ | $8.000x10^{-05}$ | rs1458991 | $7.854x10^{-03}$ | $4.813x10-^{07}$ |
| rs1994969 | *IGF2BP1* | $6.5x10^{-4}$ | rs8069452 | $3.588x10^{-06}$ | $8.221x10^{-06}$ | rs9894411 | $9.298x10^{-07}$ | $1.770x10^{-05}$ |
| rs12229918 | *MSRB3* | $4.3x10^{-4}$ | rs1870566 | $6.000x10^{-03}$ | $4.000x10^{-03}$ | rs1976274 | $2.348x10^{-09}$ | $1.892x10^{-05}$ |
| rs6568401 | *6q21* | $3.8x10^{-4}$ | rs6913299 | $9.000x10^{-03}$ | $4.000x10^{-03}$ | rs3935361 | $2.751x10-^{03}$ | $3.478x10^{-03}$ |
| rs4937076 | *CDON* | $3.5x10^{-4}$ | rs533239 | $1.400x10^{-02}$ | $1.100x10^{-02}$ | rs600281 | $4.994x10^{-03}$ | $1.917x10^{-03}$ |
| rs7924176 | *ADK VCL AP3M1* | $1.6x10^{-3}$ | rs11597376 | $1.000x10^{-03}$ | $6.000x10^{-03}$ | rs7075347 | $9.271x10^{-03}$ | $7.457x10^{-02}$ |
| rs17101923 | *HMGA2* | $4.5x10^{-4}$ | rs1472259 | $1.222x10^{-05}$ | $3.454x10^{-06}$ | rs1472259 | $6.322x10^{-05}$ | $2.228x10^{-05}$ |
| rs10740993 | *CACNB2* | $4.9x10^{-4}$ | rs12359352 | $4.159x10^{-07}$ | $6.377x10^{-04}$ | rs7901587 | $3.536x10^{-07}$ | $1.000x10^{-03}$ |
| rs1799922 | *CALU/OPN1SW* | $7.7x10^{-4}$ | rs3807306 | $8.848x10^{-07}$ | $1.337x10-^{02}$ | rs339072 | $4.90x10^{-04}$ | $1.600x10^{-02}$ |
| rs10932688 | *2q35* | $2.7x10^{-4}$ | rs12474719 | $6.092x10-^{03}$ | $3.627x10^{-03}$ | rs12474719 | $2.839x10^{-05}$ | $1.000x10^{-03}$ |
| rs997154 | *AJUBA/C14orf93* | $2.5x10^{-4}$ | rs1263663 | $6.064x10^{-03}$ | $5.274x10^{-03}$ | rs178752 | $1.000x10^{-03}$ | $1.000x10^{-03}$ |
| rs9316505 | *DLEU7* | $1.1x10^{-4}$ | rs2050936 | $3.062x10^{-03}$ | $8.437x10^{-03}$ | rs2812245 | $2.603x10^{-02}$ | $4.135x10^{-02}$ |
| rs412000 | *TEX15/RAD51C* | 7.2x10-4 | rs3785492 | $4.891x10^{-06}$ | $1.955x10^{-02}$ | rs7215531 | $7.626x10^{-03}$ | $1.238x10^{-03}$ |

**Supplementary Table 3**: Results for the conditional analysis in each genome-wide significant region.

| MARKER | GENE/LOCUS | ALLELES | FREQ1 | EFFECT | STDERR | PVALUE |
|---|---|---|---|---|---|---|
| **rs17101923** | *HMGA2* | **G**/T | 0.19 | 0.1281 | 0.0206 | $4.7x10^{-10}$ |
| **rs7924176** | *ADK VCL AP3M1* | **A**/G | 0.58 | 0.0937 | 0.0157 | $2.5x10^{-9}$ |
| **rs10932688** | 2q35 | **G**/C | 0.72 | 0.0802 | 0.0179 | $7.7x10^{-6}$ |
| **rs10740993** | *CACNB2* | **C**/T | 0.43 | 0.0619 | 0.0155 | $6.6x10^{-5}$ |
| **rs17563** | *BMP4* | **G**/A | 0.39 | 0.0528 | 0.0162 | 0.0011 |
| **rs6568401** | 6q21 | **C**/T | 0.74 | 0.0586 | 0.0187 | 0.0017 |
| **rs12229918** | *MSRB3* | **C**/G | 0.36 | 0.0405 | 0.0162 | 0.0126 |

**Supplementary Table 4**: Results for pleotropic analysis of genome wide significance P<5x10-8 in meta-analysis for secondary dentition (Geller et al). All alleles refer to the forward strand. Positions of SNPs reported correspond to HapMap release II build 36. The Increaser allele is defined in bold.

| HEIGHT Stage 1 & 2 | | | | | | | ALSPAC AGE AT FIRST TOOTH | | | | ALSPAC NUMBER OF TEETH | | NFBC AGE AT FIRST TOOTH | | | | NFBC NUMBER OF TEETH | | META AGE AT FIRST TOOTH | | | META NUMBER OF TEETH | | |
|---|---|---|---|---|---|---|---|---|---|---|---|---|---|---|---|---|---|---|---|---|---|---|---|---|
| **SNP** | **CHR** | **POS** | **GENE** | **A1/A2** | **B** | **P** | **A1** | **A2** | **B** | **P** | **B** | **P** | **A1** | **A2** | **B** | **P** | **B** | **P** | **A1** | **B** | **P** | **A1** | **B** | **P** |
| rs425277 | 1 | 2059032 | PRKCZ | T/C | 0.022 | 2.1x10-08 | C | T | -0.019 | 6.9x10-01 | 0.013 | 6.9x10-01 | T | C | 0.047 | 2.3x10-01 | 0.024 | 5.4x10-01 | T | 0.036 | 2.3x10-01 | C | 0.002 | 9.3x10-01 |
| rs2284746 | 1 | 17179262 | MFAP2 | C/G | -0.04 | 3.9x10-29 | C | G | 0.002 | 9.6x10-01 | -0.001 | 9.6x10-01 | G | C | 0.023 | 5.2x10-01 | 0.017 | 6.4x10-01 | G | -0.013 | 6.4x10-01 | C | -0.008 | 7.3x10-01 |
| rs1738475 | 1 | 23409478 | HTR1D | C/G | 0.025 | 3.0x10-12 | C | G | -0.012 | 7.8x10-01 | -0.033 | 2.9x10-01 | G | C | 0.024 | 5.0x10-01 | -0.004 | 9.1x10-01 | C | -0.019 | 4.9x10-01 | C | -0.018 | 4.5x10-01 |
| rs4601530 | 1 | 24916698 | CLIC4 | T/C | -0.028 | 2.2x10-12 | C | T | -0.012 | 8.0x10-01 | 0.045 | 2.0x10-01 | T | C | 0.078 | 3.7x10-02 | -0.054 | 1.7x10-01 | T | 0.054 | 7.1x10-02 | T | -0.049 | 6.1x10-02 |
| rs7532866 | 1 | 26614131 | LIN28 | A/G | 0.021 | 3.4x10-08 | A | G | 0.014 | 7.6x10-01 | -0.003 | 9.3x10-01 | G | A | 0.002 | 9.6x10-01 | -0.010 | 8.0x10-01 | A | 0.004 | 8.8x10-01 | G | 0.003 | 9.2x10-01 |
| rs2154319 | 1 | 41518357 | SCMH1 | T/C | -0.03 | 1.8x10-12 | C | T | -0.003 | 9.6x10-01 | -0.005 | 8.9x10-01 | T | C | -0.032 | 4.9x10-01 | -0.001 | 9.9x10-01 | C | -0.016 | 6.3x10-01 | T | 0.003 | 9.2x10-01 |
| rs17391694 | 1 | 78396214 | GIPC2 | T/C | 0.042 | 1.7x10-11 | C | T | 0.156 | 9.9x10-03 | -0.069 | 1.1x10-01 | T | C | -0.012 | 8.7x10-01 | -0.010 | 8.9x10-01 | T | -0.096 | 3.8x10-02 | T | 0.049 | 1.9x10-01 |
| rs6699417 | 1 | 88896031 | PKN2 | T/C | 0.021 | 5.0x10-09 | C | T | -0.034 | 4.3x10-01 | 0.048 | 1.2x10-01 | T | C | 0.017 | 6.4x10-01 | -0.045 | 2.4x10-01 | T | 0.024 | 3.8x10-01 | T | -0.047 | 5.1x10-02 |
| rs10874746 | 1 | 93096559 | RPL5 | T/C | -0.024 | 6.7x10-11 | C | T | 0.020 | 6.6x10-01 | 0.065 | 4.1x10-02 | T | C | 0.108 | 2.5x10-03 | -0.064 | 8.4x10-02 | C | 0.058 | 3.8x10-02 | T | -0.064 | 7.4x10-03 |
| rs9428104 | 1 | 118657110 | SPAG17 | A/G | -0.041 | 5.6x10-23 | A | G | -0.012 | 8.1x10-01 | -0.017 | 6.3x10-01 | G | A | -0.007 | 8.7x10-01 | 0.102 | 1.6x10-02 | A | -0.001 | 9.8x10-01 | A | -0.051 | 5.7x10-02 |
| rs11205277 | 1 | 148159496 | SF3B4 | A/G | -0.046 | 4.8x10-32 | A | G | 0.054 | 4.2x10-01 | -0.050 | 2.9x10-01 | G | A | -0.004 | 9.2x10-01 | -0.008 | 8.4x10-01 | A | 0.016 | 6.3x10-01 | A | -0.015 | 6.1x10-01 |
| rs17346452 | 1 | 170319910 | DNM3 | T/C | -0.04 | 1.4x10-23 | C | T | -0.012 | 8.0x10-01 | -0.004 | 9.0x10-01 | T | C | 0.046 | 2.8x10-01 | 0.011 | 8.0x10-01 | T | 0.031 | 3.3x10-01 | T | 0.007 | 8.0x10-01 |
| rs1325598 | 1 | 175058872 | PAPPA2 | A/G | -0.022 | 1.1x10-09 | A | G | 0.101 | 1.8x10-02 | -0.028 | 3.5x10-01 | G | A | -0.057 | 1.0x10-01 | 0.055 | 1.2x10-01 | A | 0.075 | 5.7x10-03 | A | -0.040 | 8.9x10-02 |
| rs1046934 | 1 | 182290152 | TSEN15 | A/C | -0.044 | 2.1x10-31 | A | C | 0.038 | 4.0x10-01 | -0.038 | 2.4x10-01 | C | A | -0.027 | 4.5x10-01 | -0.013 | 7.3x10-01 | A | 0.031 | 2.7x10-01 | A | -0.016 | 5.1x10-01 |
| rs10863936 | 1 | 210304421 | DTL | A/G | -0.021 | 1.9x10-09 | A | G | -0.051 | 2.3x10-01 | 0.011 | 7.2x10-01 | G | A | -0.036 | 3.0x10-01 | 0.009 | 8.1x10-01 | G | 0.001 | 9.6x10-01 | A | 0.003 | 9.1x10-01 |
| rs6684205 | 1 | 216676325 | TGFB2 | A/G | -0.028 | 1.5x10-12 | A | G | 0.072 | 1.4x10-01 | -0.031 | 3.8x10-01 | G | A | -0.035 | 3.5x10-01 | -0.029 | 4.7x10-01 | A | 0.049 | 1.0x10-01 | A | -0.005 | 8.6x10-01 |
| rs11118346 | 1 | 217810342 | LYPLAL1 | T/C | -0.025 | 1.9x10-12 | C | T | 0.000 | 1.0x10+00 | -0.013 | 6.6x10-01 | T | C | 0.065 | 6.0x10-02 | -0.026 | 4.6x10-01 | T | 0.040 | 1.4x10-01 | C | -0.003 | 8.8x10-01 |
| rs10799445 | 1 | 225978506 | JMJD4 | A/C | 0.032 | 2.4x10-13 | A | C | 0.127 | 1.4x10-02 | -0.067 | 6.8x10-02 | C | A | 0.013 | 7.3x10-01 | -0.028 | 4.8x10-01 | A | 0.037 | 2.3x10-01 | A | -0.023 | 3.9x10-01 |
| rs4665736 | 2 | 25041103 | DNAJC27 | T/C | 0.029 | 7.3x10-16 | C | T | 0.022 | 6.1x10-01 | 0.047 | 1.3x10-01 | T | C | 0.044 | 2.1x10-01 | -0.016 | 6.6x10-01 | C | 0.018 | 5.1x10-01 | T | -0.034 | 1.5x10-01 |
| rs6714546 | 2 | 33214929 | LTBP1 | A/G | -0.026 | 1.6x10-09 | A | G | -0.015 | 7.6x10-01 | -0.008 | 8.2x10-01 | G | A | -0.025 | 5.5x10-01 | 0.087 | 4.6x10-02 | G | 0.007 | 8.1x10-01 | A | -0.037 | 1.6x10-01 |
| rs17511102 | 2 | 37814117 | CDC42EP3 | A/T | -0.06 | 1.6x10-18 | A | T | -0.137 | 1.2x10-01 | 0.007 | 9.1x10-01 | T | A | -0.040 | 5.6x10-01 | 0.071 | 3.3x10-01 | A | -0.029 | 6.0x10-01 | T | -0.026 | 5.8x10-01 |
| rs2341459 | 2 | 44621706 | C2orf34 | T/C | 0.025 | 7.9x10-10 | C | T | 0.002 | 9.6x10-01 | 0.015 | 6.8x10-01 | T | C | -0.080 | 2.5x10-02 | -0.016 | 6.6x10-01 | T | -0.054 | 6.5x10-02 | T | -0.015 | 5.5x10-01 |
| rs12474201 | 2 | 46774789 | SOCS5 | A/G | 0.028 | 2.6x10-13 | A | G | -0.026 | 5.7x10-01 | -0.006 | 8.6x10-01 | G | A | -0.076 | 4.9x10-02 | 0.061 | 1.3x10-01 | G | 0.033 | 2.7x10-01 | A | -0.027 | 2.8x10-01 |
| rs3791675 | 2 | 55964813 | EFEMP1 | T/C | -0.053 | 2.5x10-35 | C | T | -0.060 | 2.4x10-01 | 0.052 | 1.5x10-01 | T | C | 0.014 | 7.3x10-01 | -0.006 | 8.9x10-01 | T | 0.032 | 3.2x10-01 | T | -0.032 | 2.4x10-01 |
| rs11684404 | 2 | 88705737 | EIF2AK3 | T/C | -0.028 | 9.9x10-14 | C | T | 0.070 | 1.2x10-01 | -0.077 | 1.8x10-02 | T | C | -0.005 | 8.8x10-01 | 0.037 | 3.1x10-01 | T | -0.030 | 2.8x10-01 | T | 0.060 | 1.4x10-02 |
| rs7567288 | 2 | 134151294 | NCKAP5 | T/C | -0.032 | 2.1x10-12 | C | T | 0.032 | 5.6x10-01 | -0.030 | 4.5x10-01 | T | C | -0.018 | 6.5x10-01 | -0.037 | 3.6x10-01 | T | -0.022 | 4.8x10-01 | C | -0.003 | 9.3x10-01 |
| rs7567851 | 2 | 178392966 | PDE11A | C/G | 0.037 | 3.3x10-08 | C | G | -0.011 | 8.9x10-01 | 0.053 | 3.6x10-01 | G | C | -0.063 | 4.0x10-01 | 0.053 | 4.9x10-01 | G | 0.029 | 6.0x10-01 | C | 0.015 | 7.4x10-01 |
| rs1351164 | 2 | 217980143 | TNS1 | T/C | 0.034 | 2.1x10-14 | C | T | -0.039 | 4.8x10-01 | 0.038 | 3.2x10-01 | T | C | 0.096 | 1.5x10-02 | -0.003 | 9.3x10-01 | T | 0.076 | 1.7x10-02 | T | -0.022 | 4.4x10-01 |
| rs12470505 | 2 | 219616613 | CCDC108/IHH | T/G | 0.041 | 8.9x10-12 | G | T | -0.089 | 2.1x10-01 | 0.007 | 8.9x10-01 | T | G | 0.045 | 4.8x10-01 | 0.000 | 1.0x10+00 | T | 0.065 | 1.7x10-01 | T | -0.004 | 9.2x10-01 |
| rs2629046 | 2 | 224755988 | SERPINE2 | T/C | 0.024 | 7.9x10-12 | C | T | -0.139 | 1.3x10-03 | 0.041 | 1.8x10-01 | T | C | 0.029 | 4.0x10-01 | 0.023 | 5.2x10-01 | T | 0.072 | 7.9x10-03 | T | -0.014 | 5.6x10-01 |
| rs2580816 | 2 | 232506210 | NPPC | T/C | -0.045 | 5.8x10-22 | C | T | 0.022 | 6.9x10-01 | -0.004 | 9.3x10-01 | T | C | -0.059 | 2.6x10-01 | 0.082 | 1.3x10-01 | T | -0.041 | 2.7x10-01 | T | 0.030 | 3.4x10-01 |
| rs12694997 | 2 | 241911659 | "SEPT2" | A/G | -0.024 | 1.2x10-08 | A | G | 0.012 | 8.2x10-01 | -0.028 | 4.5x10-01 | G | A | -0.041 | 3.2x10-01 | -0.008 | 8.5x10-01 | A | 0.030 | 3.6x10-01 | A | -0.013 | 6.5x10-01 |
| rs2597513 | 3 | 13530836 | HDAC11 | T/C | -0.036 | 7.4x10-10 | C | T | 0.056 | 4.2x10-01 | 0.035 | 4.8x10-01 | T | C | -0.085 | 1.9x10-01 | 0.037 | 5.9x10-01 | T | -0.072 | 1.3x10-01 | T | -0.010 | 8.0x10-01 |
| rs13088462 | 3 | 51046753 | DOCK3 | T/C | -0.052 | 3.8x10-10 | C | T | -0.110 | 2.6x10-01 | 0.119 | 8.3x10-02 | T | C | 0.117 | 1.2x10-01 | -0.065 | 4.1x10-01 | T | 0.115 | 5.4x10-02 | T | -0.095 | 6.4x10-02 |

| | | | | | | | | | | | | | | | | | | | | | | | | |
|---|---|---|---|---|---|---|---|---|---|---|---|---|---|---|---|---|---|---|---|---|---|---|---|---|
| rs2336725 | 3 | 53093779 | RTF1 | T/C | -0.027 | 9.7x10-13 | C | T | 0.040 | 3.5x10-01 | 0.010 | 7.4x10-01 | T | C | 0.025 | 5.1x10-01 | 0.021 | 6.1x10-01 | T | -0.003 | 9.1x10-01 | C | 0.001 | 9.5x10-01 |
| rs9835332 | 3 | 56642722 | C3orf63 | C/G | -0.026 | 5.3x10-13 | C | G | -0.028 | 5.1x10-01 | 0.028 | 3.5x10-01 | G | C | 0.020 | 5.7x10-01 | -0.053 | 1.4x10-01 | C | -0.023 | 3.9x10-01 | C | 0.039 | 9.6x10-02 |
| rs17806888 | 3 | 67499012 | SUCLG2 | T/C | 0.036 | 2.1x10-09 | C | T | 0.046 | 4.8x10-01 | 0.000 | 1.0x10+00 | T | C | -0.118 | 2.1x10-01 | 0.056 | 5.7x10-01 | T | -0.070 | 1.9x10-01 | C | 0.010 | 8.1x10-01 |
| rs9863706 | 3 | 72520103 | RYBP | T/C | -0.031 | 4.1x10-13 | C | T | 0.033 | 5.3x10-01 | -0.020 | 5.9x10-01 | T | C | 0.019 | 6.7x10-01 | -0.010 | 8.3x10-01 | T | -0.004 | 9.2x10-01 | T | 0.009 | 7.7x10-01 |
| rs6439167 | 3 | 130533446 | C3orf47 | T/C | -0.034 | 8.9x10-15 | C | T | -0.049 | 3.4x10-01 | 0.028 | 4.5x10-01 | T | C | 0.030 | 5.1x10-01 | -0.017 | 7.3x10-01 | T | 0.038 | 2.6x10-01 | T | -0.024 | 4.2x10-01 |
| rs9844666 | 3 | 137456906 | PCCB | A/G | -0.024 | 3.5x10-09 | A | G | 0.024 | 6.2x10-01 | -0.022 | 5.3x10-01 | G | A | -0.036 | 3.8x10-01 | -0.022 | 6.1x10-01 | A | 0.031 | 3.3x10-01 | A | -0.005 | 8.6x10-01 |
| rs724016 | 3 | 142588260 | ZBTB38 | A/G | -0.07 | 3.1x10-86 | A | G | -0.009 | 8.4x10-01 | -0.028 | 3.6x10-01 | G | A | 0.015 | 6.7x10-01 | 0.039 | 2.8x10-01 | A | -0.013 | 6.5x10-01 | A | -0.033 | 1.6x10-01 |
| rs572169 | 3 | 173648421 | GHSR | T/C | 0.033 | 2.8x10-18 | C | T | -0.076 | 1.0x10-01 | 0.088 | 7.7x10-03 | T | C | -0.058 | 1.2x10-01 | 0.017 | 6.6x10-01 | C | -0.006 | 8.3x10-01 | T | -0.043 | 8.6x10-02 |
| rs720390 | 3 | 187031377 | IGF2BP2 | A/G | 0.029 | 1.9x10-14 | A | G | -0.012 | 7.9x10-01 | -0.016 | 6.1x10-01 | G | A | 0.017 | 6.4x10-01 | -0.005 | 9.0x10-01 | A | -0.015 | 5.9x10-01 | A | -0.008 | 7.6x10-01 |
| rs2247341 | 4 | 1671115 | SLBP/FGFR3 | A/G | 0.025 | 1.5x10-11 | A | G | -0.050 | 2.7x10-01 | 0.031 | 3.5x10-01 | G | A | -0.045 | 2.0x10-01 | 0.034 | 3.6x10-01 | G | 0.010 | 7.2x10-01 | A | 0.002 | 9.3x10-01 |
| rs6449353 | 4 | 17642586 | LCORL | T/C | 0.075 | 7.1x10-46 | C | T | -0.067 | 2.9x10-01 | -0.008 | 8.6x10-01 | T | C | -0.050 | 4.0x10-01 | 0.025 | 6.8x10-01 | T | 0.005 | 9.0x10-01 | T | 0.014 | 7.0x10-01 |
| rs17081935 | 4 | 57518233 | POLR2B | T/C | 0.03 | 3.7x10-11 | C | T | 0.002 | 9.8x10-01 | 0.022 | 5.8x10-01 | T | C | 0.077 | 6.1x10-02 | -0.051 | 2.3x10-01 | C | 0.049 | 1.4x10-01 | T | -0.035 | 2.2x10-01 |
| rs7697556 | 4 | 73734177 | ADAMTS3 | T/C | 0.028 | 2.0x10-14 | C | T | 0.009 | 8.4x10-01 | 0.028 | 3.6x10-01 | T | C | 0.029 | 4.2x10-01 | -0.076 | 3.7x10-02 | C | 0.014 | 6.2x10-01 | T | -0.048 | 4.1x10-02 |
| rs788867 | 4 | 82369030 | PRKG2/BMP3 | T/G | -0.043 | 8.9x10-28 | G | T | 0.063 | 1.8x10-01 | -0.045 | 1.7x10-01 | T | G | 0.056 | 1.2x10-01 | -0.018 | 6.4x10-01 | G | 0.012 | 6.9x10-01 | T | 0.018 | 4.8x10-01 |
| rs10010325 | 4 | 106325802 | TET2 | A/C | 0.024 | 3.9x10-11 | A | C | 0.017 | 6.9x10-01 | -0.036 | 2.3x10-01 | C | A | -0.003 | 9.2x10-01 | 0.043 | 2.4x10-01 | A | 0.009 | 7.4x10-01 | A | -0.039 | 9.2x10-02 |
| rs7689420 | 4 | 145787802 | HHIP | T/C | -0.073 | 6.2x10-51 | C | T | 0.022 | 7.0x10-01 | -0.051 | 2.1x10-01 | T | C | -0.076 | 9.0x10-02 | 0.057 | 2.3x10-01 | T | -0.055 | 1.2x10-01 | T | 0.054 | 8.1x10-02 |
| rs955748 | 4 | 184452669 | WWC2 | A/G | -0.023 | 4.4x10-08 | A | G | -0.002 | 9.7x10-01 | -0.032 | 3.6x10-01 | G | A | 0.037 | 3.6x10-01 | -0.029 | 4.8x10-01 | A | -0.023 | 4.7x10-01 | A | -0.007 | 8.0x10-01 |
| rs1173727 | 5 | 32866278 | NPR3 | T/C | 0.034 | 1.6x10-21 | C | T | 0.034 | 4.4x10-01 | -0.037 | 2.4x10-01 | T | C | 0.021 | 5.4x10-01 | -0.035 | 3.4x10-01 | T | 0.000 | 9.9x10-01 | T | 0.007 | 7.7x10-01 |
| rs11958779 | 5 | 55037656 | SLC38A9 | A/G | -0.027 | 1.8x10-12 | A | G | -0.014 | 7.7x10-01 | -0.003 | 9.2x10-01 | G | A | -0.029 | 4.3x10-01 | 0.056 | 1.5x10-01 | G | 0.013 | 6.6x10-01 | A | -0.026 | 3.1x10-01 |
| rs10037512 | 5 | 88390431 | MEF2C | T/C | 0.032 | 2.0x10-18 | C | T | 0.027 | 5.3x10-01 | -0.012 | 7.0x10-01 | T | C | 0.038 | 2.8x10-01 | 0.021 | 5.7x10-01 | C | 0.012 | 6.7x10-01 | T | 0.016 | 5.1x10-01 |
| rs13177718 | 5 | 108141243 | FER | T/C | -0.04 | 3.0x10-08 | C | T | 0.022 | 7.9x10-01 | 0.057 | 3.3x10-01 | T | C | 0.081 | 3.1x10-01 | -0.007 | 9.3x10-01 | C | 0.031 | 6.0x10-01 | T | -0.040 | 3.9x10-01 |
| rs1582931 | 5 | 122685098 | CEP120 | A/G | -0.023 | 1.5x10-10 | A | G | -0.064 | 1.6x10-01 | 0.051 | 1.1x10-01 | G | A | 0.057 | 1.2x10-01 | -0.066 | 8.0x10-02 | A | -0.060 | 3.5x10-02 | A | 0.058 | 1.9x10-02 |
| rs274546 | 5 | 131727766 | SLC22A5 | A/G | -0.029 | 7.3x10-16 | A | G | -0.013 | 7.7x10-01 | 0.032 | 3.1x10-01 | G | A | -0.037 | 2.9x10-01 | 0.005 | 8.9x10-01 | G | 0.018 | 5.2x10-01 | A | 0.016 | 5.0x10-01 |
| rs526896 | 5 | 134384604 | PITX1 | T/G | 0.03 | 2.3x10-13 | G | T | -0.017 | 7.4x10-01 | -0.035 | 3.3x10-01 | T | G | -0.048 | 2.5x10-01 | 0.034 | 4.3x10-01 | G | -0.022 | 5.0x10-01 | T | 0.035 | 2.1x10-01 |
| rs4282339 | 5 | 168188818 | SLIT3 | A/G | -0.036 | 6.6x10-16 | A | G | -0.018 | 7.3x10-01 | 0.024 | 5.2x10-01 | G | A | -0.021 | 6.2x10-01 | -0.028 | 5.3x10-01 | G | 0.005 | 8.7x10-01 | A | 0.026 | 3.7x10-01 |
| rs12153391 | 5 | 171136043 | FBXW11 | A/C | -0.03 | 3.6x10-12 | A | C | -0.043 | 3.9x10-01 | 0.025 | 5.0x10-01 | C | A | 0.096 | 1.6x10-02 | -0.077 | 6.4x10-02 | A | -0.076 | 1.6x10-02 | A | 0.047 | 8.4x10-02 |
| rs889014 | 5 | 172916720 | BOD1 | T/C | -0.03 | 9.4x10-16 | C | T | -0.054 | 2.3x10-01 | 0.002 | 9.6x10-01 | T | C | 0.038 | 2.8x10-01 | 0.033 | 3.6x10-01 | T | 0.044 | 1.1x10-01 | C | 0.014 | 5.7x10-01 |
| rs422421 | 5 | 176449932 | FGFR4/NSD1 | T/C | -0.031 | 1.1x10-12 | C | T | -0.035 | 5.0x10-01 | -0.021 | 5.7x10-01 | T | C | -0.009 | 8.5x10-01 | 0.049 | 3.3x10-01 | T | 0.011 | 7.6x10-01 | T | 0.031 | 3.0x10-01 |
| rs6879260 | 5 | 179663620 | GFPT2 | T/C | -0.022 | 1.6x10-09 | C | T | 0.003 | 9.4x10-01 | 0.069 | 2.8x10-02 | T | C | 0.035 | 3.1x10-01 | -0.015 | 6.8x10-01 | C | 0.021 | 4.5x10-01 | T | -0.046 | 5.3x10-02 |
| rs3812163 | 6 | 7670759 | BMP6 | A/T | -0.036 | 1.2x10-23 | A | T | -0.018 | 6.8x10-01 | 0.046 | 1.3x10-01 | T | A | -0.051 | 1.4x10-01 | 0.059 | 1.0x10-01 | T | 0.024 | 3.8x10-01 | A | 0.002 | 9.3x10-01 |
| rs1047014 | 6 | 19949472 | ID4 | T/C | -0.032 | 1.8x10-13 | C | T | 0.058 | 2.4x10-01 | 0.015 | 6.6x10-01 | T | C | 0.009 | 8.4x10-01 | -0.021 | 6.4x10-01 | T | -0.019 | 5.5x10-01 | T | -0.018 | 5.2x10-01 |
| rs806794 | 6 | 26308656 | Histone cluster | A/G | 0.052 | 1.2x10-39 | A | G | 0.097 | 4.7x10-02 | -0.029 | 4.0x10-01 | G | A | 0.017 | 6.2x10-01 | -0.053 | 1.4x10-01 | A | 0.025 | 2.8x10-02 | G | 0.011 | 2.5x10-02 |
| rs3129109 | 6 | 29192211 | OR2J3 | T/C | -0.032 | 2.4x10-17 | C | T | 0.015 | 7.4x10-01 | -0.037 | 2.5x10-01 | T | C | 0.021 | 5.6x10-01 | -0.045 | 2.3x10-01 | C | 0.007 | 8.1x10-01 | T | 0.003 | 9.1x10-01 |
| rs2256183 | 6 | 31488508 | MICA | A/G | 0.04 | 7.8x10-29 | A | G | 0.003 | 9.4x10-01 | -0.012 | 6.9x10-01 | G | A | -0.047 | 1.8x10-01 | 0.077 | 3.6x10-02 | A | 0.030 | 2.8x10-01 | A | -0.039 | 9.8x10-02 |
| rs6457620 | 6 | 32771977 | HLA locus | C/G | -0.029 | 2.1x10-16 | C | G | 0.062 | 1.4x10-01 | -0.028 | 3.5x10-01 | G | C | -0.025 | 4.8x10-01 | 0.020 | 5.9x10-01 | C | 0.040 | 2.7x10-02 | C | -0.025 | 2.3x10-02 |
| rs2780226 | 6 | 34307070 | HMGA1 | T/C | -0.076 | 8.1x10-28 | C | T | 0.097 | 2.0x10-01 | 0.012 | 8.2x10-01 | T | C | -0.019 | 8.1x10-01 | 0.046 | 5.7x10-01 | T | -0.058 | 2.8x10-01 | C | 0.006 | 8.9x10-01 |
| rs6457821 | 6 | 35510783 | PPARD/FANCE | A/C | -0.104 | 2.1x10-12 | A | C | 0.012 | 9.4x10-01 | 0.051 | 6.4x10-01 | - | - | - | - | - | - | A | 0.012 | 9.4x10-01 | A | 0.051 | 6.4x10-01 |

| | | | | | | | | | | | | | | | | | | | | | | | | |
|---|---|---|---|---|---|---|---|---|---|---|---|---|---|---|---|---|---|---|---|---|---|---|---|---|
| rs310405 | 6 | 81857081 | FAM46A | A/G | 0.026 | 2.2x10-13 | A | G | 0.010 | 8.1x10-01 | -0.018 | 5.6x10-01 | G | A | -0.019 | 6.0x10-01 | 0.046 | 2.2x10-01 | A | 0.015 | 5.8x10-01 | A | -0.029 | 2.2x10-01 |
| rs7759938 | 6 | 105485647 | LIN28B | T/C | -0.045 | 8.3x10-31 | C | T | 0.027 | 5.7x10-01 | 0.004 | 8.9x10-01 | T | C | -0.062 | 9.1x10-02 | 0.010 | 8.0x10-01 | T | -0.048 | 9.3x10-02 | C | 0.002 | 9.4x10-01 |
| rs1046943 | 6 | 109890634 | ZBTB24 | A/G | 0.02 | 2.5x10-08 | A | G | 0.053 | 2.3x10-01 | -0.042 | 1.8x10-01 | G | A | -0.003 | 9.4x10-01 | -0.007 | 8.5x10-01 | A | 0.022 | 4.2x10-01 | G | -0.021 | 3.7x10-01 |
| rs961764 | 6 | 117628849 | VGLL2 | C/G | -0.024 | 1.3x10-11 | C | G | -0.117 | 6.7x10-03 | 0.080 | 9.8x10-03 | G | C | 0.011 | 7.6x10-01 | 0.034 | 3.6x10-01 | C | -0.053 | 5.1x10-02 | C | 0.033 | 1.7x10-01 |
| rs1490384 | 6 | 126892853 | C6orf173 | T/C | 0.034 | 3.9x10-21 | C | T | 0.235 | 3.6x10-08 | -0.035 | 2.4x10-01 | T | C | -0.083 | 1.8x10-02 | 0.036 | 3.2x10-01 | T | -0.144 | 1.0x10-07 | T | 0.036 | 1.2x10-01 |
| rs6569648 | 6 | 130390812 | L3MBTL3 | T/C | -0.04 | 1.1x10-21 | C | T | 0.115 | 2.1x10-02 | -0.033 | 3.5x10-01 | T | C | -0.054 | 2.0x10-01 | -0.009 | 8.3x10-01 | T | -0.080 | 1.4x10-02 | T | 0.016 | 5.6x10-01 |
| rs7763064 | 6 | 142838982 | GPR126 | A/G | -0.048 | 1.1x10-33 | A | G | -0.047 | 3.2x10-01 | 0.032 | 3.5x10-01 | G | A | 0.048 | 2.4x10-01 | -0.100 | 1.7x10-02 | A | -0.047 | 1.2x10-01 | A | 0.058 | 2.6x10-02 |
| rs543650 | 6 | 152152636 | ESR1 | T/G | -0.034 | 1.2x10-17 | G | T | -0.022 | 6.2x10-01 | 0.006 | 8.6x10-01 | T | G | 0.017 | 6.3x10-01 | -0.013 | 7.1x10-01 | T | 0.019 | 4.9x10-01 | T | -0.009 | 7.0x10-01 |
| rs9456307 | 6 | 158849430 | TULP4 | A/T | -0.048 | 2.2x10-09 | A | T | 0.155 | 7.9x10-02 | -0.079 | 2.1x10-01 | T | A | -0.086 | 4.8x10-01 | 0.068 | 5.9x10-01 | A | 0.131 | 6.6x10-02 | A | -0.077 | 1.7x10-01 |
| rs798489 | 7 | 2768329 | GNA12 | T/C | -0.048 | 1.9x10-33 | C | T | 0.018 | 7.1x10-01 | -0.048 | 1.6x10-01 | T | C | 0.022 | 5.6x10-01 | 0.028 | 4.7x10-01 | C | 0.007 | 8.1x10-01 | T | 0.039 | 1.3x10-01 |
| rs4470914 | 7 | 19583047 | TWISTNB | T/C | 0.029 | 9.2x10-10 | C | T | 0.007 | 8.9x10-01 | 0.020 | 6.0x10-01 | T | C | 0.072 | 1.6x10-01 | -0.074 | 1.6x10-01 | C | 0.034 | 3.5x10-01 | T | -0.038 | 2.1x10-01 |
| rs12534093 | 7 | 23469499 | IGF2BP3 | A/T | -0.034 | 2.0x10-14 | A | T | 0.103 | 4.7x10-02 | -0.094 | 9.8x10-03 | T | A | -0.100 | 2.3x10-02 | 0.159 | 4.8x10-04 | A | 0.101 | 2.6x10-03 | A | -0.120 | 2.7x10-05 |
| rs1708299 | 7 | 28156471 | JAZF1 | A/G | 0.04 | 5.8x10-25 | A | G | 0.019 | 6.8x10-01 | 0.000 | 9.9x10-01 | G | A | -0.057 | 1.3x10-01 | 0.014 | 7.2x10-01 | A | 0.043 | 1.5x10-01 | A | -0.006 | 8.1x10-01 |
| rs6959212 | 7 | 38094851 | STARD3NL | T/C | -0.024 | 1.6x10-09 | C | T | -0.011 | 8.0x10-01 | 0.000 | 1.0x10+00 | T | C | 0.015 | 7.2x10-01 | 0.036 | 4.0x10-01 | T | 0.013 | 6.7x10-01 | C | 0.013 | 6.1x10-01 |
| rs42235 | 7 | 92086012 | CDK6 | T/C | 0.057 | 7.7x10-47 | C | T | 0.012 | 7.9x10-01 | 0.067 | 4.7x10-02 | T | C | -0.033 | 4.1x10-01 | -0.076 | 7.0x10-02 | T | -0.025 | 4.3x10-01 | T | -0.070 | 7.2x10-03 |
| rs822552 | 7 | 148281567 | PDIA4 | C/G | -0.025 | 2.6x10-08 | C | G | 0.010 | 8.5x10-01 | -0.036 | 3.6x10-01 | G | C | -0.028 | 5.6x10-01 | 0.051 | 2.9x10-01 | C | 0.020 | 5.7x10-01 | C | -0.042 | 1.7x10-01 |
| rs2110001 | 7 | 150147955 | TMEM176A | C/G | -0.031 | 3.3x10-13 | C | G | -0.009 | 8.5x10-01 | 0.016 | 6.4x10-01 | G | C | 0.002 | 9.6x10-01 | -0.025 | 5.3x10-01 | C | -0.005 | 8.7x10-01 | C | 0.020 | 4.5x10-01 |
| rs1013209 | 8 | 24172249 | ADAM28 | T/C | -0.025 | 1.6x10-09 | C | T | 0.009 | 8.6x10-01 | -0.023 | 5.3x10-01 | T | C | -0.004 | 9.1x10-01 | 0.031 | 4.3x10-01 | T | -0.006 | 8.5x10-01 | T | 0.027 | 3.2x10-01 |
| rs7460090 | 8 | 57356717 | SDR16C5 | T/C | 0.058 | 8.2x10-27 | C | T | 0.034 | 6.1x10-01 | -0.074 | 1.1x10-01 | T | C | -0.076 | 1.3x10-01 | 0.098 | 6.7x10-02 | T | -0.061 | 1.3x10-01 | T | 0.084 | 1.7x10-02 |
| rs6473015 | 8 | 78341040 | PEX2 | A/C | -0.029 | 6.9x10-13 | A | C | 0.044 | 3.5x10-01 | -0.026 | 4.4x10-01 | C | A | 0.058 | 1.7x10-01 | -0.075 | 8.9x10-02 | C | -0.013 | 6.9x10-01 | C | 0.011 | 6.7x10-01 |
| rs6470764 | 8 | 130794847 | GSDMC | T/C | -0.05 | 1.7x10-28 | C | T | -0.124 | 2.0x10-02 | 0.125 | 8.0x10-04 | T | C | -0.032 | 5.5x10-01 | 0.002 | 9.7x10-01 | T | 0.047 | 2.1x10-01 | T | -0.086 | 5.8x10-03 |
| rs12680655 | 8 | 135706519 | ZFAT | C/G | 0.028 | 1.6x10-14 | C | G | 0.031 | 4.8x10-01 | 0.003 | 9.2x10-01 | G | C | -0.008 | 8.1x10-01 | 0.070 | 5.1x10-02 | C | 0.017 | 5.3x10-01 | G | -0.028 | 2.3x10-01 |
| rs7864648 | 9 | 16358732 | BNC2 | T/G | 0.022 | 2.1x10-08 | G | T | 0.026 | 6.1x10-01 | -0.012 | 7.3x10-01 | T | G | -0.030 | 4.2x10-01 | 0.033 | 3.9x10-01 | T | -0.028 | 3.4x10-01 | T | 0.022 | 4.0x10-01 |
| rs11144688 | 9 | 77732106 | PCSK5 | A/G | -0.049 | 9.6x10-12 | A | G | -0.074 | 5.0x10-01 | 0.008 | 9.2x10-01 | G | A | 0.061 | 4.9x10-01 | 0.113 | 2.1x10-01 | A | -0.066 | 3.4x10-01 | G | -0.044 | 4.6x10-01 |
| rs7853377 | 9 | 85742025 | C9orf64 | A/G | -0.024 | 4.5x10-08 | A | G | 0.006 | 9.1x10-01 | -0.050 | 1.9x10-01 | G | A | 0.063 | 1.6x10-01 | -0.072 | 1.2x10-01 | G | -0.034 | 3.2x10-01 | A | -0.002 | 9.6x10-01 |
| rs8181166 | 9 | 88306448 | ZCCHC6 | C/G | 0.026 | 2.7x10-12 | C | G | -0.034 | 4.3x10-01 | 0.078 | 1.2x10-02 | G | C | 0.039 | 2.7x10-01 | -0.084 | 2.2x10-02 | C | -0.037 | 1.8x10-01 | C | 0.080 | 7.0x10-04 |
| rs2778031 | 9 | 90025546 | SPIN1 | T/C | 0.031 | 9.0x10-13 | C | T | 0.068 | 1.8x10-01 | 0.031 | 3.9x10-01 | T | C | 0.085 | 6.4x10-02 | -0.015 | 7.5x10-01 | C | 0.016 | 6.4x10-01 | T | -0.025 | 3.8x10-01 |
| rs9969804 | 9 | 94468941 | IPPK | A/C | 0.03 | 7.7x10-17 | A | C | 0.023 | 6.0x10-01 | 0.011 | 7.1x10-01 | C | A | 0.038 | 2.7x10-01 | -0.027 | 4.4x10-01 | C | -0.014 | 5.9x10-01 | A | 0.018 | 4.3x10-01 |
| rs1257763 | 9 | 95933766 | PTPDC1 | A/G | 0.069 | 9.9x10-10 | A | G | -0.075 | 5.8x10-01 | 0.022 | 8.2x10-01 | G | A | 0.165 | 1.8x10-01 | -0.110 | 4.0x10-01 | A | -0.124 | 1.7x10-01 | A | 0.053 | 5.0x10-01 |
| rs473902 | 9 | 97296056 | PTCH1/FANCC | T/G | 0.065 | 2.3x10-17 | G | T | -0.033 | 7.5x10-01 | 0.012 | 8.7x10-01 | T | G | 0.077 | 2.8x10-01 | -0.015 | 8.4x10-01 | T | 0.063 | 2.9x10-01 | T | -0.014 | 8.0x10-01 |
| rs7027110 | 9 | 108638867 | ZNF462 | A/G | 0.031 | 2.3x10-13 | A | G | 0.030 | 5.5x10-01 | 0.031 | 3.9x10-01 | G | A | -0.130 | 2.3x10-03 | 0.073 | 9.8x10-02 | A | 0.088 | 6.8x10-03 | G | -0.010 | 7.2x10-01 |
| rs1468758 | 9 | 112846903 | LPAR1 | T/C | -0.026 | 1.4x10-09 | C | T | 0.036 | 4.7x10-01 | -0.048 | 1.8x10-01 | T | C | -0.040 | 3.4x10-01 | 0.004 | 9.3x10-01 | T | -0.039 | 2.3x10-01 | T | 0.030 | 2.7x10-01 |
| rs751543 | 9 | 118162163 | PAPPA | T/C | 0.026 | 6.5x10-10 | C | T | 0.066 | 2.0x10-01 | -0.025 | 5.0x10-01 | T | C | 0.036 | 3.9x10-01 | 0.027 | 5.4x10-01 | T | -0.005 | 8.8x10-01 | T | 0.026 | 3.6x10-01 |

| | | | | | | | | | | | | | | | | | | | | | | | | |
|---|---|---|---|---|---|---|---|---|---|---|---|---|---|---|---|---|---|---|---|---|---|---|---|---|
| rs11599750 | 10 | 101795432 | CPN1 | T/C | -0.028 | 1.6x10-13 | C | T | -0.022 | 6.1x10-01 | 0.011 | 7.2x10-01 | T | C | -0.016 | 6.8x10-01 | -0.002 | 9.5x10-01 | T | 0.001 | 9.8x10-01 | T | -0.008 | 7.5x10-01 |
| rs2237886 | 11 | 2767307 | KCNQ1 | T/C | 0.046 | 2.2x10-13 | C | T | 0.044 | 5.3x10-01 | -0.032 | 5.2x10-01 | T | C | -0.078 | 2.2x10-01 | 0.129 | 5.1x10-02 | T | -0.062 | 1.9x10-01 | T | 0.068 | 9.0x10-02 |
| rs7926971 | 11 | 12654616 | TEAD1 | A/G | -0.023 | 4.4x10-10 | A | G | 0.011 | 7.9x10-01 | -0.006 | 8.5x10-01 | G | A | -0.033 | 3.4x10-01 | -0.004 | 9.2x10-01 | A | 0.024 | 3.7x10-01 | A | -0.002 | 9.4x10-01 |
| rs1330 | 11 | 17272605 | NUCB2 | T/C | 0.022 | 4.9x10-09 | C | T | 0.044 | 3.2x10-01 | 0.001 | 9.8x10-01 | T | C | 0.043 | 2.5x10-01 | -0.027 | 4.8x10-01 | C | 0.008 | 7.9x10-01 | T | -0.012 | 6.4x10-01 |
| rs10838801 | 11 | 48054856 | PTPRJ/SLC39A13 | A/G | -0.027 | 3.5x10-12 | A | G | -0.009 | 8.5x10-01 | -0.013 | 6.9x10-01 | G | A | -0.049 | 2.0x10-01 | 0.009 | 8.3x10-01 | G | 0.026 | 3.8x10-01 | A | -0.011 | 6.5x10-01 |
| rs1814175 | 11 | 49515748 | FOLH1 | T/C | 0.022 | 1.6x10-08 | C | T | 0.012 | 7.9x10-01 | 0.014 | 6.7x10-01 | T | C | -0.021 | 5.6x10-01 | -0.001 | 9.8x10-01 | T | -0.018 | 5.3x10-01 | T | -0.008 | 7.3x10-01 |
| rs5017948 | 11 | 51270794 | OR4A5 | A/T | 0.027 | 3.1x10-08 | A | T | -0.062 | 2.6x10-01 | 0.018 | 6.5x10-01 | T | A | 0.081 | 9.6x10-02 | 0.010 | 8.4x10-01 | A | -0.073 | 4.7x10-02 | A | 0.007 | 8.2x10-01 |
| rs3782089 | 11 | 65093395 | SSSCA1 | T/C | -0.058 | 3.6x10-13 | C | T | -0.050 | 5.5x10-01 | 0.064 | 2.8x10-01 | T | C | -0.139 | 5.2x10-02 | 0.032 | 6.7x10-01 | C | -0.058 | 2.8x10-01 | T | -0.027 | 5.6x10-01 |
| rs7112925 | 11 | 66582736 | RHOD | T/C | -0.023 | 9.0x10-10 | C | T | 0.000 | 1.0x10+00 | 0.033 | 3.0x10-01 | T | C | 0.004 | 9.2x10-01 | 0.007 | 8.6x10-01 | C | 0.002 | 9.4x10-01 | T | -0.017 | 4.8x10-01 |
| rs634552 | 11 | 74959700 | SERPINH1 | T/G | 0.039 | 3.5x10-13 | G | T | -0.190 | 2.6x10-03 | 0.071 | 1.1x10-01 | T | G | -0.029 | 5.3x10-01 | 0.011 | 8.2x10-01 | T | 0.046 | 2.1x10-01 | T | -0.032 | 3.2x10-01 |
| rs494459 | 11 | 118079885 | TREH | T/C | 0.02 | 1.7x10-08 | C | T | 0.085 | 5.1x10-02 | -0.020 | 5.3x10-01 | T | C | 0.029 | 4.1x10-01 | -0.022 | 5.4x10-01 | T | -0.016 | 5.6x10-01 | T | 0.002 | 9.4x10-01 |
| rs654723 | 11 | 128091365 | FLI1 | A/C | 0.025 | 3.6x10-11 | A | C | 0.107 | 1.8x10-02 | -0.041 | 2.0x10-01 | C | A | -0.031 | 4.3x10-01 | 0.007 | 8.6x10-01 | A | 0.063 | 3.2x10-02 | A | -0.028 | 2.7x10-01 |
| rs2856321 | 12 | 11747040 | ETV6 | A/G | -0.029 | 4.5x10-15 | A | G | -0.038 | 3.9x10-01 | 0.039 | 2.2x10-01 | G | A | 0.013 | 7.1x10-01 | -0.040 | 2.8x10-01 | A | -0.023 | 4.1x10-01 | A | 0.040 | 1.0x10-01 |
| rs10770705 | 12 | 20748734 | SLCO1C1 | A/C | 0.033 | 8.0x10-18 | A | C | 0.004 | 9.4x10-01 | -0.004 | 9.1x10-01 | C | A | -0.041 | 2.9x10-01 | 0.062 | 1.2x10-01 | A | 0.026 | 3.9x10-01 | A | -0.027 | 2.8x10-01 |
| rs2638953 | 12 | 28425682 | CCDC91 | C/G | 0.032 | 6.7x10-17 | C | G | -0.023 | 6.1x10-01 | 0.006 | 8.6x10-01 | G | C | 0.014 | 6.9x10-01 | -0.035 | 3.7x10-01 | C | -0.018 | 5.3x10-01 | C | 0.018 | 4.8x10-01 |
| rs2066807 | 12 | 55026949 | STAT2 | C/G | -0.054 | 1.0x10-13 | C | G | 0.095 | 2.8x10-01 | -0.108 | 8.1x10-02 | G | C | 0.018 | 8.3x10-01 | -0.017 | 8.3x10-01 | C | 0.034 | 5.7x10-01 | C | -0.062 | 2.1x10-01 |
| rs1351394 | 12 | 64638093 | HMGA2 | T/C | 0.06 | 1.7x10-65 | C | T | 0.149 | 5.5x10-04 | -0.151 | 9.8x10-07 | T | C | -0.128 | 2.6x10-04 | 0.134 | 2.4x10-04 | T | -0.136 | 5.3x10-07 | T | 0.144 | 1.0x10-09 |
| rs10748128 | 12 | 68113925 | FRS2 | T/G | 0.038 | 2.1x10-20 | G | T | -0.107 | 1.8x10-02 | 0.023 | 4.7x10-01 | T | G | -0.004 | 9.2x10-01 | -0.018 | 6.7x10-01 | T | 0.046 | 1.3x10-01 | T | -0.022 | 4.0x10-01 |
| rs11107116 | 12 | 92502635 | SOCS2 | T/G | 0.052 | 1.4x10-34 | G | T | 0.006 | 9.1x10-01 | 0.034 | 3.5x10-01 | T | G | -0.016 | 6.9x10-01 | 0.007 | 8.6x10-01 | T | -0.012 | 7.0x10-01 | T | -0.016 | 5.5x10-01 |
| rs7971536 | 12 | 100897919 | CCDC53/GNPTAB | A/T | -0.028 | 8.2x10-14 | A | T | -0.013 | 7.7x10-01 | 0.025 | 4.3x10-01 | T | A | -0.008 | 8.2x10-01 | 0.004 | 9.1x10-01 | A | 0.000 | 9.9x10-01 | A | 0.013 | 5.8x10-01 |
| rs11830103 | 12 | 122389499 | SBNO1 | A/G | -0.035 | 3.9x10-15 | A | G | -0.003 | 9.6x10-01 | 0.000 | 9.9x10-01 | G | A | -0.057 | 1.7x10-01 | 0.073 | 9.4x10-02 | G | 0.035 | 2.9x10-01 | A | -0.032 | 2.7x10-01 |
| rs7332115 | 13 | 32045548 | PDS5B/BRCA2 | T/G | -0.023 | 5.5x10-10 | G | T | -0.099 | 2.6x10-02 | 0.033 | 2.9x10-01 | T | G | -0.042 | 2.3x10-01 | 0.056 | 1.2x10-01 | T | 0.012 | 6.6x10-01 | G | 0.005 | 8.3x10-01 |
| rs3118905 | 13 | 50003335 | DLEU7 | A/G | -0.056 | 1.1x10-45 | A | G | -0.009 | 8.4x10-01 | 0.014 | 6.8x10-01 | G | A | -0.033 | 3.8x10-01 | 0.029 | 4.6x10-01 | G | 0.017 | 5.6x10-01 | G | -0.005 | 8.5x10-01 |
| rs7319045 | 13 | 90822575 | GPC5 | A/G | 0.025 | 1.2x10-11 | A | G | -0.046 | 3.0x10-01 | 0.059 | 6.2x10-02 | G | A | -0.001 | 9.8x10-01 | -0.036 | 3.4x10-01 | A | -0.018 | 5.3x10-01 | A | 0.049 | 4.1x10-02 |
| rs1950500 | 14 | 23900690 | NFATC4 | T/C | 0.034 | 2.2x10-18 | C | T | -0.066 | 1.5x10-01 | 0.008 | 8.0x10-01 | T | C | -0.007 | 8.6x10-01 | -0.022 | 5.8x10-01 | T | 0.023 | 4.4x10-01 | T | -0.014 | 5.9x10-01 |
| rs2093210 | 14 | 60027032 | SIX6 | T/C | -0.032 | 6.2x10-17 | C | T | 0.027 | 5.5x10-01 | -0.036 | 2.6x10-01 | T | C | -0.017 | 6.7x10-01 | -0.057 | 1.7x10-01 | T | -0.021 | 4.8x10-01 | T | 0.001 | 9.6x10-01 |
| rs1570106 | 14 | 67882868 | RAD51L1 | T/C | -0.026 | 8.1x10-09 | C | T | 0.136 | 1.2x10-02 | -0.119 | 2.2x10-03 | T | C | -0.126 | 3.5x10-03 | 0.164 | 2.4x10-04 | T | -0.130 | 1.2x10-04 | T | 0.139 | 2.3x10-06 |
| rs862034 | 14 | 74060499 | LTBP2 | A/G | -0.028 | 7.3x10-14 | A | G | 0.001 | 9.7x10-01 | -0.020 | 5.2x10-01 | G | A | -0.034 | 3.5x10-01 | 0.010 | 7.9x10-01 | A | 0.021 | 4.6x10-01 | A | -0.016 | 5.0x10-01 |
| rs7155279 | 14 | 91555634 | TRIP11 | T/G | -0.024 | 1.4x10-10 | G | T | -0.033 | 4.6x10-01 | -0.028 | 3.9x10-01 | T | G | 0.010 | 8.0x10-01 | -0.037 | 3.4x10-01 | T | 0.019 | 5.1x10-01 | T | 0.001 | 9.6x10-01 |
| rs16964211 | 15 | 49317787 | CYP19A1 | A/G | -0.05 | 1.7x10-09 | A | G | -0.122 | 2.4x10-01 | -0.035 | 6.3x10-01 | G | A | -0.077 | 2.6x10-01 | -0.028 | 7.0x10-01 | G | 0.017 | 7.7x10-01 | A | -0.003 | 9.6x10-01 |
| rs7178424 | 15 | 60167551 | C2CD4A | T/C | -0.021 | 5.6x10-09 | C | T | 0.010 | 8.2x10-01 | 0.010 | 7.5x10-01 | T | C | 0.034 | 3.5x10-01 | -0.011 | 7.8x10-01 | C | 0.016 | 5.7x10-01 | T | -0.010 | 6.7x10-01 |
| rs10152591 | 15 | 67835211 | TLE3 | A/C | 0.041 | 2.7x10-10 | A | C | 0.015 | 8.2x10-01 | 0.019 | 6.9x10-01 | C | A | 0.060 | 4.3x10-01 | -0.001 | 9.9x10-01 | C | -0.018 | 7.2x10-01 | A | 0.014 | 7.3x10-01 |
| rs12902421 | 15 | 69948457 | MYO9A | T/C | -0.062 | 2.9x10-08 | C | T | -0.001 | 1.0x10+00 | 0.100 | 2.6x10-01 | T | C | 0.054 | 7.0x10-01 | -0.029 | 8.4x10-01 | T | 0.024 | 8.0x10-01 | T | -0.081 | 2.9x10-01 |

| | | | | | | | | | | | | | | | | | | | | | | | | |
|---|---|---|---|---|---|---|---|---|---|---|---|---|---|---|---|---|---|---|---|---|---|---|---|---|
| rs4965598 | 15 | 98577137 | ADAMTS17 | T/C | -0.028 | 4.3x10-13 | C | T | -0.017 | 7.1x10-01 | 0.001 | 9.6x10-01 | T | C | 0.071 | 7.9x10-02 | -0.094 | 2.6x10-02 | T | 0.047 | 1.2x10-01 | T | -0.036 | 1.6x10-01 |
| rs11648796 | 16 | 732191 | NARFL | A/G | -0.034 | 1.2x10-13 | A | G | 0.048 | 3.8x10-01 | -0.045 | 2.5x10-01 | G | A | -0.016 | 6.8x10-01 | 0.002 | 9.6x10-01 | A | 0.027 | 4.0x10-01 | A | -0.025 | 3.9x10-01 |
| rs26868 | 16 | 2189377 | CASKIN1 | A/T | 0.034 | 9.0x10-17 | A | T | -0.032 | 4.6x10-01 | 0.039 | 2.1x10-01 | T | A | -0.012 | 7.5x10-01 | -0.015 | 6.9x10-01 | A | -0.007 | 8.2x10-01 | A | 0.030 | 2.2x10-01 |
| rs1659127 | 16 | 14295806 | MKL2 | A/G | 0.027 | 1.1x10-11 | A | G | 0.088 | 6.5x10-02 | -0.008 | 8.1x10-01 | G | A | -0.009 | 8.2x10-01 | -0.012 | 7.7x10-01 | A | 0.040 | 1.8x10-01 | G | 0.000 | 9.9x10-01 |
| rs8052560 | 16 | 87304743 | CTU2/GALNS | A/C | 0.029 | 3.3x10-08 | A | C | -0.025 | 6.7x10-01 | 0.039 | 3.4x10-01 | C | A | 0.017 | 7.6x10-01 | -0.045 | 4.3x10-01 | A | -0.021 | 6.1x10-01 | A | 0.041 | 2.2x10-01 |
| rs4640244 | 17 | 21224816 | KCNJ12 | A/G | 0.024 | 2.3x10-08 | A | G | 0.046 | 2.9x10-01 | -0.037 | 2.4x10-01 | G | A | -0.060 | 1.4x10-01 | 0.059 | 1.6x10-01 | A | 0.053 | 7.4x10-02 | A | -0.045 | 7.6x10-02 |
| rs3110496 | 17 | 24941897 | ANKRD13B | A/G | -0.022 | 7.3x10-09 | A | G | -0.021 | 6.5x10-01 | -0.021 | 5.2x10-01 | G | A | -0.042 | 2.2x10-01 | 0.022 | 5.5x10-01 | G | 0.019 | 4.9x10-01 | A | -0.021 | 3.8x10-01 |
| rs3764419 | 17 | 26188149 | ATAD5/RNF135 | A/C | -0.035 | 1.8x10-21 | A | C | 0.015 | 7.3x10-01 | 0.020 | 5.2x10-01 | C | A | -0.013 | 7.2x10-01 | 0.054 | 1.4x10-01 | A | 0.014 | 6.2x10-01 | C | -0.011 | 6.4x10-01 |
| rs17780086 | 17 | 27367395 | LRRC37B | A/G | 0.028 | 2.6x10-08 | A | G | 0.014 | 8.3x10-01 | -0.017 | 7.2x10-01 | G | A | -0.049 | 3.6x10-01 | 0.134 | 1.7x10-02 | A | 0.035 | 4.0x10-01 | A | -0.064 | 7.3x10-02 |
| rs1043515 | 17 | 34175722 | PIP4K2B | A/G | -0.023 | 2.9x10-10 | A | G | 0.102 | 1.8x10-02 | -0.080 | 9.1x10-03 | G | A | -0.023 | 5.1x10-01 | 0.055 | 1.2x10-01 | A | 0.053 | 4.7x10-02 | A | -0.069 | 2.9x10-03 |
| rs4986172 | 17 | 40571807 | ACBD4 | T/C | -0.032 | 2.3x10-16 | C | T | -0.022 | 6.3x10-01 | 0.044 | 1.8x10-01 | T | C | 0.002 | 9.6x10-01 | 0.013 | 7.1x10-01 | T | 0.009 | 7.4x10-01 | T | -0.018 | 4.5x10-01 |
| rs2072153 | 17 | 44745013 | ZNF652 | C/G | 0.021 | 3.5x10-08 | C | G | -0.005 | 9.2x10-01 | 0.026 | 4.4x10-01 | G | C | 0.065 | 9.7x10-02 | -0.061 | 1.3x10-01 | C | -0.040 | 1.8x10-01 | C | 0.040 | 1.2x10-01 |
| rs4605213 | 17 | 46599746 | NME2 | C/G | 0.021 | 2.7x10-08 | C | G | -0.053 | 2.5x10-01 | 0.021 | 5.2x10-01 | G | C | 0.034 | 3.8x10-01 | -0.025 | 5.3x10-01 | C | -0.042 | 1.6x10-01 | C | 0.023 | 3.7x10-01 |
| rs227724 | 17 | 52133816 | NOG | A/T | -0.03 | 7.4x10-15 | A | T | -0.167 | 3.1x10-04 | 0.028 | 3.9x10-01 | T | A | 0.034 | 3.4x10-01 | 0.048 | 2.0x10-01 | A | -0.085 | 3.0x10-03 | T | -0.005 | 8.5x10-01 |
| rs2079795 | 17 | 56851431 | TBX2 | T/C | 0.04 | 2.1x10-24 | C | T | 0.014 | 7.6x10-01 | -0.006 | 8.4x10-01 | T | C | 0.025 | 5.4x10-01 | -0.014 | 7.3x10-01 | C | 0.008 | 7.9x10-01 | C | -0.001 | 9.6x10-01 |
| rs2665838 | 17 | 59320197 | CSH1/GH1 | C/G | -0.042 | 5.1x10-25 | C | G | 0.002 | 9.8x10-01 | -0.021 | 5.6x10-01 | G | C | 0.000 | 1.0 | -0.040 | 3.5x10-01 | C | 0.001 | 9.8x10-01 | G | 0.004 | 8.8x10-01 |
| rs11867479 | 17 | 65601802 | KCNJ16/KCNJ2 | T/C | 0.025 | 1.5x10-10 | C | T | -0.030 | 4.9x10-01 | 0.031 | 3.3x10-01 | T | C | 0.074 | 9.0x10-02 | -0.132 | 3.6x10-03 | T | 0.053 | 9.2x10-02 | T | -0.064 | 1.3x10-02 |
| rs4800452 | 18 | 18981609 | CABLES1 | T/C | 0.051 | 4.2x10-30 | C | T | 0.018 | 7.3x10-01 | 0.018 | 6.3x10-01 | T | C | -0.035 | 4.0x10-01 | 0.010 | 8.1x10-01 | T | -0.029 | 3.8x10-01 | T | -0.006 | 8.4x10-01 |
| rs9967417 | 18 | 45213498 | DYM | C/G | -0.038 | 9.3x10-25 | C | G | 0.008 | 8.5x10-01 | -0.030 | 3.3x10-01 | G | C | 0.030 | 4.6x10-01 | 0.002 | 9.6x10-01 | G | -0.012 | 6.8x10-01 | C | -0.020 | 4.2x10-01 |
| rs17782313 | 18 | 56002077 | MC4R | T/C | -0.028 | 3.8x10-11 | C | T | 0.146 | 3.6x10-03 | -0.069 | 5.2x10-02 | T | C | 0.001 | 9.9x10-01 | -0.036 | 4.5x10-01 | T | -0.066 | 5.2x10-02 | T | 0.031 | 2.7x10-01 |
| rs12982744 | 19 | 2128193 | DOT1L | C/G | -0.03 | 3.4x10-16 | C | G | -0.023 | 6.0x10-01 | -0.023 | 4.5x10-01 | G | C | -0.010 | 8.0x10-01 | -0.044 | 2.6x10-01 | C | -0.004 | 8.8x10-01 | G | 0.003 | 9.1x10-01 |
| rs7507204 | 19 | 3379834 | NFIC | C/G | 0.036 | 4.3x10-16 | C | G | -0.012 | 8.3x10-01 | 0.005 | 9.0x10-01 | G | C | -0.002 | 9.7x10-01 | -0.049 | 2.5x10-01 | C | -0.003 | 9.3x10-01 | C | 0.026 | 3.8x10-01 |
| rs891088 | 19 | 7135762 | INSR | A/G | -0.029 | 2.4x10-12 | A | G | -0.029 | 5.5x10-01 | 0.038 | 2.8x10-01 | G | A | -0.025 | 5.1x10-01 | 0.002 | 9.7x10-01 | G | 0.004 | 8.9x10-01 | A | 0.021 | 4.2x10-01 |
| rs4072910 | 19 | 8550031 | ADAMTS10 | C/G | -0.031 | 3.6x10-13 | C | G | 0.017 | 7.2x10-01 | 0.033 | 3.3x10-01 | G | C | -0.002 | 9.5x10-01 | -0.009 | 8.2x10-01 | C | 0.008 | 7.9x10-01 | C | 0.023 | 3.7x10-01 |
| rs2279008 | 19 | 17144303 | MYO9B | T/C | 0.025 | 2.5x10-08 | C | T | 0.031 | 5.3x10-01 | 0.029 | 4.1x10-01 | T | C | -0.079 | 2.9x10-02 | 0.002 | 9.5x10-01 | T | -0.062 | 3.4x10-02 | T | -0.014 | 5.7x10-01 |
| rs17318596 | 19 | 46628935 | ATP5SL | A/G | 0.032 | 5.0x10-16 | A | G | -0.015 | 7.6x10-01 | 0.009 | 8.0x10-01 | G | A | 0.037 | 3.6x10-01 | -0.040 | 3.3x10-01 | A | -0.028 | 3.7x10-01 | A | 0.022 | 4.1x10-01 |
| rs1741344 | 20 | 4049800 | SMOX | T/C | -0.023 | 3.3x10-09 | C | T | 0.065 | 1.5x10-01 | -0.030 | 3.5x10-01 | T | C | 0.046 | 2.4x10-01 | 0.011 | 7.9x10-01 | T | -0.002 | 9.4x10-01 | T | 0.023 | 3.6x10-01 |
| rs2145272 | 20 | 6574218 | BMP2 | A/G | -0.039 | 2.1x10-24 | A | G | 0.037 | 4.1x10-01 | 0.024 | 4.6x10-01 | G | A | -0.077 | 4.8x10-02 | -0.011 | 7.8x10-01 | A | 0.060 | 4.2x10-02 | A | 0.019 | 4.5x10-01 |
| rs7274811 | 20 | 31796842 | ZNF341 | T/G | -0.041 | 5.9x10-22 | G | T | -0.091 | 6.2x10-02 | 0.018 | 6.0x10-01 | T | G | 0.038 | 3.5x10-01 | -0.071 | 9.3x10-02 | T | 0.059 | 5.6x10-02 | T | -0.039 | 1.4x10-01 |
| rs143384 | 20 | 33489170 | GDF5 | A/G | -0.063 | 1.0x10-58 | A | G | 0.005 | 9.2x10-01 | 0.052 | 1.2x10-01 | G | A | -0.037 | 3.1x10-01 | 0.019 | 6.3x10-01 | A | 0.025 | 3.9x10-01 | A | 0.021 | 4.0x10-01 |
| rs237743 | 20 | 47336426 | ZNFX1 | A/G | 0.041 | 1.3x10-20 | A | G | -0.100 | 5.0x10-02 | 0.089 | 1.4x10-02 | G | A | 0.016 | 7.0x10-01 | -0.018 | 6.7x10-01 | A | -0.049 | 1.3x10-01 | A | 0.060 | 3.1x10-02 |
| rs2834442 | 21 | 34612656 | KCNE2 | A/T | 0.026 | 5.1x10-12 | A | T | 0.022 | 6.3x10-01 | 0.002 | 9.5x10-01 | T | A | 0.033 | 3.7x10-01 | -0.050 | 1.8x10-01 | T | -0.011 | 7.0x10-01 | A | 0.022 | 3.7x10-01 |
| rs4821083 | 22 | 31386341 | SYN3 | T/C | 0.031 | 3.1x10-10 | C | T | -0.042 | 4.9x10-01 | 0.044 | 3.1x10-01 | T | C | -0.053 | 2.8x10-01 | -0.034 | 5.0x10-01 | C | -0.016 | 6.8x10-01 | T | -0.040 | 2.3x10-01 |

**Supplementary Table 5: 180 robustly associated height variants from the Lango-Allen et al. (2010) Giant Consortium meta-analysis examining the degree to which these SNPs were associated with ‘age at first tooth’ and ‘number of teeth’. A1 refers to the effect allele in all studies.**

| | | Pathway P value thresholds | | | | | |
|---|---|---|---|---|---|---|---|
| P-value criterion for SNPs | Number of genes | P<0.05 | | P<0.01 | | P<0.001 | |
| | | Number of pathways | P value | Number of pathways | P value | Number of pathways | P value |
| 0.0001 | 118 | 70 | 0.017 | 19 | 0.049 | 4 | 0.053 |
| 0.0005 | 262 | 49 | 0.174 | 16 | 0.089 | 2 | 0.159 |
| 0.001 | 410 | 73 | 0.063 | 16 | 0.110 | 3 | 0.086 |
| 0.005 | 1358 | 157 | 0.004 | 50 | 0.002 | 11 | 0.004 |

**(A)** Number of significantly over-represented pathways in the 'age at first tooth' GWAS.

| | | Pathway P value thresholds | | | | | |
|---|---|---|---|---|---|---|---|
| P-value criterion for SNPs | Number of genes | P<0.05 | | P<0.01 | | P<0.001 | |
| | | Number of pathways | P value | Number of pathways | P value | Number of pathways | P value |
| 0.0001 | 90 | 25 | 0.196 | 6 | 0.258 | 1 | 0.255 |
| 0.0005 | 254 | 26 | 0.546 | 5 | 0.542 | 1 | 0.355 |
| 0.001 | 408 | 56 | 0.146 | 13 | 0.167 | 0 | 1.000 |
| 0.005 | 1272 | 60 | 0.273 | 17 | 0.130 | 0 | 1.000 |

**(B)** Number of significantly over-represented pathways in the 'number of teeth' GWAS.

**Supplementary Table 6**: Summary statistics from ALIGATOR analyses for (a) the 'time to first tooth' GWAS and (b) the 'number of teeth' GWAS . The first column gives the P-value criteria (left-most column) GWAS threshold. The second column displays the number of genes with one or more SNPs with a p-value less than the labelled threshold. Third column displays number of pathways with a p-value less than the labelled threshold, the adjacent P value indicates whether this number is significantly greater than that expected by chance, similarly for the subsequent columns.
